# Erythrocyte-derived extracellular vesicles induce endothelial dysfunction through arginase-1 and oxidative stress in type 2 diabetes

**DOI:** 10.1172/JCI180900

**Published:** 2025-03-20

**Authors:** Aida Collado, Rawan Humoud, Eftychia Kontidou, Maria Eldh, Jasmin Swaich, Allan Zhao, Jiangning Yang, Tong Jiao, Elena Domingo, Emelie Carlestål, Ali Mahdi, John Tengbom, Ákos Végvári, Qiaolin Deng, Michael Alvarsson, Susanne Gabrielsson, Per Eriksson, Zhichao Zhou, John Pernow

**Affiliations:** 1 Division of Cardiology and; 2 Division of Immunology and Respiratory Medicine, Department of Medicine Solna, Karolinska University Hospital, Karolinska Institutet, Stockholm, Sweden.; 3 Center for Molecular Medicine and; 4 Department of Clinical Immunology and Transfusion Medicine, Karolinska University Hospital, Stockholm, Sweden.; 5 KTH Royal Institute of Technology, Stockholm, Sweden.; 6 Department of Physiology and Pharmacology, Karolinska Institutet, Stockholm, Sweden.; 7 Department of Pharmacology, Faculty of Medicine and Odontology, University of Valencia, Valencia, Spain.; 8 Institute of Health Research, University Clinic Hospital of Valencia, Valencia, Spain.; 9 Department of Molecular Medicine and Surgery and; 10 Division of Chemistry I, Department of Medical Biochemistry and Biophysics, Karolinska Institutet, Stockholm, Sweden.; 11 Center for Diabetes, Academic Specialist Center, Health Care Services Stockholm County, Stockholm, Sweden.; 12 Department of Cardiology, Karolinska University Hospital, Stockholm, Sweden.

**Keywords:** Cardiology, Vascular biology, Cardiovascular disease, Diabetes, Endothelial cells

## Abstract

Red blood cells (RBCs) induce endothelial dysfunction in type 2 diabetes (T2D), but the mechanism by which RBCs communicate with the endothelium is unknown. This study tested the hypothesis that extracellular vesicles (EVs) secreted by RBCs act as mediators of endothelial dysfunction in T2D. Despite a lower production of EVs derived from RBCs of T2D patients (T2D RBC-EVs), their uptake by endothelial cells was greater than that of EVs derived from RBCs of healthy individuals (H RBC-EVs). T2D RBC-EVs impaired endothelium-dependent relaxation, and this effect was attenuated following inhibition of arginase in EVs. Inhibition of vascular arginase or oxidative stress also attenuated endothelial dysfunction induced by T2D RBC-EVs. Arginase-1 was detected in RBC-derived EVs, and arginase-1 and oxidative stress were increased in endothelial cells following coincubation with T2D RBC-EVs. T2D RBC-EVs also increased arginase-1 protein in endothelial cells following mRNA silencing and in the endothelium of aortas from endothelial cell arginase-1–knockout mice. It is concluded that T2D-RBCs induce endothelial dysfunction through increased uptake of EVs that transfer arginase-1 from RBCs to the endothelium to induce oxidative stress and endothelial dysfunction. These results shed important light on the mechanism underlying endothelial dysfunction mediated by RBCs in T2D.

## Abstract 2

Vascular injury in type 2 diabetes is caused by extracellular vesicles produced by and transported from red blood cells to the vascular wall.

## Graphical abstract

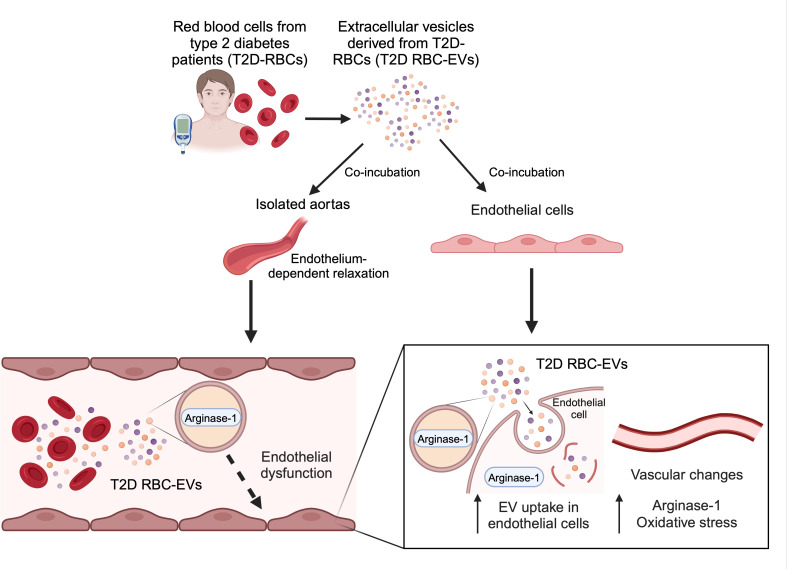

## Introduction

Type 2 diabetes (T2D) is one of the major risk factors for cardiovascular disease (1). Over time, patients with T2D have poorer clinical outcomes and develop multiple cardiovascular complications related to atherosclerosis. The pathogenesis behind the development of atherosclerosis and ischemic heart disease is complex and multifactorial, which explains why specific treatment, beyond glucose-lowering therapy to specifically prevent cardiovascular complications, is currently limited or lacking (2). Thus, there is a need for improved insights into the mechanism behind cardiovascular complications to develop novel treatments that specifically target and prevent the development of cardiovascular disease in patients with T2D.

Existing evidence suggests that vascular endothelial dysfunction, characterized by impaired bioavailability of the vasodilator and antiinflammatory signaling molecule nitric oxide (NO) and increased oxidative stress, occurs in early stages and contributes to cardiovascular disease progression (3). However, the mechanisms driving endothelial dysfunction and, thereby, atherosclerotic cardiovascular progression remain to be identified. Our lab has identified the red blood cell (RBC), an overlooked player in cardiovascular disease, as a key contributor to endothelial dysfunction in T2D (4). RBCs from patients with T2D (T2D-RBCs) induce endothelial and cardiac dysfunction via a mechanism involving upregulation of arginase, which results in attenuated NO bioavailability and formation of reactive oxygen species ROS (4, 5). These observations point to an intriguing mechanism behind cardiovascular injury in T2D mediated by RBC-derived signaling. However, it remains to be clarified how the RBCs transmit their signals to the endothelium, resulting in the dysregulation of endothelial function in T2D.

Extracellular vesicles (EVs) are known to be important mediators of intercellular communication. They consist of a heterogeneous group of subcellular closed membranous structures released by cells in an evolutionarily conserved manner (6). They are involved in a multitude of biological processes through their ability to carry and transfer proteins, metabolites, lipids, and genetic material between different cell types (7, 8). Interestingly, RBCs are one of the major sources of circulating EVs, and RBC-derived EVs may represent a mechanism of intercellular communication both under physiological and pathological conditions (9, 10). However, the functional role of EVs derived from RBCs in the communication between T2D-RBCs and the cardiovascular system has not been explored.

Consequently, we hypothesized that EVs derived from RBCs of patients with T2D carry arginase, enter endothelial cells, and induce endothelial dysfunction. By using a translational approach, we aimed to demonstrate that RBC-derived EVs are important mediators of endothelial dysfunction in T2D through the upregulation of arginase-1 and increased ROS formation.

## Results

### Study population.

Characteristics of the healthy individuals and patients with T2D included for blood sampling and collection of RBCs are shown in Table 1. T2D patients had higher body mass index, systolic and diastolic blood pressure, fasting glucose, glycated hemoglobin, and triglycerides, whereas total cholesterol, high-density lipoprotein, and low-density lipoprotein levels were lower compared with the age-matched healthy control group. There were 7 smokers in the T2D group and 2 in the healthy control group. No medication was taken by any of the healthy participants.

### RBCs from patients with T2D release fewer EVs.

**Figure 1:**
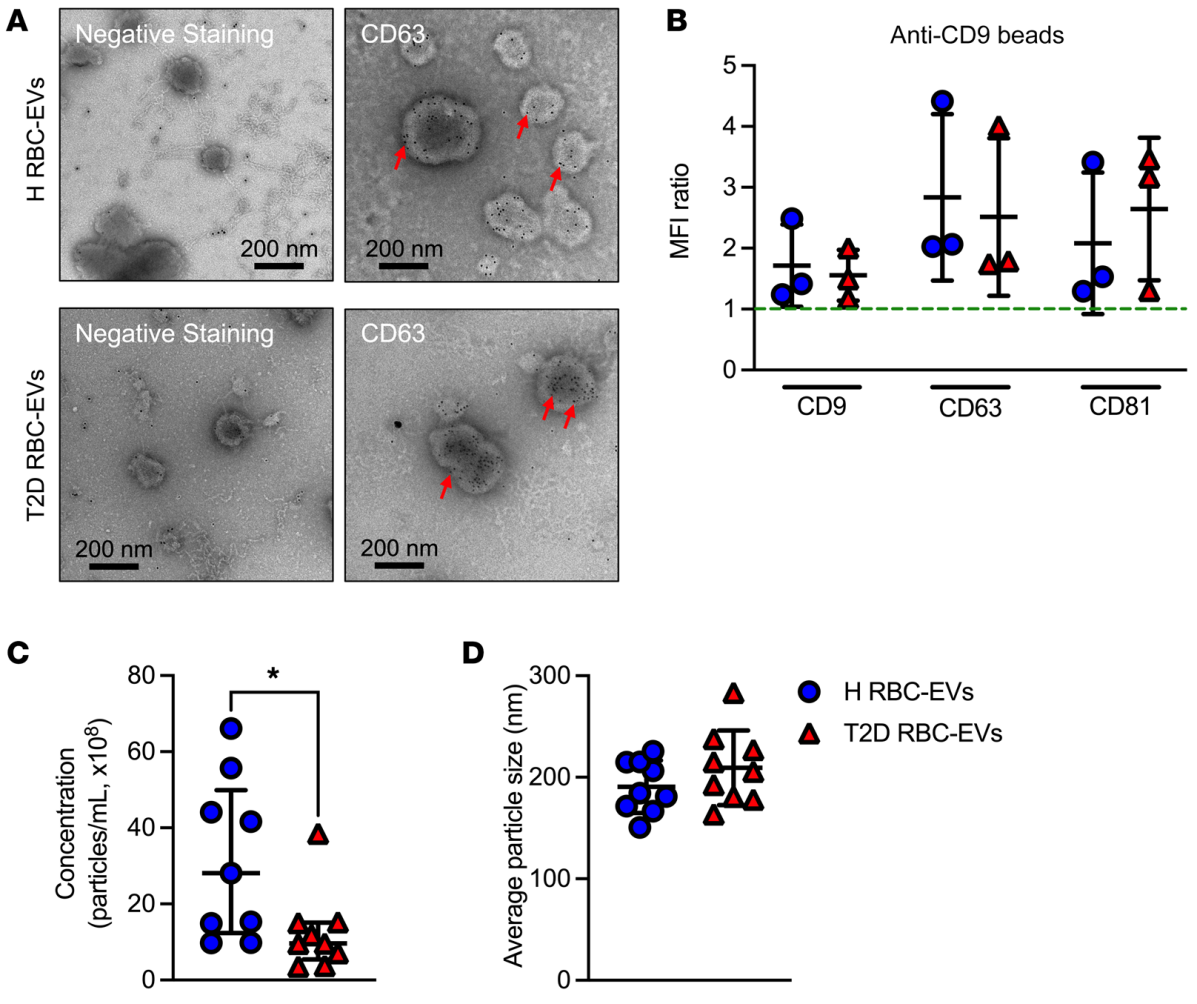
EVs are released by human RBCs. Representative TEM images of EVs derived from RBCs from healthy subjects (H RBC-EVs) and patients with T2D (T2D RBC-EVs) negatively stained or immunostained with gold-labeled anti-CD63 antibody. Red arrows point at positive signals (black dots) for CD63-gold beads (**A**, *n* = 3). EVs were captured by anti-CD9–coated latex beads, detected either by anti-CD9, anti-CD63, or anti-CD81. The MFI was measured by flow cytometry. The dotted line indicates a signal above 1, which was considered as a positive signal (**B**, *n* = 3). Quantification of EV concentration (**C**, *n* = 9). Distribution of the average particle size of H RBC-EVs and T2D RBC-EVs (**D**, *n* = 9). Values are expressed as mean ± SD (**B** and **D**) and median ± interquartile range (Q1–Q3) (**C**). *P < 0.05, Mann-Whitney *U* test.

RBCs from healthy subjects (H-RBCs) and T2D-RBCs were cultured in serum-free media, and EVs were isolated and then visualized by transmission electron microscopy (TEM) with negative staining or immunostaining with gold-labeled anti-CD63 antibody. Representative TEM images of negative staining or CD63 gold-labeled EVs derived from H-RBCs (H RBC-EVs) and T2D-RBCs (T2D RBC-EVs) are shown in Figure 1A. Most of the EVs appeared intact, and the TEM pictures show the presence of spherical, membrane-capsuled structures ranging between 50 and 300 nm in diameter (Figure 1A).

EVs were characterized by bead-based flow cytometry. Purified EVs were bound to anti-CD9–coated latex beads. This confirmed the presence of CD9, CD63, and CD81, 3 tetraspanins that are enriched on EVs, on EVs derived from H-RBCs and T2D-RBCs (Figure 1B).

Nanoparticle-tracking analysis (NTA) revealed that the concentration of T2D RBC-EVs was significantly lower than that of EVs from H-RBCs (Figure 1C). The average size of T2D RBC-EVs and H RBC-EVs did not differ (Figure 1D). H RBC-EVs and T2D RBC-EVs did not affect human carotid artery endothelial cell (HCtAEC) viability (Supplemental Figure 1; supplemental material available online with this article; https://doi.org/10.1172/JCI180900DS1).

### Increased uptake of T2D RBC-EVs by endothelial cells.

**Figure 2:**
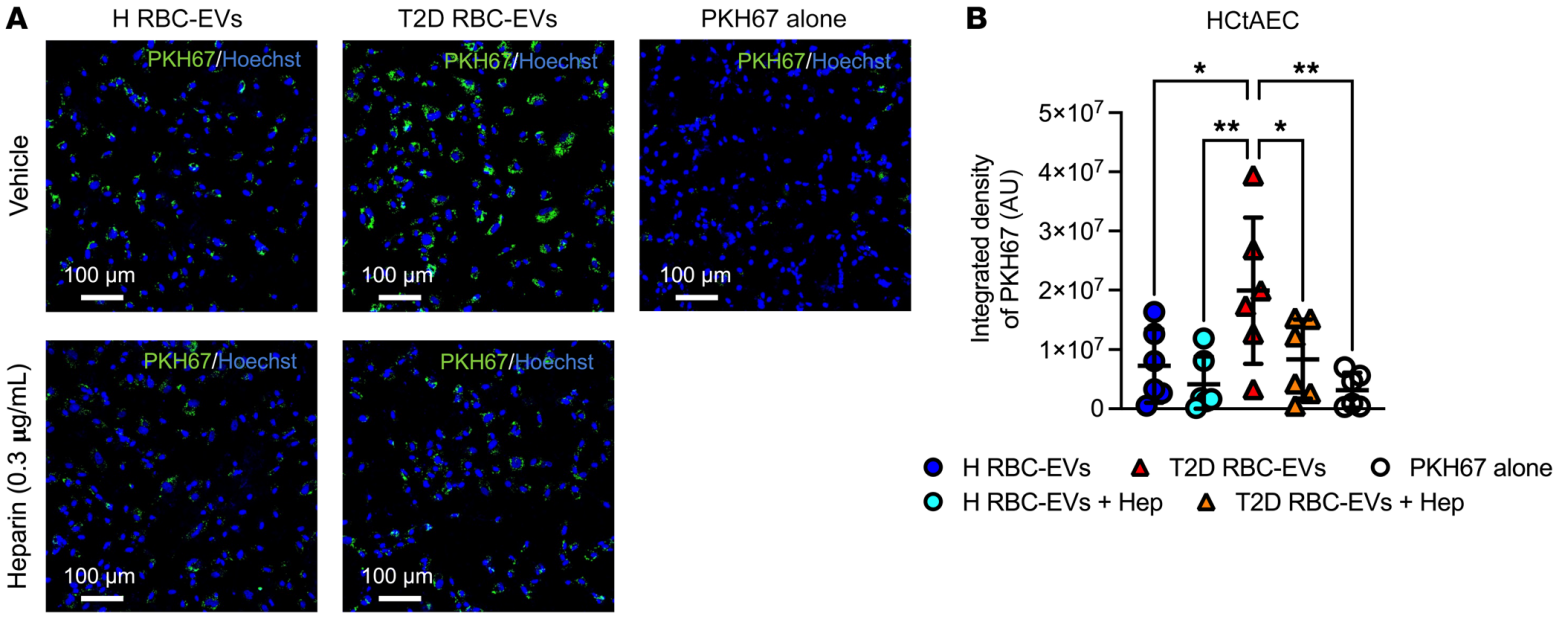
Increased uptake of the RBC-derived EVs from T2D patients by endothelial cells. Representative immunofluorescence images depicting PKH67 (green) in the absence or presence of heparin (Hep) in HCtAECs (**A**, *n* = 6). Quantitative analyses of the integrated density for PKH67-labeled EVs after 24 hours of coincubation with HCtAECs in the absence or presence of heparin (**B**, *n* = 6). Values are expressed as mean ± SD. *P < 0.05; **P < 0.01, 1-way ANOVA.

To study the communication of RBC-derived EVs with endothelial cells, we determined the uptake of EVs by endothelial cells using PKH67-stained EVs. Despite the lower number of EVs produced by T2D-RBCs described above, the uptake of T2D RBC-EVs by HCtAECs was significantly greater than that of H RBC-EVs (Figure 2, A and B, and Supplemental Figure 2A). It has been described that such incorporation occurs through forming multivesicular endosomes (11), and we observed that T2D RBC-EVs were colocalized with the intercellular endosome marker RAB5A in HCtAEC (Supplemental Figure 2B).

Heparin is known to inhibit the uptake of EVs by recipient cells (12, 13). Administration of heparin during coincubation indeed inhibited the uptake of T2D RBC-EVs by endothelial cells (Figure 2, A and B, and Supplemental Figure 2A).

### EVs derived from T2D-RBCs induce endothelial dysfunction.

**Figure 3:**
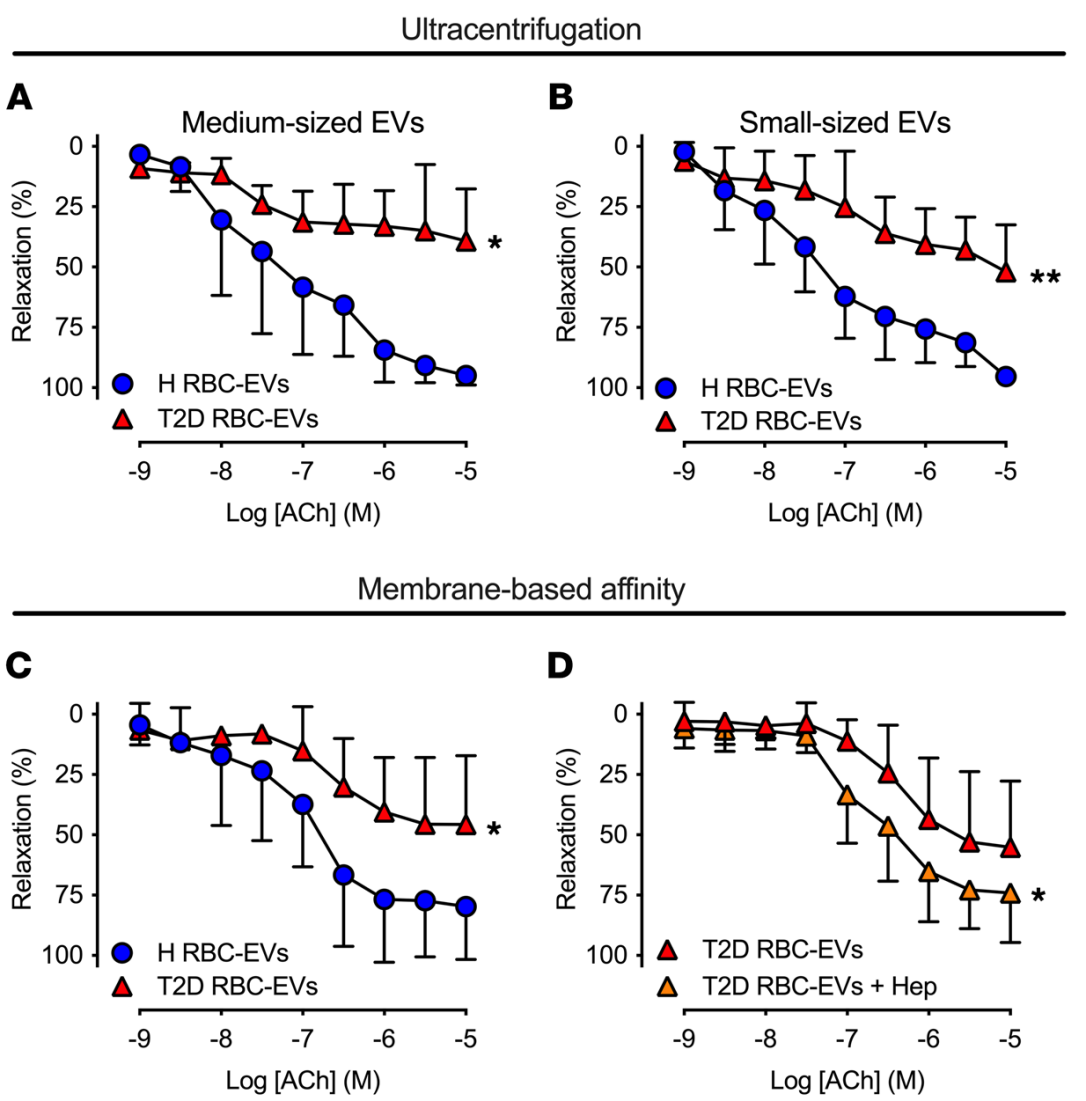
T2D RBC-EVs induce endothelial dysfunction, rescued by uptake inhibition. EDR evoked by ACh in mouse aortas following 18 hours of coincubation with H RBC-EVs and T2D RBC-EVs. EVs were isolated by sequential ultracentrifugation (**A**, *n* = 4 and **B**, *n* = 3–9) or membrane affinity columns (**C**, *n* = 6–9). EDR evoked by ACh in mouse aortas following 18 hours of coincubation with T2D RBC-EVs and heparin (**D**, *n* = 7). Values are expressed as mean and SD. *P < 0.05; **P < 0.01, repeated-measures 2-way ANOVA.

Having established that endothelial cells take up T2D RBC-EVs to a greater extent, we next investigated the functional impact of these EVs on endothelial function. Following coincubation of mouse aortic rings with H RBC-EVs and T2D RBC-EVs, both medium- and small-sized EVs isolated from T2D-RBCs by differential ultracentrifugation impaired endothelium-dependent relaxation (EDR) when compared with H RBC-EVs (Figure 3, A and B). Similarly, RBC-derived EVs isolated by membrane affinity also significantly impaired EDR in T2D (Figure 3C). By contrast, we did not observe any detrimental effect of EVs isolated by the 2 different methods on endothelium-independent relaxation (EIR) (Supplemental Figure 3, A–C), confirming that the EVs derived from T2D-RBCs selectively impaired endothelial function, independently of which isolation method was used. Consequently, the subsequent studies were performed using EVs isolated by membrane affinity.

As the concentration of the EVs derived from H-RBCs was higher than that from RBCs of T2D patients, we performed additional functional studies using the same amount of EVs for both groups. Also in this situation, T2D RBC-EVs impaired endothelial function (Supplemental Figure 4A), indicating that RBC-derived EVs induced endothelial dysfunction in T2D irrespective of whether the unadjusted concentration or the same amount of EVs were administered.

To elucidate the functional implications of the greater uptake of T2D RBC-EVs by endothelial cells, we applied heparin during the 18-hour coincubation of EVs with mouse aortic rings. Following coincubation, heparin significantly attenuated the impairment of EDR induced by T2D RBC-EVs in comparison with vessels incubated without heparin (Figure 3D), while heparin did not affect EDR in vessels incubated with H RBC-EVs (Supplemental Figure 4B). These results clearly indicate that T2D RBC-EVs enter the endothelial cells to induce endothelial dysfunction.

### EVs derived from T2D-RBCs carrying arginase-1 induce endothelial dysfunction.

**Figure 4:**
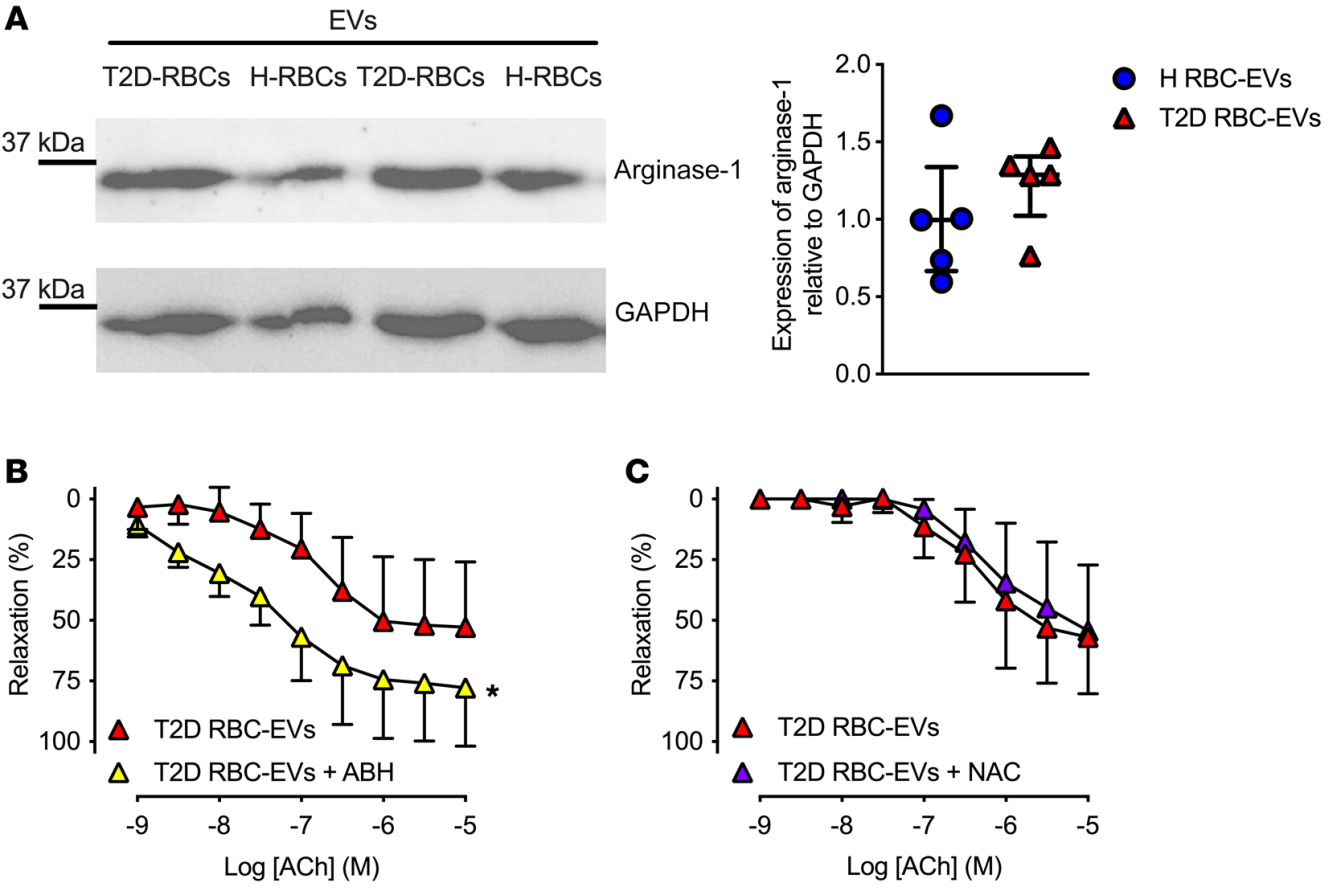
Arginase-1 is present in RBC-derived EVs and mediates endothelial dysfunction. Western blot images of arginase-1 (35 kDa) and GAPDH (36 kDa) in isolated EVs derived from H-RBCs and T2D-RBCs and quantification of the expression normalized to GAPDH (**A**, *n* = 5). EDR evoked by ACh in mouse aortas following 18 hours of coincubation of T2D RBC-EVs with the arginase inhibitor ABH (**B**, *n* = 6). EDR evoked by ACh in mouse aortas following 18 hours of coincubation of T2D RBC-EVs with the antioxidant NAC (**C**, *n* = 5). Values are expressed as median ± interquartile range (Q1–Q3) (**A**) and mean and SD (**B** and **C**). *P < 0.05, repeated measures 2-way ANOVA (**B**).

Since our group previously discovered that T2D-RBCs induce endothelial dysfunction via upregulation of arginase-1 and increased formation of ROS, we hypothesized that arginase-1 and ROS are key players in the endothelial dysfunction triggered by T2D RBC-EVs. Western blot analysis showed that arginase-1 was present in EVs and carried by H RBC-EVs and T2D RBC-EVs. The expression of arginase-1 did not differ significantly between T2D RBC-EVs and H RBC-EVs (Figure 4A). To further elucidate the role of arginase-1 carried by the EVs in the development of endothelial dysfunction, we coincubated mouse aortic rings with T2D RBC-EVs in the presence or absence of the arginase inhibitor 2(S)-amino-6-boronohexanoic acid (ABH). Notably, ABH significantly attenuated the impairment in endothelial function induced by EVs derived from T2D-RBCs (Figure 4B). In contrast, the addition of the antioxidant N-acetylcysteine (NAC) to the coincubation of EVs and aortic rings did not affect EDR (Figure 4C). To ensure that the inhibitory effect of ABH during the 18-hour coincubation with T2D RBC-EVs did not carry over to affect endothelial function in the myograph studies, control experiments were conducted using aortas from T2D mice (*db/db*), which exhibit severe endothelial dysfunction (Supplemental Figure 5). Incubation with ABH for 18 hours followed by washing did not improve endothelial function. However, when vessels were incubated with ABH for 18 hours and again given ABH in the organ bath for 1 hour, the endothelial function was improved. This indicates that the beneficial effect of arginase inhibition during the coincubation of EVs and the aortas was achieved by inhibiting arginase selectively in the EVs (Figure 4B). Taken together, these observations indicate that T2D RBC-EVs impaired endothelial function through arginase signaling carried by the EVs.

### EVs derived from T2D-RBCs induce endothelial dysfunction through upregulated arginase-1 and increased oxidative stress in vasculature.

**Figure 5:**
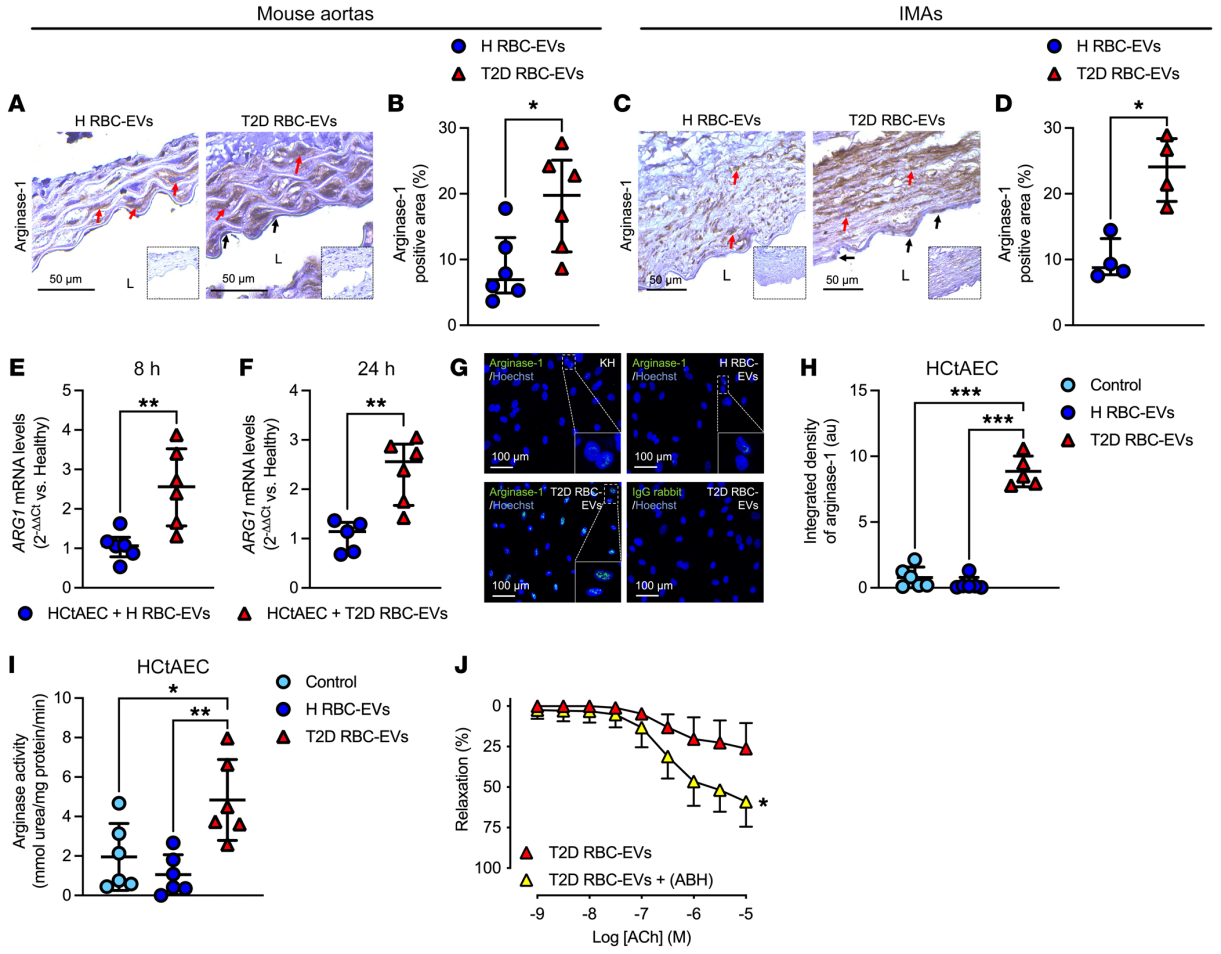
T2D RBC-EVs induce endothelial dysfunction through vascular arginase-1. Immunohistochemical images (**A**, *n* = 6) and quantification (**B**, *n* = 6) of arginase-1 in aortic rings and in human IMAs (**C** and **D**, *n* = 4) following 18 hours of coincubation with H RBC-EVs and T2D RBC-EVs. Inserts show IgG controls. L indicates lumen, black arrows endothelial cells, and red arrows smooth muscle cells. mRNA levels of arginase-1 (*ARG1*) in HCtAEC after coincubation with H RBC-EVs and T2D RBC-EVs for 8 hours (**E**, *n* = 6) and 24 hours (**F**, *n* = 5–6). Immunofluorescence (**G**, *n* = 5–6) and quantification (**H**, *n* = 5–6) of arginase-1 (green) in HCtAECs following 24 hours of coincubation with H RBC-EVs and T2D RBC-EVs. Arginase activity in HCtAECs incubated with medium (control), H RBC-EVs, and T2D RBC-EVs for 24 hours (**I**, *n* = 6). EDR evoked by ACh in mouse aortas following 18 hours of coincubation with T2D RBC-EVs with and without the administration of ABH to the vessels for 1 hour in the organ baths (**J**, *n* = 5). Parentheses indicate that the inhibitor was added in the organ baths for 1 hour following the 18 hours of coincubation with T2D RBC-EVs. Values are expressed as median ± interquartile range (Q1–Q3) (**B** and **D**–**F**), mean ± SD (**H** and **I**), and mean and SD (**J**). *P < 0.05; **P < 0.01; ***P < 0.001, Mann-Whitney *U* test (**B** and **D**–**F**), 1-way ANOVA (**H** and **I**), and repeated measures 2-way ANOVA (**J**).

It is known that arginase in RBCs regulates endothelial dysfunction via upregulated arginase and reduced NO production in endothelial cells, which has been implicated in other pathologies such as T2D, familial hypercholesterolemia, and COVID-19 (4, 14, 15). Based on this, we hypothesized that vascular arginase and ROS production contribute to endothelial dysfunction induced by EVs derived from T2D-RBCs. Expression of arginase-1 was significantly increased in mouse aortas following coincubation with T2D RBC-EVs compared with aortas incubated with H RBC-EVs (Figure 5, A and B). In contrast, no change was found in arginase-2 expression (Supplemental Figure 6, A and B). T2D RBC-EVs also increased arginase-1 expression levels in human internal mammary arteries (IMAs) (Figure 5, C and D). Coincubation of HCtAEC with EVs derived from T2D-RBCs increased arginase-1 (*ARG1*) mRNA levels at 8 hours and 24 hours (Figure 5, E and F) and protein levels (Figure 5, G and H, and Supplemental Figure 7A), whereas no change was observed in arginase-2 (*ARG2*) mRNA levels (Supplemental Figure 6, C and D). The increase in arginase-1 protein was translated into increased arginase activity in HCtAECs following incubation with T2D RBC-EVs compared with medium or H RBC-EVs (Figure 5I). Further, pharmacological inhibition of vascular arginase, using the arginase inhibitor ABH significantly attenuated the impairment in endothelial function induced by T2D RBC-EVs (Figure 5J).

**Figure 6:**
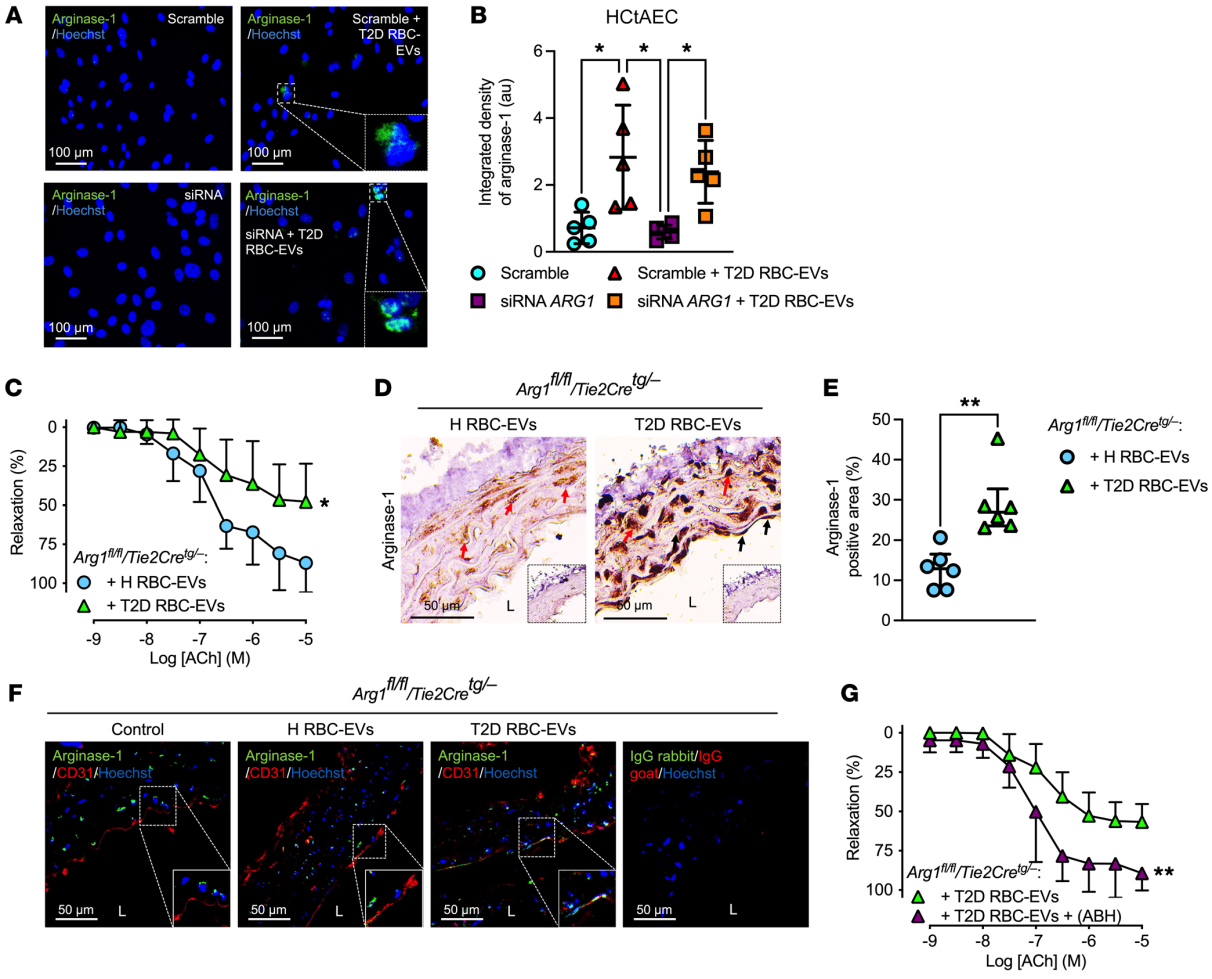
T2D RBC-EVs induce endothelial dysfunction through the transfer of arginase-1 to the endothelium. Immunofluorescence (**A**, *n* = 5) and quantification (**B**, *n* = 5) of arginase-1 (green) in HCtAECs transfected with scramble or siRNA for arginase-1 (*ARG1*) and coincubated with T2D RBC-EVs for 24 hours. EDR evoked by ACh in mouse aortic rings from *Arg1^fl/fl^/Tie2Cre^tg/–^* mice following 18 hours of coincubation with H RBC-EVs and T2D RBC-EVs (**C**, *n* = 6–7). Immunohistochemical images (**D**, *n* = 6) and quantification (**E**, *n* = 6) of arginase-1 in aortas from *Arg1^fl/fl^/Tie2Cre^tg/–^* mice following 18 hours of coincubation with H RBC-EVs and T2D RBC-EVs. Inserts show IgG controls. L indicates lumen, black arrows endothelial cells, and red arrows smooth muscle cells. Immunofluorescence images of arginase-1 (green) and CD31 (red) in aortas from *Arg1^fl/fl^/Tie2Cre^tg/–^* mice following coincubation with H RBC-EVs and T2D RBC-EVs. Nuclei were stained with Hoechst (blue) (**F**, *n* = 5). EDR evoked by ACh in mouse aortas from *Arg1^fl/fl^/Tie2Cre^tg/–^* mice following 18 hours of coincubation with T2D RBC-EVs with and without the administration of ABH to the vessels for 1 hour in the organ baths (**G**, *n* = 6). Parentheses indicate that the inhibitor was added in the organ baths for 1 hour following the 18 hours of coincubation with T2D RBC-EVs. Values are expressed as mean ± SD (**B**), mean and SD (**C** and **G**), and median ± interquartile range (Q1–Q3) (**E**). *P < 0.05; **P < 0.01, 1-way ANOVA (**B**), repeated measures 2-way ANOVA (**C** and **G**), and Mann-Whitney U test (**E**).

To better understand the mechanism behind the increased arginase-1 levels in endothelial cells induced by T2D RBC-EVs, we silenced *ARG1* mRNA in HCtAECs. Quantitative PCR (qPCR) analysis showed that *ARG1* mRNA levels in HCtAECs were decreased by siRNA (Supplemental Figure 7B). Interestingly, a significant increase in protein levels of arginase-1 was still apparent after coincubation of siRNA-transfected cells with T2D RBC-EVs (Figure 6, A and B, and Supplemental Figure 7C), suggesting that the EVs transferred arginase-1 protein to the endothelial cells. To shed additional light on the possibility of transfer of arginase-1 protein, we performed experiments using aortas from endothelial cell arginase-1–KO mice (*Arg1^fl/fl^/Tie2Cre^tg/–^*). Notably, these vessels also developed endothelial dysfunction following incubation with T2D RBC-EVs compared with those incubated with H RBC-EVs (Figure 6C). No differences in endothelial function were observed between *Arg1^fl/fl^/Tie2Cre^tg/–^* and their littermates (*Arg1^fl/fl^/Tie2Cre^–/–^*) when they were incubated with H RBC-EVs (Supplemental Figure 8A) or with T2D RBC-EVs (Supplemental Figure 8B). RBC-derived EVs did not affect EIR in aortas from *Arg1^fl/fl^/Tie2Cre^tg/–^* or *Arg1^fl/fl^/Tie2Cre^–/–^* mice (Supplemental Figure 8, C and D). Interestingly, incubation of aortas from endothelial cell arginase-1–KO mice with T2D RBC-EVs led to increased expression of arginase-1 in the vessel wall, including endothelial cells (Figure 6, D and E), similar to what was observed in WT mouse aortas incubated with T2D RBC-EVs (Figure 5, A and B). Arginase-1 was coexpressed with CD31 in the aortas of *Arg1^fl/fl^/Tie2Cre^tg/–^* mice following incubation with T2D RBC-EVs (Figure 6F), suggesting colocalization in the endothelium. Moreover, the endothelial dysfunction induced by T2D RBC-EVs in aortas from endothelial cell arginase-1–KO mice was attenuated by the arginase inhibitor ABH (Figure 6G). These results provide further support for the conclusion that EVs derived from T2D-RBCs transfer arginase-1 protein to endothelial cells, leading to endothelial dysfunction.

**Figure 7:**
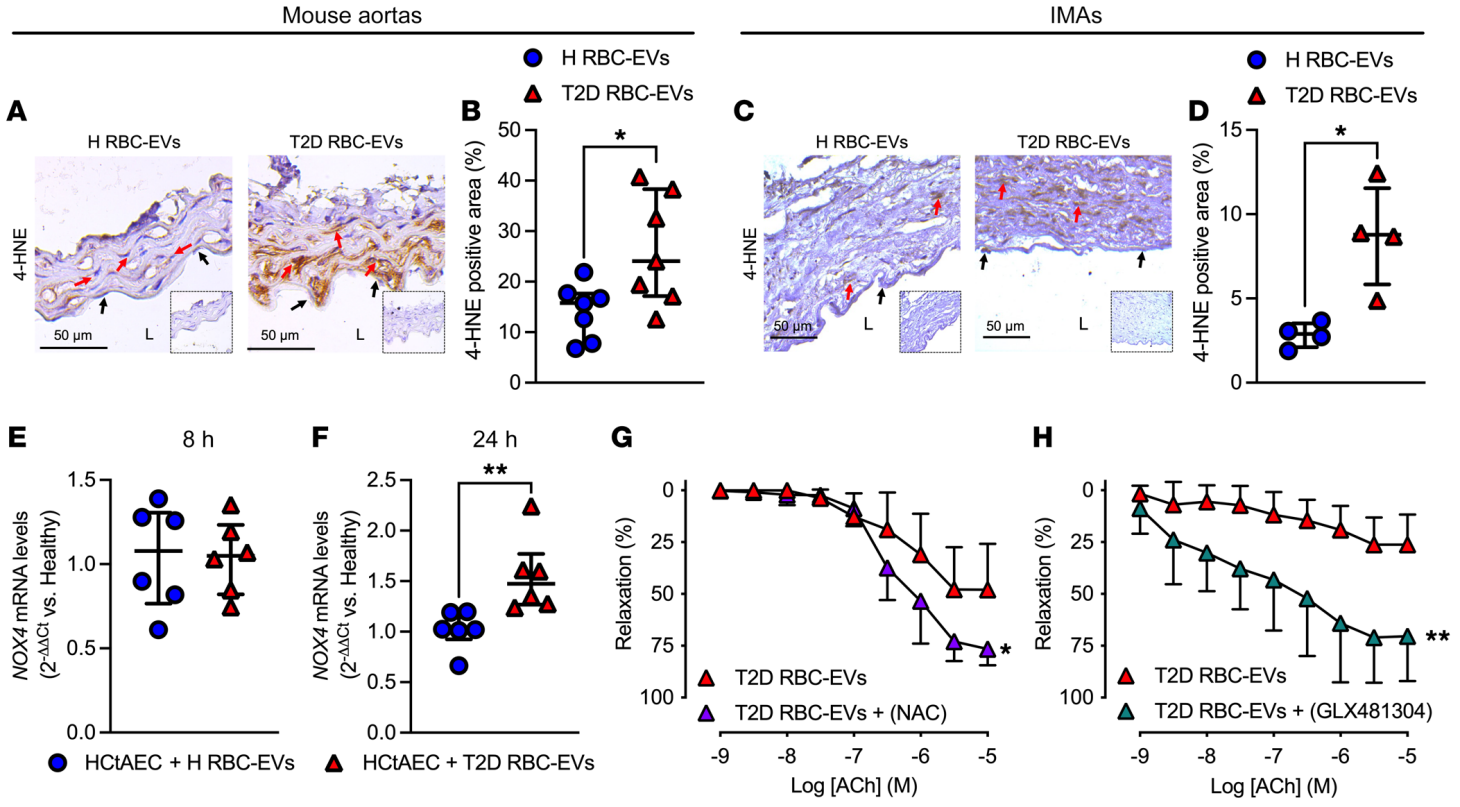
T2D RBC-EVs induce endothelial dysfunction through ROS. Immunohistochemical images (**A**, *n* = 7) and quantification (**B**, *n* = 7) of 4-HNE in mouse aortic rings following 18 hours of coincubation with H RBC-EVs and T2D RBC-EVs. Immunohistochemical images (**C**, *n* = 4) and quantification (**D**, *n* = 4) of 4-HNE in IMAs following 18 hours of coincubation with H RBC-EVs and T2D RBC-EVs. Inserts show IgG controls. L indicates lumen, black arrows endothelial cells, and red arrows smooth muscle cells. mRNA levels of *NOX4* after coincubation of HCtAECs with H RBC-EVs and T2D RBC-EVs for 8 hours (**E**, *n* = 6) and 24 hours (**F**, *n* = 6). EDR evoked by ACh in mouse aortas following 18 hours of coincubation with T2D RBC-EVs with and without administration of NAC to the vessels for 1 hour in the organ baths (**G**, *n* = 6). EDR evoked by ACh in mouse aortas following 18 hours of coincubation with T2D RBC-EVs with and without administration of GLX481304 (NOX2/4 inhibitor) to the vessels for 1 hour in the organ baths (**H**, *n* = 6). Parentheses indicate that the inhibitor was added in the organ baths for 1 hour following the 18 hours of coincubation with T2D RBC-EVs. Values are expressed as median ± interquartile range (Q1–Q3) (**B** and **D**–**F**), and mean and SD (**G** and **H**). *P < 0.05; **P < 0.01 using Mann-Whitney *U* test (**B**, **D**, and **F**), and repeated measures 2-way ANOVA (**G** and **H**).

Previous studies have demonstrated that increased oxidative stress in endothelial cells is linked to arginase (4, 16). To determine that EV-derived arginase also contributes to endothelial dysfunction by increasing oxidative stress in the vessel, we performed immunostaining on vessels incubated with T2D RBC-EVs and T2D RBC-EVs treated with ABH for 18 hours. We observed a significant reduction in the expression levels of the oxidative stress marker 4-hydroxynonenal (4-HNE) in vessels where arginase activity was inhibited (Supplemental Figure 9), suggesting that arginase-1 from T2D RBC-EVs contributes to increased oxidative stress in the endothelium. To explore the potential molecular changes induced by T2D RBC-EVs in the endothelium, we investigated oxidative stress triggered by T2D RBC-EVs. First, we conducted bulk RNA-Seq on HCtAECs after 24 hours incubation with H RBC-EVs and T2D RBC-EVs. Gene set enrichment analysis (GSEA) identified the upregulation of key pathways related to oxidative stress, such as the hallmark oxidative phosphorylation pathway and the hallmark ROS pathway (Supplemental Figure 10). In accordance with this finding, we next observed that levels of 4-HNE were increased in mouse aortas (Figure 7, A and B) and human IMAs incubated with T2D RBC-EVs (Figure 7, C and D). Further, T2D RBC-EVs significantly increased mRNA levels of NADPH oxidase 4 (*NOX4*) in HCtAECs at 24 hours but not at 8 hours (Figure 7, E and F), whereas NADPH oxidase 1 (*NOX1*) expression was unchanged (Supplemental Figure 11, A and B). To elucidate the functional implications of this change, we inhibited oxidative stress in the aorta by applying NAC and a specific inhibitor of NOX2/NOX4 (GLX481304) to the aortic segments after the coincubation with T2D RBC-EVs for 1 hour. Both NAC and GLX481304 attenuated the impairment in endothelial function induced by T2D RBC-EVs (Figure 7, G and H). Collectively, these observations suggest that T2D RBC-EVs increase vascular arginase-1 and oxidative stress, leading to endothelial dysfunction.

### Unbiased proteomic analysis of RBC-derived EVs.

To increase our knowledge regarding the proteome of the EVs derived from RBCs, we conducted an unbiased proteomics analysis of RBC-derived EVs from age-matched healthy controls and individuals with T2D using nontargeted liquid chromatography–tandem mass spectrometry–based (LC-MS/MS-based) proteomics. This analysis led to the identification of 1,053 unique proteins, including arginase-1, expressed in at least 2/3 of the samples for each group in total. The full list of the identified proteins by the proteomic analysis is presented in Supplemental Tables 2–4. As depicted in the Venn diagram (Supplemental Figure 12), 920 of these identified proteins were common to both H RBC-EVs and T2D RBC-EVs, whereas a minority of proteins were detected in H RBC-EVs only (77 proteins) or in T2D RBC-EVs only (56 proteins). This implies that the protein cargo carried by RBC-derived EVs is very similar between healthy and T2D conditions, highlighting the importance of the uptake of the T2D RBC-EVs by the endothelium.

## Discussion

This study highlights a potential disease mechanism by which EVs released from RBCs contribute to cardiovascular complications by impairing endothelial function in T2D. Despite intensive research and recent treatment advances, the mechanisms driving cardiovascular complications in T2D remain largely unclear, and the increased cardiovascular mortality among patients with T2D remains greatly elevated (17). In the current study, we provide evidence for a concept by which vascular injury in T2D occurs via release of EVs and transfer of signaling molecules from RBCs to endothelial cells. We demonstrate that the EVs secreted by T2D-RBCs are taken up by endothelial cells to a greater extent in comparison with H RBC-EVs and induce endothelial dysfunction via the transfer of arginase-1 by the EVs and increased vascular oxidative stress. Taken together, these findings identify the release and transfer of arginase-containing EVs as an important communication mechanism between RBCs and the vascular wall, resulting in endothelial dysfunction in T2D.

A wide range of evidence suggests that RBCs are active regulators of vascular homeostasis by playing a central role in the development of cardiovascular injury in different pathologies and are not just simple carriers of oxygen (4, 14, 15, 18). RBCs derived from T2D patients induce endothelial dysfunction via upregulation of arginase-1 and oxidative stress in the target vessel (4). However, the mechanism by which this endothelial dysfunction is mediated from the RBCs to the endothelium is poorly understood. It has remained unknown how the RBCs communicate with the vessel and how the transfer of signaling between the RBCs and the endothelial cells occurs. Since a large amount of EVs are produced by RBCs (10, 19), we hypothesized that EVs play a central role in this communication. The current study demonstrates the importance of the EVs released from T2D-RBCs as key mediators in the development of endothelial dysfunction induced by the RBCs. This effect was mediated both by medium-sized and small-sized EVs separated by sequential ultracentrifugation and was reproduced by using a membrane-based affinity method. Also, the specificity of EVs in the development of endothelial dysfunction was demonstrated by the inhibitory effect of heparin, which is known to interfere with the uptake of EVs by the recipient cells (12, 13).

The mechanism behind the endothelial dysfunction induced by T2D RBC-EVs was investigated in detail. Our group has shown that upregulated arginase-1 in RBCs is a key factor contributing to the development of endothelial dysfunction in T2D (4). We, therefore, hypothesized that the EVs derived from T2D-RBCs carry arginase-1, leading to endothelial dysfunction. Indeed, we demonstrate that the EVs contain arginase-1, and pharmacological inhibition of arginase prevented the development of endothelial dysfunction. In addition, the finding that incubation with the EVs from T2D-RBCs was associated with increased expression of arginase-1 in the vessel wall of both mouse aorta and human IMA suggests that the EVs induced endothelial dysfunction via delivery or upregulation of arginase-1 in the endothelium. A previous study demonstrated that arginase-containing circulating EVs from rodents and patients with T2D induced endothelial dysfunction (13), in line with the data from the present study. However, that study did not determine the origin of the EVs and speculated that they derived from the liver. Our data demonstrate that arginase-containing EVs are derived from RBCs to induce endothelial dysfunction in T2D. Deeper analyses showed that arginase-1 protein in endothelial cells was still increased after RNA silencing as well as in aortas from endothelial cell arginase-1–KO mice, suggesting that the increased levels of arginase in the endothelium after coincubation with T2D RBC-EVs is due to delivery of arginase-1 protein by the EVs derived from T2D-RBCs. The conclusion that the transfer of arginase-1 protein by the EVs to the endothelial cells is of functional importance is supported by the finding that T2D RBC-EVs induced endothelial dysfunction in aortas from endothelial cell arginase-1–KO mice. However, based on the finding that not only arginase protein but also mRNA was increased in endothelial cells, it cannot be excluded that de novo synthesis of arginase-1 also occurs. Collectively, these results provide important information regarding how RBCs communicate with and transfer signaling to the vascular endothelium to induce endothelial dysfunction in T2D.

Our previous study has revealed that endothelial cell oxidative stress induced by RBCs is partly driven by arginase-1 (4). We therefore explored the development of oxidative stress in the endothelium following incubation with EVs from T2D-RBCs. Accordingly, 4-HNE, a marker of oxidative stress, was increased in vessels incubated with T2D RBC-EVs, and this effect was attenuated by arginase inhibition. The transcriptomic analysis in endothelial cells after the incubation with T2D RBC-EVs supports the activation of pathways involved in the generation of oxidative stress. When HCtAECs were incubated with T2D RBC-EVs, we detected increased levels of *NOX4*, which is a key enzyme for superoxide production in diabetes (20). The attenuation of vascular oxidative stress by NAC and the NOX2/NOX4 inhibitor prevented the impairment of EDR induced by T2D RBC-EVs, suggesting a functional role of NOX4-derived ROS in the development of endothelial dysfunction in T2D. In a previous study, it was demonstrated that vascular NOX1 was of importance for RBC-mediated endothelial dysfunction in T2D (4). This may suggest that different NOX isoforms may be involved in endothelial dysfunction mediated by RBCs and RBC-derived EVs.

In the unbiased proteomic analysis, several proteins in addition to arginase-1 were identified in EVs from RBCs of both patients with T2D and healthy controls. Some of these proteins are of potential interest because of their involvement in oxidative stress generation, cell adhesion, and inflammation, which are processes of relevance to the pathophysiology of T2D. Further investigations are needed to fully understand the function of several of these other proteins present in RBC-derived EVs.

A highly interesting observation is that EVs derived from T2D-RBCs are taken up by endothelial cells in a larger number than EVs from H-RBCs. This increased uptake of EVs in endothelial cells was observed despite the lower number of EVs released from T2D-RBCs. Although, it is well-known that EVs are key players in cell-to-cell communication (9), the pathways involved in the uptake and trafficking of EVs between different cell types are poorly understood. The observation that inhibition of EV uptake by heparin prevented the development of endothelial dysfunction indicates that the increased uptake of RBC-derived EVs by the endothelial cells is an important feature of the endothelial dysfunction induced by these EVs. The increased uptake of EVs by endothelial cells therefore seems to be an important determinant of the endothelial dysfunction induced by the EVs. The inhibition of EV uptake by heparin suggests involvement of heparan sulphate proteoglycans (12, 21). Interestingly, our proteomic analysis of EVs revealed the presence of syndecan-4, which is a major protein component of heparan sulphate proteoglycans expressed on cell membranes (21). This may suggest that the heparan sulphate glycoproteins on RBC-derived EVs are involved in the regulation of their uptake. Another protein detected in the EVs is CD44, which is a cell-surface glycoprotein that binds to the components of the glycocalyx, such as hyaluronic acid, and is involved in cell adhesion and migration (21, 22). The precise involvement of these and other mechanisms behind the increased uptake of EVs by endothelial cells certainly warrants further studies.

The present study has certain limitations. The patients included had several comorbidities, including coronary artery disease, peripheral artery disease, and nephropathy, and were treated with antidiabetic and preventive cardiovascular medications that may have affected EV uptake or the effect of EVs on endothelial function. However, results from a previous study (4) suggest that neither comorbidities nor comedications affect RBC-induced endothelial function. Further, since many of the medications (statins, angiotensin-converting enzyme inhibitors and receptor antagonists, and glucose-lowering medication) are known to improve endothelial function (23), they would be expected to result in an underestimation of the endothelial dysfunction observed following incubation with RBC-derived EVs from patients taking these medications. It should also be noted that these observations are confined to ex vivo incubations and determination of endothelial dysfunction. It can, therefore, not be determined to which degree RBC-derived EVs contribute to endothelial dysfunction in vivo. The advantage of the present study design is that we can specifically determine the effect of the EVs derived from RBCs, which is challenging in the in vivo situation. Additionally, it remains unknown whether certain characteristics of RBCs, such as fragility and deformability, influence EV production and function, and this deserves to be investigated in future studies.

In conclusion, the present study demonstrates a mechanism behind vascular injury in T2D mediated by RBC-derived EVs and provides important advances in understanding vascular complications in T2D. The study demonstrates that EVs produced by RBCs from patients with T2D induce endothelial dysfunction through a mechanism involving increased uptake of EVs in endothelial cells, delivery of arginase-1, and induction of vascular oxidative stress. The study demonstrates the mechanism of signal transfer from RBCs to the vascular endothelium to induce endothelial dysfunction in T2D. These results shed light on the mechanism underlying vascular injury and thereby provide the basis for identifying targets for the treatment of vascular complications in T2D. Therapeutic strategies that interfere with the uptake of RBC-derived EVs by endothelial cells, the cargo of the EVs, or the transfer of signaling molecules by RBC-derived EVs may have the potential to prevent vascular injury in T2D.

## Methods

### Sex as a biological variable.

Sex was not considered a biological variable in this study. Our study examined male and female humans and animals, without making any distinction between sexes.

### Patient recruitment.

Patients with T2D (*n* = 68), defined according to the WHO criteria, were recruited from the Department of Endocrinology and Diabetology, Karolinska University Hospital and Center for Diabetes, Academic Specialist Center, Health Care Services Stockholm County, Sweden, for collection of blood samples and isolation of RBCs. A group of age-matched healthy control subjects (*n* = 35) was recruited from the Department of Cardiology, Karolinska University Hospital. Another group of nondiabetic subjects (*n* = 4) scheduled for coronary artery bypass surgery was contacted for collection of IMAs. The characteristics of those subjects are summarized in Supplemental Table 1.

### Blood sample preparations.

Fresh blood samples were collected in EDTA tubes (BD Vacutainer blood collection tubes; BD Biosciences) by puncture of the cubital vein after a fasting period of at least 12 hours. Routine blood tests were sent to clinical chemistry. RBCs were isolated following several centrifugation steps. Whole blood was centrifuged at 1,000*g* at 4°C for 10 minutes, after which plasma, buffy coat, and the top part of the RBC layer were removed. Subsequently, 3 cycles of washing (with centrifugation at 1,000*g* at 4°C for 5 minutes each cycle) with Krebs-Henseleit (KH) buffer (pH 7.4) containing 118 mM NaCl, 4.7 mM KCl, 1.2 mM MgSO_4_, 1.2 mM KH_2_PO_2_, 25 mM NaHCO_3_, 11 mM glucose, and 2.4 mM CaCl_2_ were conducted to obtain purified RBCs (14). This procedure has been shown to remove 99% of white blood cells and 98% of platelets (24).

### Extracellular vesicle isolation.

Freshly isolated RBCs were diluted to a hematocrit of 20% in KH buffer and incubated for 18 hours at 37°C and 5% CO_2_ for EV release. Afterward, the conditioned medium was collected and centrifuged twice, the first time at 300*g* at 4°C for 10 minutes, and the collected supernatant was centrifuged at 3,000*g* at 4°C for 30 minutes. The conditioned medium was then stored at –80ºC until further use.

For the isolation of EVs, 2 different isolation methods were used, ultracentrifugation and membrane-based affinity. For the ultracentrifugation, samples were prefiltered using 0.8 μm filters (Acrodisc 25 mm w/0.8 μm Supor; Pall Corporation), and the EVs were isolated by serial ultracentrifugation (yielding medium- and small-sized EVs). Briefly, medium-sized EVs were collected after centrifugation at 16,500*g* at 4°C for 30 minutes, after which the EVs were resuspended in KH buffer. The supernatant was filtered through a 0.22 μm filter and then ultracentrifuged twice at 100,000*g* at 4°C for 2 hours (NVT90 Rotor; Beckman Coulter) to obtain small-sized EVs (25). The small-sized EVs were resuspended in KH buffer. The EVs were also isolated using membrane affinity with the exoEasy Maxi Kit (QIAGEN) following the manufacturer’s instructions. EVs were concentrated with centrifugal filters (Amicon Ultra-4; Sigma-Aldrich), and a buffer exchange was done to KH buffer. Isolated EVs were stored at –80°C or used immediately.

### Characterization of EVs.

EV preparation for gold-immunostaining TEM was performed by the 3D-EM Core Facility at Karolinska Institutet. Mouse monoclonal anti-human CD63 (clone MEM-259, catalog no. ab8219; Abcam) and polyclonal goat IgG H&L (10 nm gold; catalog no. ab39619; Abcam) antibodies were used. Human FcR-Blocking Reagent (catalog no. 130-059-901; Miltenyi Biotec) was added to each EV sample, mixed, and allowed to incubate for at least 48 hours before the EV samples were prepared for imaging. For the experiment, formvar-carbon grids (200 mesh, copper; Electron Microscopy Sciences) were initially glow discharged for 2 minutes, 25 mA, and then EV samples were incubated on the grid for 1 minute. Grids were washed 4 times with phosphate-buffered saline (PBS) and blocked with 1% BSA/PBS for 1 minute; 3.5 μL of the primary antibody in 0.5% BSA/PBS was added for 1 minute, washed with 1.0% BSA, and then with PBS. Grids were incubated with the gold-labeled secondary antibody (polyclonal goat IgG; catalog no. ab39619; Abcam) for 5 minutes and rinsed with 1.0% BSA/PBS and Millipore water. Control grids incubated with only the EV and secondary antibodies were also used. The grids were stained with 1% uranyl acetate and then imaged using a Talos 120C G2 (Thermo Fisher Scientific) equipped with a Ceta-D detector at ×22,000 and ×45,000 magnification.

To measure EV concentration and size distribution, samples were diluted in PBS (30 kDa- filtered) and analyzed by NTA running the NTA 3.0 analytical software package (NanoSight). An LM10 platform with an sCMOS camera (NanoSight) equipped with a 405 nm laser was used. The diluted samples were analyzed with camera level 10 and detection threshold 3, and for each sample, 4 consecutive videos of 30 seconds each were recorded at room temperature while injecting the sample with a syringe pump (speed 50 mL/min).

EV characterization was done by bead-based flow cytometry. In brief, 4 μm diameter aldehyde/sulfate latex beads (Invitrogen) were coated with anti-human CD9 (clone HI9a; BioLegend) under agitation overnight at room temperature. Next, the antibody-coated beads were spun (10,000*g* at room temperature for 10 minutes) and blocked with 100 mM glycine for 30 minutes, followed by a wash with 1% BSA/PBS. The EV fractions from H-RBCs and T2D-RBCs were then bound to the anti-human CD9-coated beads, with a total of 1 μL beads per staining (1.3 × 10^5^ beads). The bead-EV complexes were washed (10,000*g* at room temperature for 10 minutes) and incubated for 30 minutes at 4°C with the following phycoerythrin-conjugated antibodies (2 μg/mL): anti-CD9 (clone HI9a), anti-CD63 (clone H5C6), anti-CD81 (clone 5A6), and their corresponding isotype control IgG_1_ (clone MOPC-21). All antibodies were purchased from BioLegend. The samples were then washed in PBS (2,500*g* at 4°C for 5 minutes). The bead-EV complexes were then analyzed using a FACSCanto II (BD Biosciences), and data were analyzed with FlowJo software version 10.9.0 (FlowJo). All surface markers were normalized using isotype controls.

### Tissue preparation and myograph studies.

Male and female WT C57BL/6 mice (Janvier Labs), a diabetic mouse model (*db/db* mice, Janvier Labs), mice lacking arginase-1 in endothelial cells and hematopoietic cells (*Arg1^fl/fl^/Tie2Cre^tg/–^*), and their littermates, *Arg1^fl/fl^/Tie2Cre^–/–^* (a gift from Eleonore Köhler, Maastricht University), were used at the age of 12–16 weeks. Previous studies have demonstrated that the *Arg1^fl/fl^/Tie2Cre^tg/–^* mice lack *ARG1* mRNA and protein in endothelial cells (26). It has previously been shown that mice carrying the fully functional floxed alleles of the arginase-1 gene (*Arg1^fl/fl^*) are indistinguishable from their WT littermates (27), and the littermates were therefore used as control animals. Mice were housed in the animal facility (Comparative Medicine) at Karolinska Institutet and kept in 12-hour light/12-hour dark cycles with free access to standard chow and water.

Mice were anesthetized with pentobarbital sodium (50 mg/kg i.p.), followed by thoracotomy and removal of the aorta. The aorta was cleaned by removing fat and connective tissues and subsequently cut transversely into 2 mm long aortic segments. The aortic rings were incubated with the same volume of isolated EVs for 18 hours at 37°C and 5% CO_2_. Afterward, vessel rings were mounted in a wire myograph (Danish Myo Technology A/S) in individual 6 mL organ baths containing KH buffer. Changes in isometric vascular tone were recorded with a Harvard Isometric Transducer (Harvard Apparatus). At the end of the equilibration period, the vessels were exposed to KCl (50 mM) twice to check the contractility. Thereafter, vessels were allowed to equilibrate in fresh KH buffer for 30 minutes before initiating different experimental protocols. EDR and EIR were evaluated by administration of cumulatively increasing concentrations of acetylcholine (ACh) (10^–9^–10^–5^ M) and sodium nitroprusside (SNP) (10^–9^–10^–5^ M), respectively, to vessels preconstricted by phenylephrine (10^–6^ M). In separate experiments, heparin (0.3 μg/mL), the arginase inhibitor ABH (10 mM), the antioxidant NAC (10 mM), or vehicle were administered during the 18-hour coincubation of EVs and aortic segments to selectively investigate the uptake of the EVs, function of EV arginase, and oxidative stress, respectively (4, 13). ABH (100 μM), NAC (10 μM), and a NOX2/4 inhibitor (GLX481304; 3 μM) were also applied for 1 hour into the organ baths after mounting the vessels on the wire myograph following the coincubation with EVs for 18 hours for determination of the involvement of vascular arginase and oxidative stress, respectively. All pharmacological compounds were purchased from Sigma-Aldrich.

### Histological and immunohistochemical analysis.

Mouse aortic rings or human IMAs were, following the EV coincubation for 18 hours, fixed for 24 hours in 4% formaldehyde (VWR International) at room temperature, dehydrated in graded ethanol (70, 95, and 99%), embedded in paraffin, sectioned using a microtome, and mounted on coated glass slides (Superfrost plus; Thermo Fisher Scientific). At least 6 slides containing approximately 4 tissue cross-sections (5 μm thick) from each animal were examined. Sections were deparaffinized in xylene and rehydrated in graded ethanol. For antigen retrieval, slides were subjected to high-pressure boiling in citrate buffer (pH 6.0). After peroxidase inactivation (0.3%) and blockade with goat serum (Abcam), aorta cross-sections were incubated overnight (4°C) with the following primary antibodies: rabbit polyclonal anti-human arginase-1 (1:100 dilution, catalog no. HPA003595; Atlas Prestige Antibody, Sigma-Aldrich), rabbit polyclonal anti-human arginase-2 (1:50 dilution, catalog no. HPA000663; Atlas Prestige Antibody, Sigma-Aldrich), and mouse monoclonal anti–4-HNE (1:200 dilution, IgG_2b_, clone 198960, catalog no. MAB3249; R&D Systems Inc.) (4, 14). Specific labeling was detected using labeled horseradish peroxidase (HRP) polymer conjugate as a secondary antibody as part of the EnVision^+^ FLEX Mini Kit (Dako, Agilent Technologies). Isotype controls were used as negative controls to confirm the specificity of the antibodies (rabbit IgG, catalog no. ab37415, and mouse IgG_2b_, clone MCP-11, catalog no. ab18469, both from Abcam). Samples were developed using a solution containing 3, 3′-diaminobenzidine (Dako, Agilent Technologies), then counterstained with Mayer’s modified hematoxylin (Abcam) and mounted using a mounting medium (Abcam). Fields from each aortic section were captured (Leica DM3000 Digital microscope; Leica Biosystems), digitized, and analyzed (ImageJ software, version 2.0, NIH).

For double immunofluorescence analysis, aorta cross-sections were permeabilized with 0.3% Triton X-100 for 10 minutes, blocked, and incubated overnight (4°C) with the following primary antibodies: rabbit polyclonal anti-human arginase-1 (1:100 dilution, catalog no. HPA003595; Atlas Prestige Antibody, Sigma-Aldrich) and goat polyclonal anti-mouse CD31 (1:25 dilution, catalog no. AF3628; R&D Systems Inc). Specific labeling was detected with an Alexa Fluor 488 chicken anti-rabbit secondary antibody (1:200 dilution, catalog no. A21441; Invitrogen, Life Technologies) and an Alexa Fluor 594 donkey anti-goat secondary antibody (1:200 dilution, catalog no. A11058; Invitrogen, Life Technologies), respectively. Cell nuclei were counterstained with Hoechst 33344 dye (1:1,000 dilution; Sigma-Aldrich) and mounted with SlowFade Gold antifade reagent (Molecular Probes). To confirm the specificity of antibodies, isotype controls were used as negative controls (rabbit IgG, catalog no. ab37415, or goat IgG, catalog no. 02-6202 from Abcam and Invitrogen, respectively). Fields were captured with the fluorescence microscope equipped with an ×40 objective lens and a ×10 eyepiece (Leica DM3000 digital microscope; Leica Biosystems), digitized, and analyzed (ImageJ software, version 2.0, NIH).

### Western blot.

EVs were lysed using RIPA lysis buffer (VWR International) containing protease inhibitors (Roche), sonicated 5 times at room temperature for 5 minutes each, and centrifuged at 16,000*g* and 4°C for 15 minutes. The total protein content of the extracts was quantified by a bicinchoninic acid protein assay kit (Pierce Biotechnology, Life Technologies). The proteins were separated on 10% SDS gel (5 μg per sample) and transferred onto 0.45 μm nitrocellulose blotting membranes (Amersham). Membranes were blocked with 5% milk for 1 hour at room temperature and incubated overnight at 4°C with a rabbit polyclonal anti-human arginase-1 primary antibody (1:1000 dilution, catalog no. HPA003595; Atlas Prestige Antibody, Sigma-Aldrich) and, after stripping of the membranes, with a rabbit polyclonal anti-human GAPDH (1:2,500 dilution; catalog no. G9545; Sigma-Aldrich). Membranes were subsequently washed, incubated for an additional hour with the secondary enzyme HRP-linked goat anti-rabbit polyclonal antibody (1:10000 dilution; catalog no. P0448; Dako, Agilent Technologies), and developed using the Amersham ECL Select Western Blotting Detection Reagent (Amersham). Signals were recorded using a chemiluminescent analyzer (ChemiDoc Imaging System; Bio-Rad) and analyzed with ImageJ software, version 2.0.

### LC-MS/MS analysis of EVs.

Proteomic characterization of EV samples was performed by the Proteomics Biomedicum Core Facility at Karolinska Institutet. Briefly, EV-enriched samples were dried using a vacuum concentrator (Eppendorf), solubilized in 25 μL of 8M urea in 50 mM Tris-HCl, pH 8.5, and sonicated in a water bath for 5 minutes. Samples were supplemented with 25 μL of 0.2% ProteaseMAX surfactant (Promega) in 50 mM Tris-HCl and 10% acetonitrile (ACN) and sonicated in a water bath for 5 minutes. Following the addition of 49 μL of Tris-HCl buffer, samples were probe sonicated using a Vibra-Cell probe (Sonics & Materials, Inc.) for 2 minutes with 2/2 seconds on/off pulse at 20% amplitude before a final sonication in a water bath for 5 minutes. The samples were centrifuged at 10,000*g* for 5 minutes, and the supernatants were transferred to new sample tubes.

Proteins were reduced by adding 1.5 μL of 500 mM dithiothreitol (Sigma-Aldrich) and incubated at 37°C for 45 minutes while shaking at 400 rpm on a block heater. Alkylation was performed with the addition of 3.2 μL of 500 mM iodoacetamide (Sigma-Aldrich) at room temperature for 30 minutes at 400 rpm in darkness. Then 0.5 μg of sequencing grade-modified trypsin (Promega) was added to the samples and incubated for 16 hours at 37°C. The digestion was stopped with 10 μL of formic acid (FA) (Sigma-Aldrich), incubating the solutions at room temperature for 5 minutes. The sample was cleaned on a HyperSep C18 filter plate with 40 μL bed volume (Thermo Fisher Scientific) and dried in vacuum.

Peptides were reconstituted in solvent A (0.1% FA in 2% ACN), and 2 μL of each sample was injected using an UltiMate 3000 Nano-Flow UHPLC system (Thermo Fisher Scientific). Peptides were captured on a 2 cm Acclaim PepMap Trap Column (Thermo Fisher Scientific) and separated on a heated (55°C) 50 cm long EASY-Spray C18 Column (Thermo Fisher Scientific) applying a 90-minute long gradient: 4%–26% of solvent B (0.1% FA in 98% ACN) in 90 minutes, 26%–95% in 5 minutes, and 95% of solvent B for 5 minutes at a flow rate of 300 nL/min.

Mass spectra were acquired on an Orbitrap Eclipse Tribrid Mass Spectrometer (Thermo Fisher Scientific) in *m/z* 300 to 1,500 at a resolution of *R* = 120,000 (at *m/z* 200) for full mass, targeting 6 × 10^5^ ions in maximum 100 ms, followed by data-dependent higher-energy collisional dissociation (HCD) fragmentations of precursor ions with a charge state 2+ to 6+, using 45 second dynamic exclusion. The tandem mass spectra of the top precursor ions were acquired in 3-second cycle time with a resolution of *R* = 30,000, targeting 1 × 10^5^ ions for a maximum injection time of 54 ms, setting quadrupole isolation width to 0.7 Th and normalized collision energy to 30%.

### Protein detection.

The raw files were searched against a human Uniprot database (downloaded on 2023-02-09, with 20,330 nonredundant entries) using an MS Amanda, version 2.0 (https://ms.imp.ac.at/?goto=msamanda), search engine loaded into Proteome Discoverer 3.0 software (Thermo Fisher Scientific). MS1 precursor mass tolerance was set at 10 ppm, and MS2 tolerance was set at 0.02 Da. The search criteria included a static carbamidomethylation of cysteines (+57.0214 Da) and variable modifications of oxidation (+15.9949 Da) on methionine residues, deamidation (+0.984 Da), asparagine, and glutamine residues. The search was performed with full trypsin/P digestion and allowed a maximum of 2 missed cleavages on the peptides analyzed from the sequence database. The false-discovery rates of proteins and peptides were set at 0.05. All protein and peptide identifications were grouped, and any redundant entries were removed. Unique peptides and unique master proteins were reported.

### Cell culture.

HCtAECs were purchased from Cell Applications Inc. (Cell Applications, Inc.) and maintained in endothelial cell growth medium (Cell Applications, Inc.), including 10% FBS (Biowest) and 2% penicillin-streptomycin. Cells were grown to confluence up to passage 8 to preserve endothelial cell features.

### Cell viability assay.

The viability of HCtAECs was determined by the colorimetry assay of the mitochondrial-dependent reduction of MTT (Sigma-Aldrich) to formazan. HCtAECs (2 × 10^5^ cells/mL) were seeded to each well of a 96-well plate. The next day, HCtAECs were coincubated with EVs derived from T2D-RBC-EVs and H-RBC-EVs for 24 hours in serum-free endothelial cell growth medium (Cell Applications, Inc.). Cells were then incubated with MTT (3 mg/mL in PBS) for 3 hours at 37°C. Medium was discarded, and dimethyl sulfoxide (200 μL) was added to each well to dissolve the formazan precipitate. Optical densities were measured at 2 wavelengths (560 and 650 nm) in a Victor2 microplate reader (PerkinElmer).

### Endothelial cell–EV coincubation.

HCtAECs were cultured in precoated 6-well plates until they reached 70%–80% confluence. Afterward, freshly isolated EVs from H-RBCs and T2D-RBCs were incubated with HCtAECs in serum-free endothelial cell growth medium (Cell Applications, Inc.) at 37°C and 5% CO_2_ for 8 hours and 24 hours. HCtAECs were then washed several times with ice-cold PBS (Gibco, Thermo Fisher Scientific), detached with QIAzol Lysis Reagent (QIAGEN) or RIPA lysis buffer, and immediately stored at –80°C for subsequent RNA or protein extraction.

### Arginase activity assay.

HCtAECs were lysed using RIPA lysis buffer (VWR International) containing protease inhibitors (Roche). Arginase activity was determined by a colorimetric assay as previously described (4). Briefly, 75 μL of 10 mM MnCl_2_ (mixed with 50 mM Tris) with pH 7.5 were added to 50 μL per sample. The mixture was heated to 56°C for 10 minutes to activate arginase, and the arginine hydrolysis was achieved by adding 50 μL of 0.5 M l-arginine dissolved in 50 mM Tris, pH 9.7, and then incubated at 37°C for 60 minutes. The hydrolysis was stopped by adding 400 μL of stop-solution (H_2_SO_4_:H_3_PO_4_:H_2_O 1:3:7). Afterward, 25 μL of 9% α-isonitrosopropiophenone (diluted in ethanol) was added to the mixture and incubated at 100°C for 60 minutes. The samples were then loaded onto a 0.22 μm centrifugal filter (Ultrafree; Sigma-Aldrich) and centrifuged for 5 minutes at 5,000*g* at room temperature. The urea concentration in the filtrate was determined in a spectrophotometer Victor2 microplate reader (PerkinElmer) at 540 nm. Arginase activity was calculated as urea production (mmol urea/mg protein/min).

### Arginase-1 silencing via siRNA.

60%–70% confluent HCtAECs were transfected with either a control siRNA or with an arginase-1–specific siRNA (50 nM; both predesigned by Ambion, Life Technologies) using Lipofectamine RNAiMAX (Invitrogen). Twenty-four hours after the transfection, HCtAECs were coincubated with freshly isolated T2D RBC-EVs in serum-free endothelial cell growth medium (Cell Applications Inc.) at 37°C and 5% CO_2_ for an additional 24 hours. Afterward, HCtAECs were washed with ice-cold PBS (Gibco, Thermo Fisher Scientific) 48 hours after transfection, detached with QIAzol Lysis Reagent (QIAGEN), and immediately stored at –80°C for subsequent RNA extraction.

### Quantitative PCR.

RNA extraction was performed using the QIAGEN miRNeasy Mini Kit (QIAGEN). Total RNA was assessed using a NanoDrop 2000 spectrophotometer (Thermo Fisher Scientific). cDNA was synthesized by using the TaqMan High-Capacity cDNA Transcription Kit (Applied Biosystems). PCR amplification reactions were performed in QuantStudio 7 Pro Real-Time PCR System (Applied Biosystems) using the TaqMan Universal PCR Master Mix II with UNG (Applied Biosystems) following the manufacturer’s instructions. The resulting cDNA was amplified with specific TaqMan Gene Expression probes for arginase-1 (*ARG1*; Hs00968981_g1), arginase-2 (*ARG2*; Hs00982833_m1), *NOX1* (Hs01071088_m1), and *NOX4* (Hs01379108_m1).

The relative quantification of the transcript was determined with the 2^–ΔΔCt^ method, using ribosomal protein lateral stalk subunit P0 (*RPLP0*; Hs99999902_m1) as endogenous control and normalizing to the control group. Applied Biosystems predesigned all probes. All samples were measured in duplicate.

### Immunocytochemistry.

HCtAECs were grown to confluence on glass coverslips. In some experiments, cells were transfected with either a control siRNA or with arginase-1–specific siRNA (50 nM; Ambion, Life Technologies) for 24 hours and coincubated with freshly isolated T2D RBC-EVs in serum-free endothelial cell growth medium (Cell Applications, Inc.) at 37°C and 5% CO_2_ for a further 24 hours. Cells were then washed twice with ice-cold PBS, fixed with 4% paraformaldehyde, and blocked in a 3% BSA/PBS solution. HCtAECs were incubated with a rabbit polyclonal anti-human arginase-1 antibody (1:100 dilution, catalog no. HPA003595; Atlas Prestige Antibody, Sigma-Aldrich). To confirm antibody specificity, a rabbit IgG polyclonal isotype control was used (catalog no. ab37415; Abcam). A secondary Alexa Fluor 488–conjugated goat anti-rabbit antibody (1:200 dilution, catalog no. A11034; Invitrogen, Life Technologies) was used for 1 hour at room temperature. Cell nuclei were counterstained with Hoechst 33344 dye for 30 minutes at room temperature (1:500 dilution; Sigma-Aldrich). Images were captured with a Leica DM3000 digital microscope (Leica Biosystems), digitized, and analyzed (ImageJ software, version 2.0, NIH).

### In vitro uptake of EVs.

HCtAECs were seeded in 8-well chamber slides (Thermo Fisher Scientific) and cultured in endothelial cell growth medium (Cell Applications Inc.) supplemented with 10% FBS and 2% penicillin-streptomycin until they reached 80%–90% confluence.

To study the uptake of RBC-derived EVs by endothelial cells, EVs were isolated using the exoEasy Maxi Kit as explained above, and their cell membranes were labeled with aliphatic tails (PKH67) according to the manufacturer’s instructions (Sigma-Aldrich). Briefly, diluted EVs were incubated with PKH67 for 5 minutes. Then, EVs were washed twice with PBS and centrifuged at 3,000*g* for 5 minutes. Lastly, EVs were diluted in serum-free endothelial cell growth medium (Cell Applications Inc.) before being coincubated with HCtAECs for 24 hours in serum-free endothelial cell growth medium to determine the uptake of the EVs. PKH67 alone was used as a control for dye aggregates. Heparin (0.3 μg/mL) was added to some wells to block the uptake of EVs by the endothelial cells. In another set of experiments, after 24 hours coincubation with EVs, HCtAECs were fixed, blocked, and incubated with an Alexa Fluor 594–conjugated mouse monoclonal antibody against RAB5A (1:100 dilution, IgG_2b_, clone 3A4, catalog no. NBP1-04340AF594; Novus Biologicals) for 2 hours at room temperature. Afterward, the cells were washed, and cell nuclei were counterstained with Hoechst 33344 dye (1:1,000 dilution; Sigma-Aldrich) and mounted with SlowFade Gold antifade reagent (Molecular Probes). Images from at least 6 fields were taken by a confocal microscope (Nikon ECLIPSE Ti2; Nikon) and then processed and analyzed using the software ImageJ (Fiji software, version 2.0).

### Library preparation, bulk RNA-Seq, and data analysis.

The libraries for bulk RNA-Seq were prepared using prime-seq as previously described (28). The Qubit dsDNA HS Assay Kit (Thermo Fisher Scientific) was used for the determination of cDNA and library concentrations, and quality control of cDNA size and library size selection was done using the Bioanalyzer High Sensitivity DNA Kit (Agilent Technologies) according to the manufacturer’s instructions. The final library was subsequently sequenced on an Illumina NovaSeq 6000 at an average depth of 10 million reads per sample at Novogene Ltd.

Raw data obtained from bulk RNA-Seq was processed as previously described (29). Afterward, data analysis was performed in R, version 4.3.0. Quality control of the samples (amount of reads per sample, genes detected, and mitochondrial gene count) was performed in Seurat, version 4.3.0 (30), and all samples passed quality control. Subsequently, differential gene expression analysis was performed using DESeq2, version v.1.40.2 (31). Mitochondrial genes and genes with a low expression (summed gene count ≤ 20) were removed from further analysis. Differentially expressed genes were defined by adjusted *P* value < 0.05. Thereafter, whole gene lists obtained from DESeq2 after log*_2_* fold change shrinking using ashr (32) were used for GSEA using hallmark genesets from the Molecular Signatures Database (MSigDB, version 2023.2) and clusterProfiler, version 4.8.2 (33–35).

### Statistics.

Statistical analysis was calculated using GraphPad Prism 10 (GraphPad Software Inc.). The distribution of data was tested using the D’Agostino-Pearson test and Shapiro-Wilk test. Normally distributed data are presented as mean ± SD, while data that were not normally distributed are presented as median ± interquartile range (Q1–Q3). Categorical data are reported as numbers and percentages. Differences in concentration-dependent relaxations were analyzed using a 2-way ANOVA with repeated measures. Multiple comparisons were performed using 1-way ANOVA or 2-way ANOVA, followed by post hoc analysis using Bonferroni’s test. Differences between 2 groups were performed using an unpaired or paired 2-tailed *t* test or nonparametric Mann-Whitney *U* test, depending on the distribution of data, or Fisher’s exact test. The number of experimental observations (*n*) refers to the number of animals and cell culture experiments. *P* < 0.05 was considered statistically significant.

### Study approval.

The protocols were approved by the Swedish Ethical Review Authority for human studies (2014_0463-31 and 2016/2283-32), and all procedures were conducted according to the Declaration of Helsinki. All participants were informed of the study’s purpose and gave their oral and written informed consent before any study-related procedures were initiated. Animal protocols were approved by the Regional Ethics Committee of Stockholm (17708-2019 and 20380-22) and conformed to the *Guide for the Care and Use of Laboratory Animals* (National Academies Press, 2011).

### Data availability.

Values for all data points in graphs are reported in the Supporting Data Values file. The raw RNA-Seq data reported in this paper have been deposited under the Bioproject accession no. PRJNA1186397. Other data are available from the corresponding author upon reasonable request.

## Author contributions

AC, ME, SG, PE, ZZ, and JP conceptualized and supervised the study. AC, RH, EK, ME, JS, TJ, ED, AZ, AV, and ZZ performed and collected research data. AC, ME, AZ, and QD analyzed research data and performed statistical analysis. AC, RH, EK, ME, JS, JY, TJ, and ED provided animal and methodology resources. EC, AM, MA, and JP recruited patients and collected samples. AC and JP drafted the manuscript. RH, EK, ME, JS, AZ, JY, TJ, ED, EC, AM, JT, AV, QD, MA, SG, PE, and ZZ edited the manuscript. The order of the co–first authors was based on the role of AC in the conceptualization of the study and draft of the manuscript. All authors have read and approved the final version of the manuscript.

## Supplementary Material

Supplemental data

Unedited blot and gel images

Supporting data values

## Figures and Tables

**Table 1:**
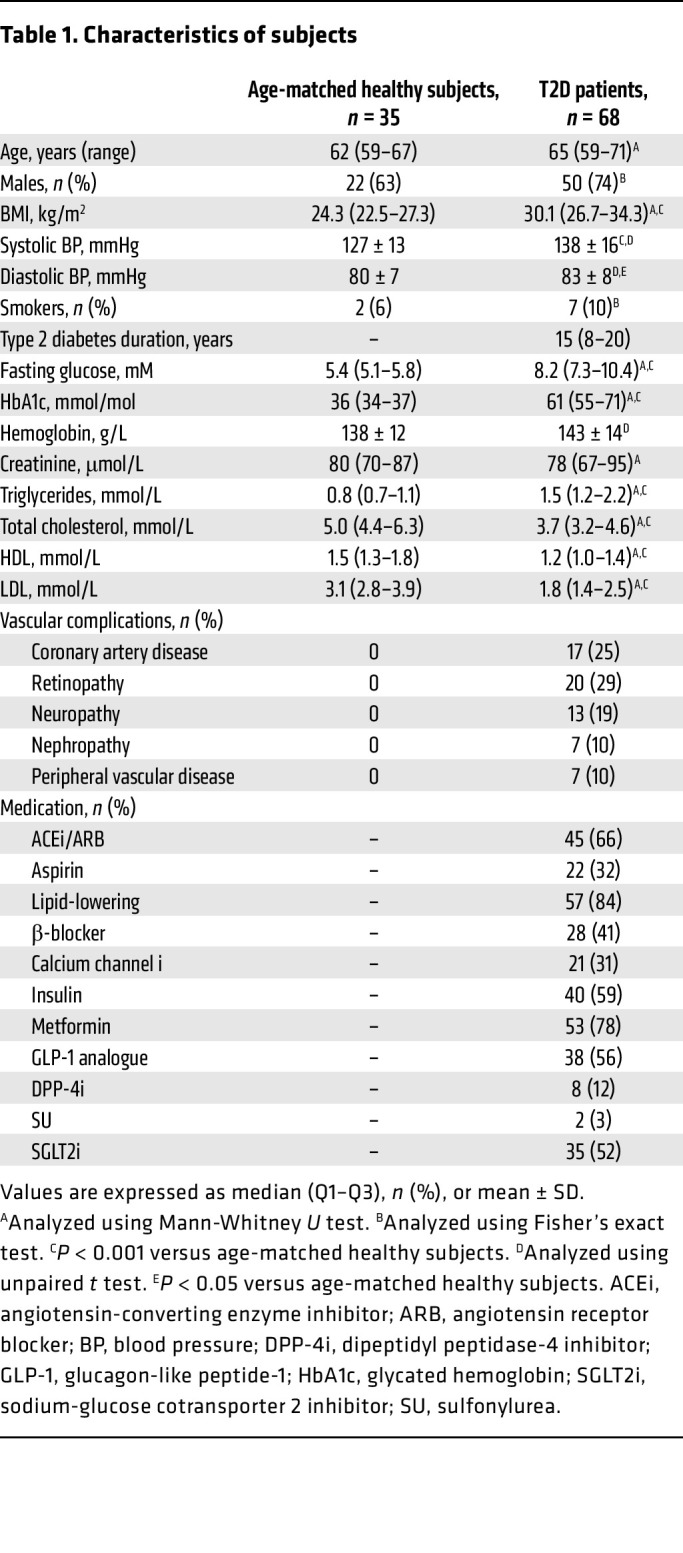
Characteristics of subjects

## Version 1 03/20/2025

In-Press Preview

## Version 2 05/15/2025

Electronic publication
