# Supplementary material for: Erythrocyte-derived extracellular vesicles induce endothelial dysfunction through arginase-1 and oxidative stress in type 2 diabetes
Source: J Clin Invest. 2025 Mar 20;135(10):e180900. doi: 10.1172/JCI180900 (PMC12077887; doi:10.1172/JCI180900)
Supplement: Supplemental data [file jci-135-180900-s277.pdf]

SUPPLEMENTAL FIGURES

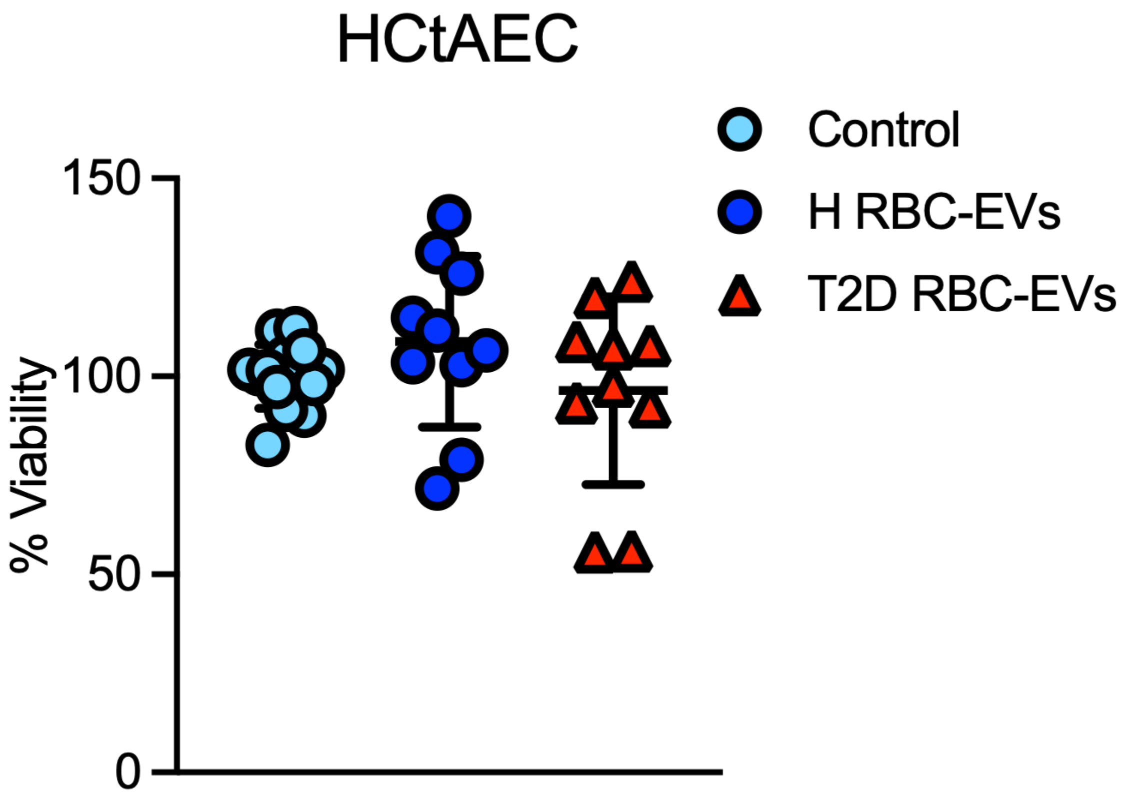

Supplemental Figure 1. Effect of red blood cell (RBC)-derived extracellular vesicles (EVs) on cell viability. Viability (%) of human carotid artery endothelial cells (HCtAEC) in the presence of Krebs-Henseleit (KH) buffer (control), released EVs from healthy RBCs (H RBC-EVs), and EVs from RBCs of patients with type 2 diabetes (T2D RBC-EVs) for 24h and determined by the MTT method. Values are expressed as mean ± SD (n=10-14).

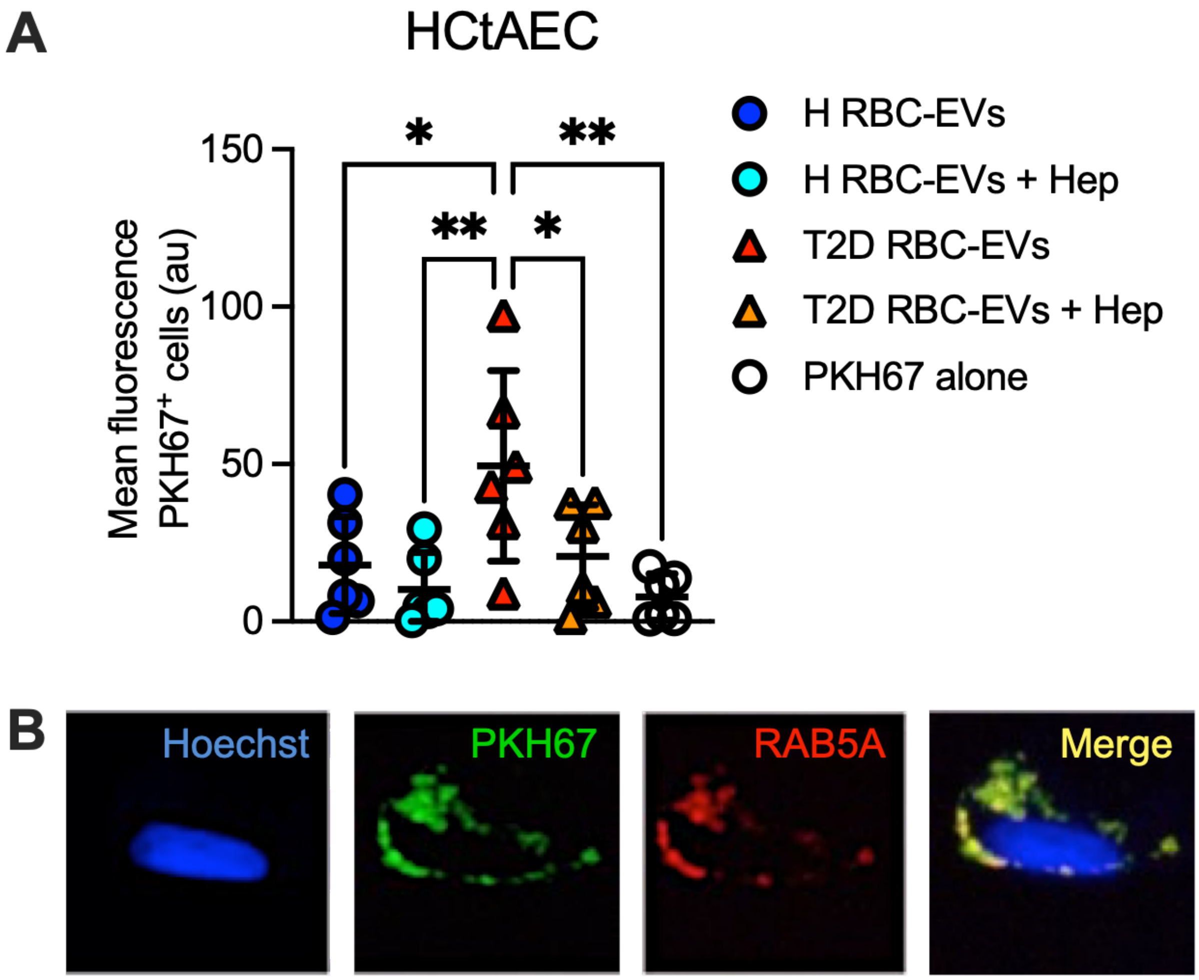

Supplemental Figure 2. Quantitative analyses of the mean fluorescence PKH67^{+} cells (arbitrary units; au) after 24h co-incubation with HCtAEC with or without heparin (Hep) treatment (**A**, n=6). Representative immunofluorescence images depicting co-localization of PKH67 (green) and intracellular endosomal marker RAB5A (red) in HCtAEC following 24h co-incubation with H RBC-EVs and T2D RBC-EVs. Nuclei were stained with Hoechst (**B**). Values are expressed as mean ± SD. *$P<0.05$ or **$P<0.01$ using one-way analyses of variance (ANOVA).

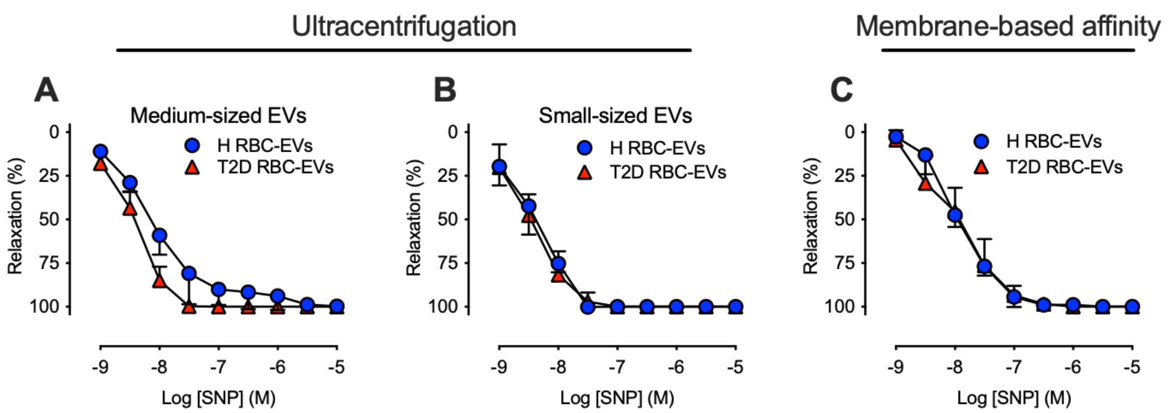

Supplemental Figure 3. Endothelium-independent relaxation (EIR) evoked by sodium nitroprusside (SNP) in mouse aortas following 18h of co-incubation with H RBC-EVs or T2D RBC-EVs and isolated by sequential ultracentrifugation (**A**, medium-sized EVs, n=3-4 and **B**, small-sized EVs, n=3-9) or membrane-base affinity (**C**, n=5-6 in each group). Values are expressed as mean and SD.

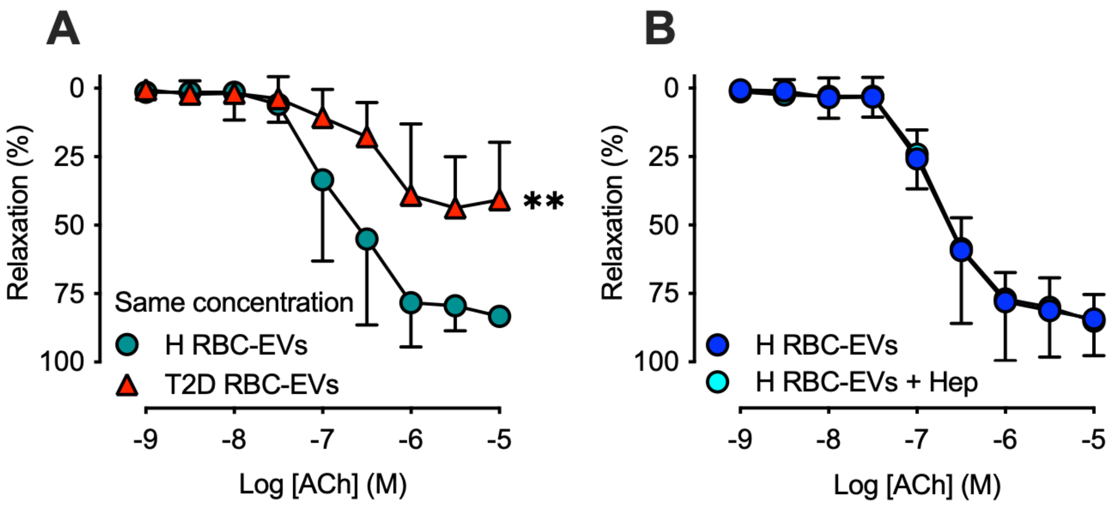

Supplemental Figure 4. Endothelium-dependent relaxation (EDR) evoked by acetylcholine (ACh) in mouse aortic rings following 18h of co-incubation with H RBC-EVs or T2D RBC-EVs at the same concentration (**A**, n=4-7). EDR evoked by ACh in mouse aortas following 18h of co-incubation with H RBC-EVs and heparin (**B**, n=5). Values are expressed as mean and SD. ***P*<0.01 using repeated measures two-way ANOVA in **A**.

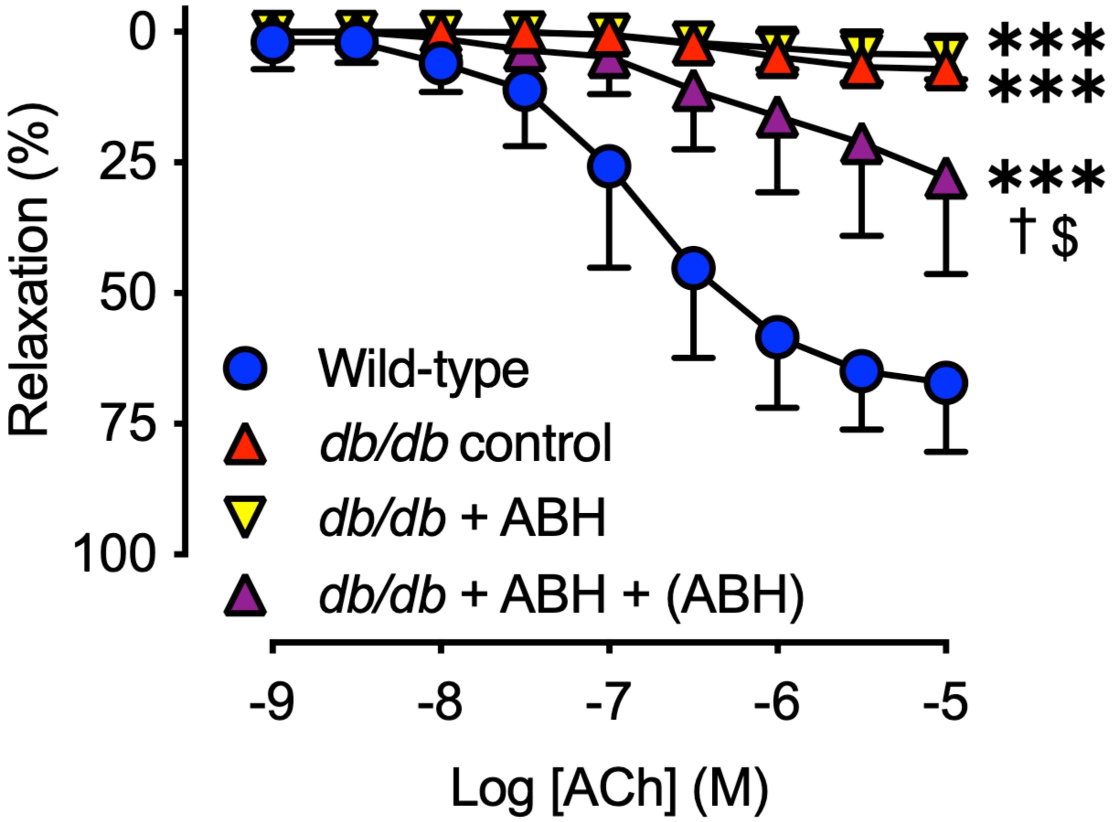

Supplemental Figure 5. Effects of arginase inhibitor ABH on endothelial function in mice with type 2 diabetes (*db/db*). EDR evoked by ACh in aortas isolated from wild-type mice or *db/db* mice following incubation with vehicle or the arginase inhibitor ABH (n=8-10) during the 18h incubation (*db/db* control and *db/db* + ABH, respectively). Following the incubation, EDR was evaluated in the presence of ABH also in the myograph (*db/db* + ABH + (ABH); n=9-10). Values are expressed as mean and SD. ****P*<0.001 vs. wild-type, †*P*<0.05 vs. *db/db* control, and $*P*<0.05 vs. *db/db* + ABH using repeated measures two-way ANOVA. Parentheses indicate that the inhibitor was added in the organ baths for 1h following the 18h incubation.

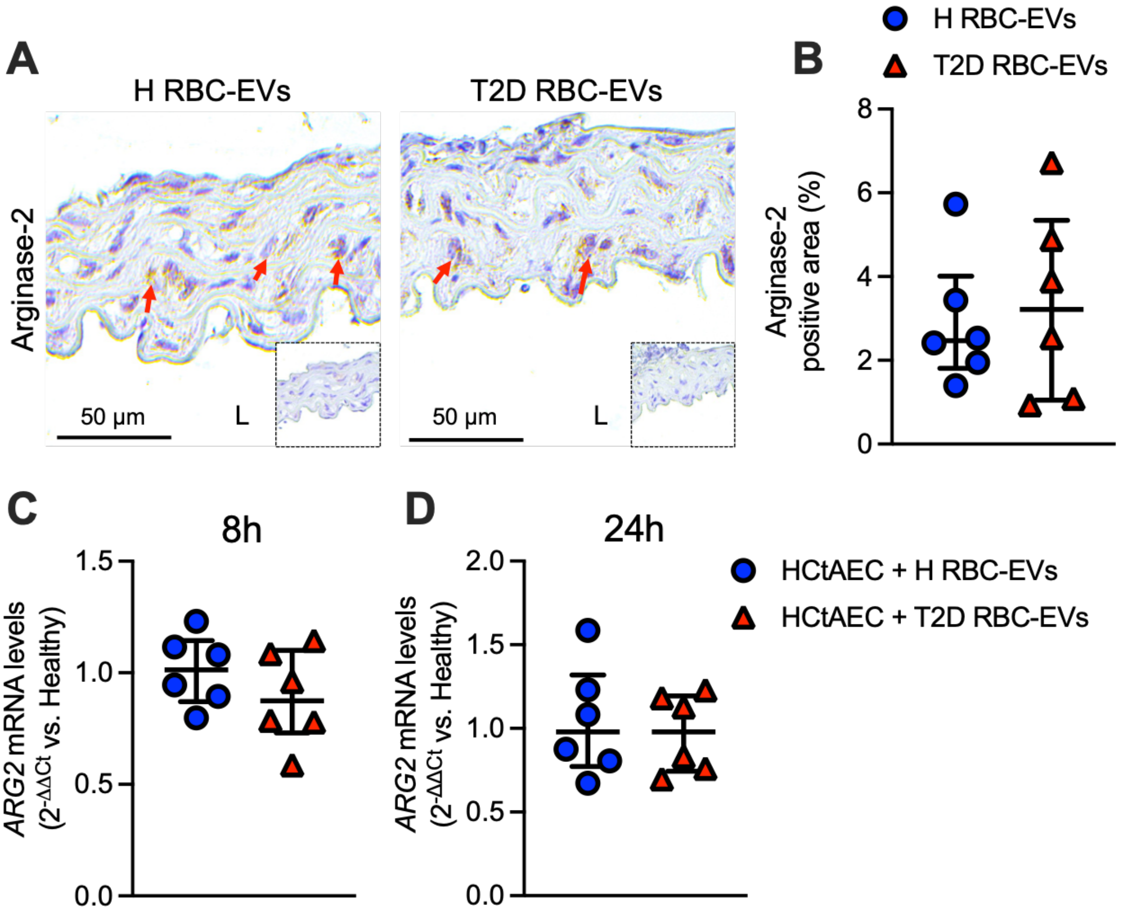

Supplemental Figure 6. Representative immunohistochemical images depicting arginase-2 in mouse aortic rings following 18h co-incubation with H RBC-EVs and T2D RBC-EVs (**A**). IgG controls are presented in inserts for each experimental condition. L indicates the luminal side of the vessel, and red arrows indicate smooth muscle cells. Quantitative analyses of positivity of the total area in mouse aortas for arginase-2 (**B**, n=6). mRNA expression levels of arginase 2 (*ARG2*) after co-incubation of HCtAEC with H RBC-EVs and T2D RBC-EVs for 8h (**C**, n=6) and 24h (**D**, n=6). Values are expressed as median ± interquartile range (Q1-Q3).

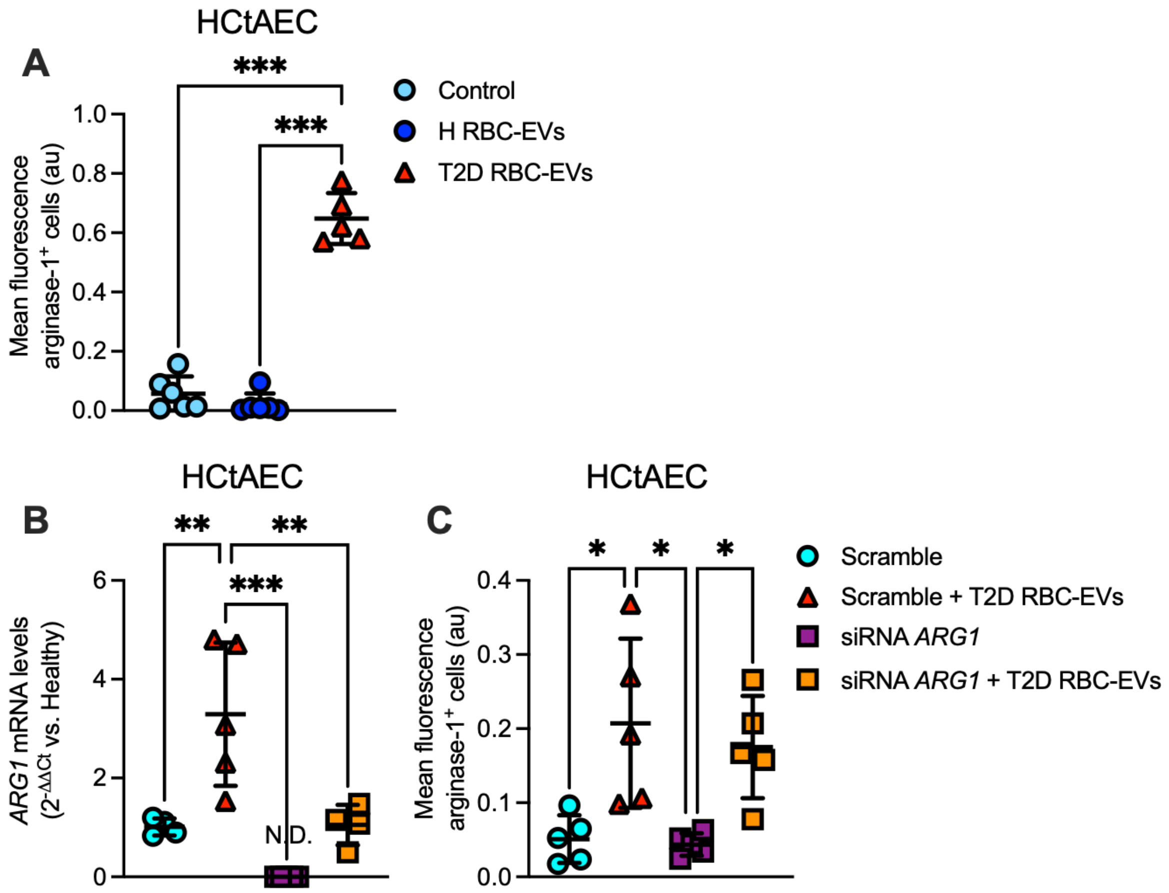

Supplemental Figure 7. Quantitative analyses of the mean fluorescence (arbitrary units; au) for arginase-1 positive HCtAEC after 24h co-incubation with H RBC-EVs and T2D RBC-EVs (**A**, n=5-6). mRNA expression levels of arginase 1 (*ARG1*) in HCtAEC after 24h transfection and 24h of co-incubation with T2D RBC-EVs (**B**, n=4-5). Quantitative analyses of the mean fluorescence (arbitrary units; au) for arginase-1 positive HCtAEC after 24h transfection and 24h co-incubation with T2D RBC-EVs (**C**, n=5). N.D.= Not detected. Values are expressed as mean ± SD. **P*<0.05, ***P*<0.01, or ****P*<0.001 using one-way ANOVA in **A**, **B**, and **C**.

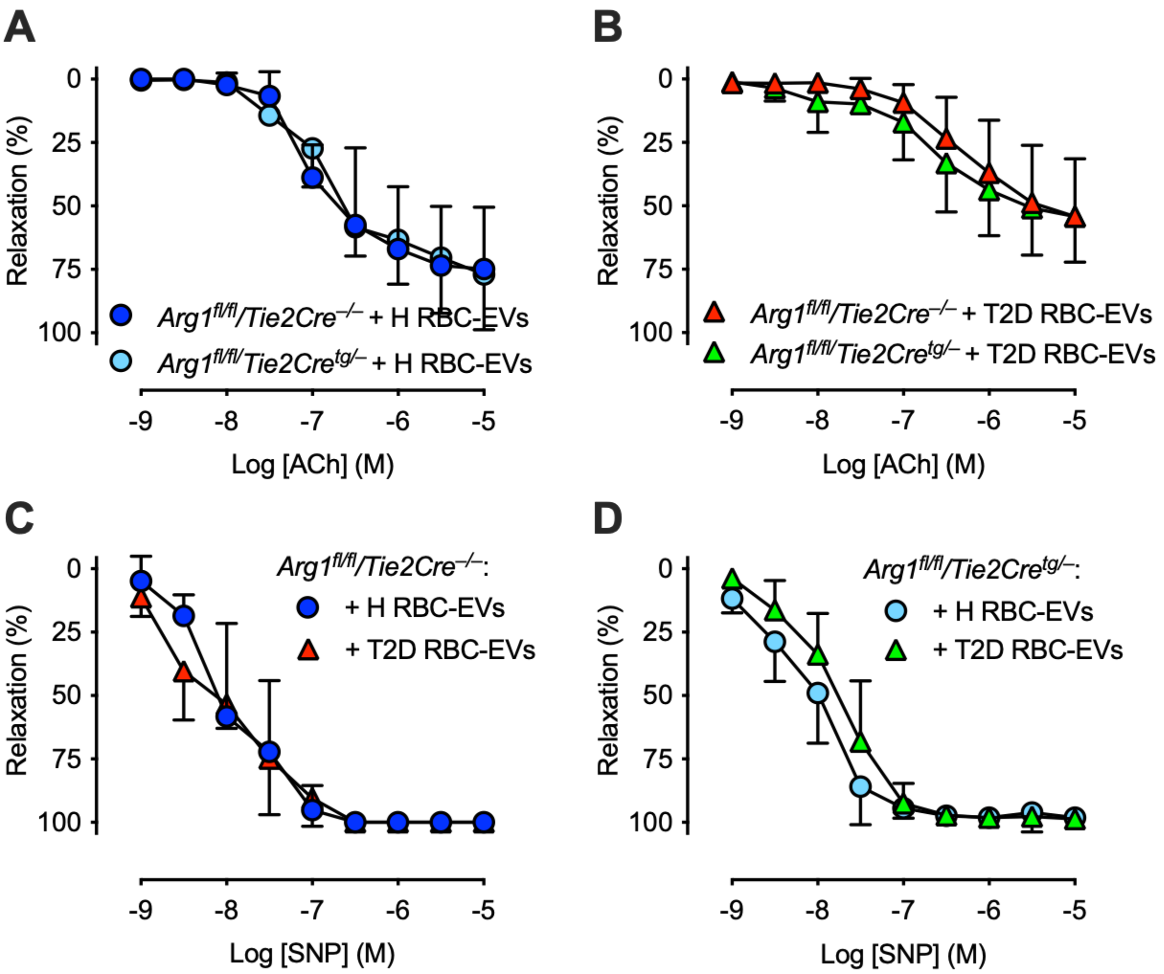

Supplemental Figure 8. EDR evoked by ACh in aortic rings from endothelial cell arginase 1 knockout (KO) mice ($Arg1^{fl/fl}/Tie2Cre^{tg/-}$) and their littermates ($Arg1^{fl/fl}/Tie2Cre^{-/-}$) following 18h of co-incubation with H RBC-EVs (**A**, n=4). EDR evoked by ACh in aortas from $Arg1^{fl/fl}/Tie2Cre^{tg/-}$ and $Arg1^{fl/fl}/Tie2Cre^{-/-}$ mice following 18h of co-incubation with T2D RBC-EVs (**B**, n=5). EIR evoked by SNP in aortas from $Arg1^{fl/fl}/Tie2Cre^{-/-}$ mice following 18h of co-incubation with H RBC-EVs or T2D RBC-EVs (**C**, n=4). EIR evoked by SNP in aortas from $Arg1^{fl/fl}/Tie2Cre^{tg/-}$ mice following 18h of co-incubation with H RBC-EVs or T2D RBC-EVs (**D**, n=4). Values are expressed as mean and SD.

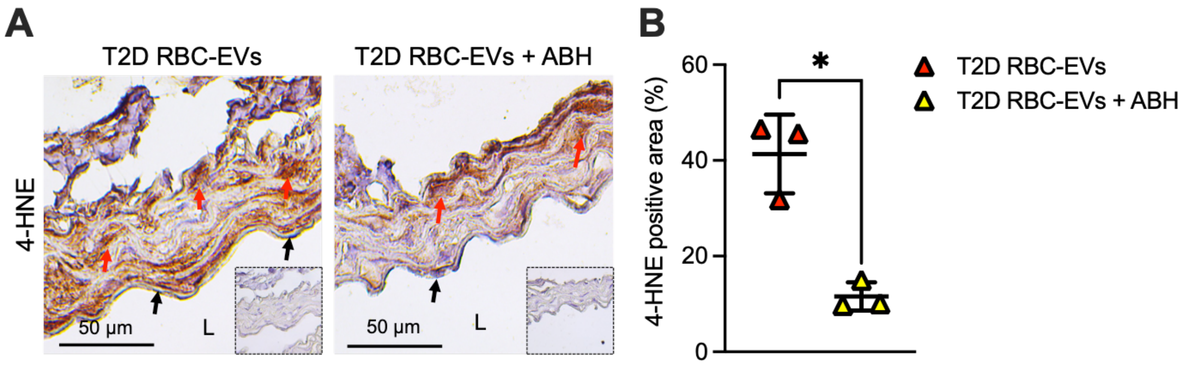

Supplemental Figure 9. Representative immunohistochemical images depicting 4-hydroxynonenal (4-HNE) in mouse aortic rings following 18h co-incubation with T2D RBC-EVs and T2D RBC-EVs + ABH (**A**). IgG controls are presented in inserts for each experimental condition. L indicates the luminal side of the vessel, black arrows endothelial cells, and red arrows smooth muscle cells. Quantitative analyses of positivity of the total area in mouse aortas for 4-HNE (**B**, n=3). Values are expressed as mean ± SD. **P*<0.05 using paired t-test in **B**.

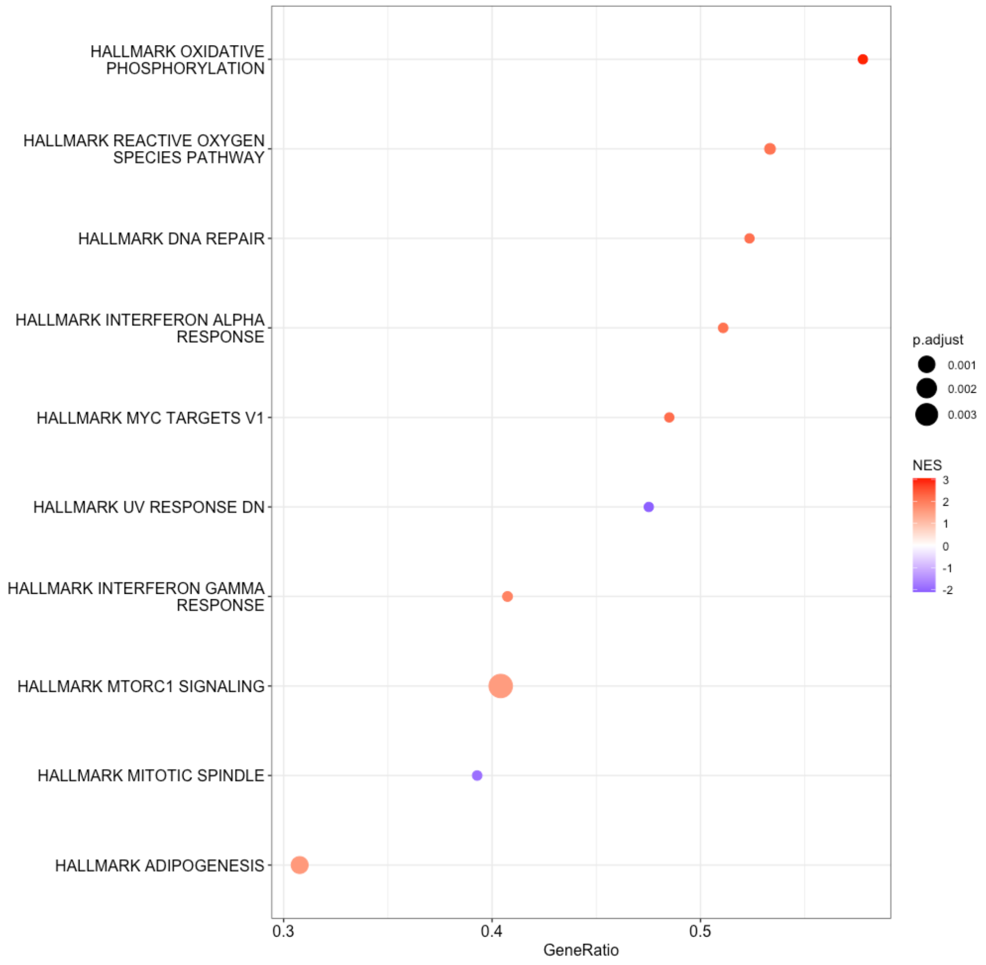

Supplemental Figure 10. Dotplot depicting significantly enriched Hallmark gene sets generated by gene set enrichment analysis (GSEA) comparing the transcriptome of HCtAEC co-incubated 24h with EVs derived from H-RBCs (n=6) and T2D-RBCs (n=6).

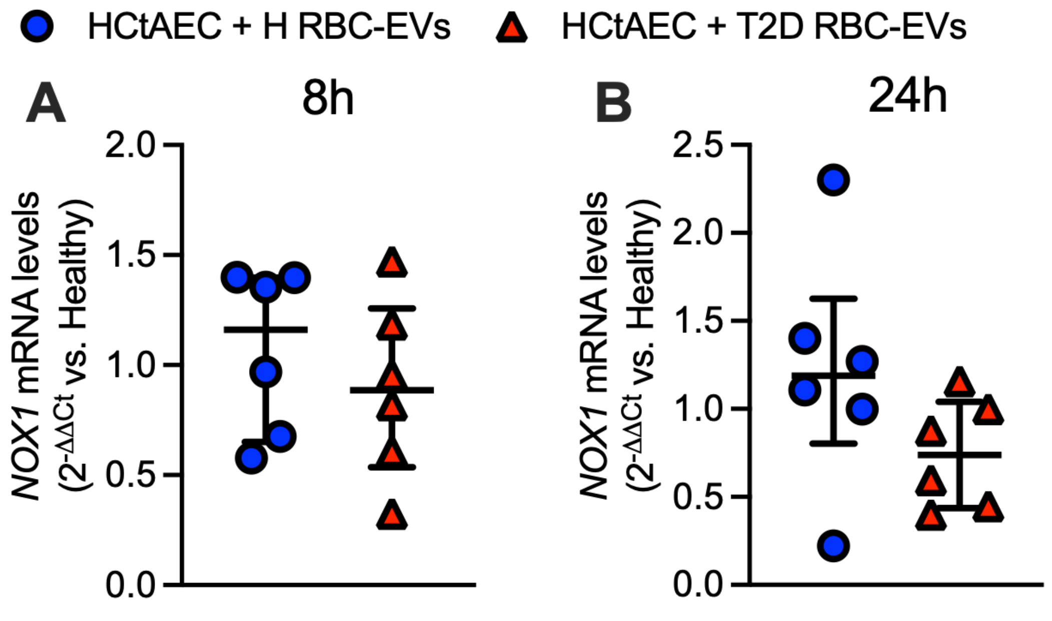

Supplemental Figure 11. mRNA expression levels of NADPH oxidase 1 (*NOX1*) after co-incubation of HCtAEC with H RBC-EVs and T2D RBC-EVs for 8h (**A**, n=6) and 24h (**B**, n=6). Values are expressed as median ± interquartile range (Q1-Q3).

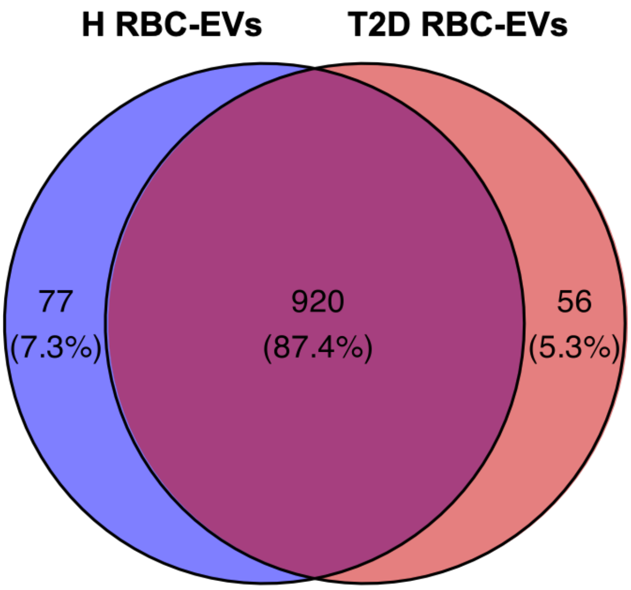

Supplemental Figure 12. Venn diagram. The number of identified proteins and the percentage of the total number of identified proteins in each data set.

SUPPLEMENTAL TABLES

Supplemental Table 1. Characteristics for IMA donors, n=4

Age, years (range)	61 ± 6 (55-68)
Males, n (%)	2 (50)
BMI, kg/m^2	26.9 ± 5.8
Systolic BP, mmHg	127 ± 15
Diastolic BP, mmHg	81 ± 6
Smokers, n (%)	0 (0)
Fasting glucose, mM	5.5 ± 0.9
HbA1c, mmol/mol	37 ± 4
Hemoglobin, g/L	117 ± 23
Creatinine, µmol/L	71 ± 20
Triglycerides, mmol/L	1.4 ± 0.6
Total cholesterol, mmol/L	4.9 ± 1.8
HDL, mmol/L	1.2 ± 0.3
LDL, mmol/L	3.1 ± 1.5
Medication, n (%)	
ACEi/ARB	2 (50)
Aspirin	3 (75)
Lipid-lowering	3 (75)
β-blocker	2 (50)
Calcium channel i	1 (25)

Values are expressed as mean ± SD or n (%). ACEi=angiotensin-converting enzyme inhibitor; ARB=angiotensin receptor blocker; BMI=body mass index; BP=blood pressure;

HbA1c=glycated hemoglobin; HDL=high-density lipoprotein; IMA=internal mammary artery; LDL=low-density lipoprotein.

Supplemental Table 2. Proteins detected in H-RBC- and T2D-RBC-derived EVs by LC-MS/MS

Protein	Accession	Description
A1BG	P04217	Alpha-1B-glycoprotein OS=Homo sapiens OX=9606 GN=A1BG PE=1 SV=4
A2M	P01023	Alpha-2-macroglobulin OS=Homo sapiens OX=9606 GN=A2M PE=1 SV=3
ABI1	Q8IZP0	Abl interactor 1 OS=Homo sapiens OX=9606 GN=ABI1 PE=1 SV=4
ACAP1	Q15027	Arf-GAP with coiled-coil, ANK repeat and PH domain-containing protein 1 OS=Homo sapiens OX=9606 GN=ACAP1 PE=1 SV=1
ACIN1	Q9UKV3	Apoptotic chromatin condensation inducer in the nucleus OS=Homo sapiens OX=9606 GN=ACIN1 PE=1 SV=2
ACLY	P53396	ATP-citrate synthase OS=Homo sapiens OX=9606 GN=ACLY PE=1 SV=3
ACP1	P24666	Low molecular weight phosphotyrosine protein phosphatase OS=Homo sapiens OX=9606 GN=ACP1 PE=1 SV=3
ACTB	P60709	Actin, cytoplasmic 1 OS=Homo sapiens OX=9606 GN=ACTB PE=1 SV=1
ACTC1	P68032	Actin, alpha cardiac muscle 1 OS=Homo sapiens OX=9606 GN=ACTC1 PE=1 SV=1
ACTN1	P12814	Alpha-actinin-1 OS=Homo sapiens OX=9606 GN=ACTN1 PE=1 SV=2
ACTN4	O43707	Alpha-actinin-4 OS=Homo sapiens OX=9606 GN=ACTN4 PE=1 SV=2
ACTR1B	P42025	Beta-centractin OS=Homo sapiens OX=9606 GN=ACTR1B PE=1 SV=1
ACTR2	P61160	Actin-related protein 2 OS=Homo sapiens OX=9606 GN=ACTR2 PE=1 SV=1
ACTR3	P61158	Actin-related protein 3 OS=Homo sapiens OX=9606 GN=ACTR3 PE=1 SV=3
ADAM10	O14672	Disintegrin and metalloproteinase domain-containing protein 10 OS=Homo sapiens OX=9606 GN=ADAM10 PE=1 SV=1
ADD1	P35611	Alpha-adducin OS=Homo sapiens OX=9606 GN=ADD1 PE=1 SV=2
AFM	P43652	Afamin OS=Homo sapiens OX=9606 GN=AFM PE=1 SV=1
AGT	P01019	Angiotensinogen OS=Homo sapiens OX=9606 GN=AGT PE=1 SV=3
AHCY	P23526	Adenosylhomocysteinase OS=Homo sapiens OX=9606 GN=AHCY PE=1 SV=4
AHNAK	Q09666	Neuroblast differentiation-associated protein AHNAK OS=Homo sapiens OX=9606 GN=AHNAK PE=1 SV=2
AHSG	P02765	Alpha-2-HS-glycoprotein OS=Homo sapiens OX=9606 GN=AHSG PE=1 SV=2
AHSP	Q9NZD4	Alpha-hemoglobin-stabilizing protein OS=Homo sapiens OX=9606 GN=AHSP PE=1 SV=1
AK1	P00568	Adenylate kinase isoenzyme 1 OS=Homo sapiens OX=9606 GN=AK1 PE=1 SV=3

Protein	Accession	Description
AK2	P54819	Adenylate kinase 2, mitochondrial OS=Homo sapiens OX=9606 GN=AK2 PE=1 SV=2
ALAD	P13716	Delta-aminolevulinic acid dehydratase OS=Homo sapiens OX=9606 GN=ALAD PE=1 SV=1
ALDH1A1	P00352	Aldehyde dehydrogenase 1A1 OS=Homo sapiens OX=9606 GN=ALDH1A1 PE=1 SV=2
ALDOA	P04075	Fructose-bisphosphate aldolase A OS=Homo sapiens OX=9606 GN=ALDOA PE=1 SV=2
AMBP	P02760	Protein AMBP OS=Homo sapiens OX=9606 GN=AMBP PE=1 SV=1
AMPD3	Q01432	AMP deaminase 3 OS=Homo sapiens OX=9606 GN=AMPD3 PE=1 SV=1
ANK1	P16157	Ankyrin-1 OS=Homo sapiens OX=9606 GN=ANK1 PE=1 SV=3
ANP32A	P39687	Acidic leucine-rich nuclear phosphoprotein 32 family member A OS=Homo sapiens OX=9606 GN=ANP32A PE=1 SV=1
ANP32B	Q92688	Acidic leucine-rich nuclear phosphoprotein 32 family member B OS=Homo sapiens OX=9606 GN=ANP32B PE=1 SV=1
ANP32E	Q9BTT0	Acidic leucine-rich nuclear phosphoprotein 32 family member E OS=Homo sapiens OX=9606 GN=ANP32E PE=1 SV=1
ANXA1	P04083	Annexin A1 OS=Homo sapiens OX=9606 GN=ANXA1 PE=1 SV=2
ANXA11	P50995	Annexin A11 OS=Homo sapiens OX=9606 GN=ANXA11 PE=1 SV=1
ANXA2	P07355	Annexin A2 OS=Homo sapiens OX=9606 GN=ANXA2 PE=1 SV=2
ANXA3	P12429	Annexin A3 OS=Homo sapiens OX=9606 GN=ANXA3 PE=1 SV=3
ANXA4	P09525	Annexin A4 OS=Homo sapiens OX=9606 GN=ANXA4 PE=1 SV=4
ANXA5	P08758	Annexin A5 OS=Homo sapiens OX=9606 GN=ANXA5 PE=1 SV=2
ANXA6	P08133	Annexin A6 OS=Homo sapiens OX=9606 GN=ANXA6 PE=1 SV=3
ANXA7	P20073	Annexin A7 OS=Homo sapiens OX=9606 GN=ANXA7 PE=1 SV=3
APBB1IP	Q7Z5R6	Amyloid beta A4 precursor protein-binding family B member 1-interacting protein OS=Homo sapiens OX=9606 GN=APBB1IP PE=1 SV=1
APCS	P02743	Serum amyloid P-component OS=Homo sapiens OX=9606 GN=APCS PE=1 SV=2
APEH	P13798	Acylamino-acid-releasing enzyme OS=Homo sapiens OX=9606 GN=APEH PE=1 SV=4
API5	Q9BZZ5	Apoptosis inhibitor 5 OS=Homo sapiens OX=9606 GN=API5 PE=1 SV=3
APLP2	Q06481	Amyloid beta precursor like protein 2 OS=Homo sapiens OX=9606 GN=APLP2 PE=1 SV=2
APOA1	P02647	Apolipoprotein A-I OS=Homo sapiens OX=9606 GN=APOA1 PE=1 SV=1

Protein	Accession	Description
APOA2	P02652	Apolipoprotein A-II OS=Homo sapiens OX=9606 GN=APOA2 PE=1 SV=1
APOA4	P06727	Apolipoprotein A-IV OS=Homo sapiens OX=9606 GN=APOA4 PE=1 SV=4
APOB	P04114	Apolipoprotein B-100 OS=Homo sapiens OX=9606 GN=APOB PE=1 SV=2
APOBR	Q0VD83	Apolipoprotein B receptor OS=Homo sapiens OX=9606 GN=APOBR PE=1 SV=3
APOC1	P02654	Apolipoprotein C-I OS=Homo sapiens OX=9606 GN=APOC1 PE=1 SV=1
APOC2	P02655	Apolipoprotein C-II OS=Homo sapiens OX=9606 GN=APOC2 PE=1 SV=1
APOC3	P02656	Apolipoprotein C-III OS=Homo sapiens OX=9606 GN=APOC3 PE=1 SV=1
APOC4	P55056	Apolipoprotein C-IV OS=Homo sapiens OX=9606 GN=APOC4 PE=1 SV=1
APOD	P05090	Apolipoprotein D OS=Homo sapiens OX=9606 GN=APOD PE=1 SV=1
APOE	P02649	Apolipoprotein E OS=Homo sapiens OX=9606 GN=APOE PE=1 SV=1
APOF	Q13790	Apolipoprotein F OS=Homo sapiens OX=9606 GN=APOF PE=1 SV=2
APOH	P02749	Beta-2-glycoprotein 1 OS=Homo sapiens OX=9606 GN=APOH PE=1 SV=3
APOL1	O14791	Apolipoprotein L1 OS=Homo sapiens OX=9606 GN=APOL1 PE=1 SV=5
APOM	O95445	Apolipoprotein M OS=Homo sapiens OX=9606 GN=APOM PE=1 SV=2
APP	P05067	Amyloid-beta precursor protein OS=Homo sapiens OX=9606 GN=APP PE=1 SV=3
AQP1	P29972	Aquaporin-1 OS=Homo sapiens OX=9606 GN=AQP1 PE=1 SV=3
ARF3	P61204	ADP-ribosylation factor 3 OS=Homo sapiens OX=9606 GN=ARF3 PE=1 SV=2
ARG1	P05089	Arginase-1 OS=Homo sapiens OX=9606 GN=ARG1 PE=1 SV=2
ARHGAP25	P42331	Rho GTPase-activating protein 25 OS=Homo sapiens OX=9606 GN=ARHGAP25 PE=1 SV=2
ARHGAP45	Q92619	Rho GTPase-activating protein 45 OS=Homo sapiens OX=9606 GN=ARHGAP45 PE=1 SV=2
ARHGDIA	P52565	Rho GDP-dissociation inhibitor 1 OS=Homo sapiens OX=9606 GN=ARHGDIA PE=1 SV=3
ARHGDIB	P52566	Rho GDP-dissociation inhibitor 2 OS=Homo sapiens OX=9606 GN=ARHGDIB PE=1 SV=3
ARL6IP5	O75915	PRA1 family protein 3 OS=Homo sapiens OX=9606 GN=ARL6IP5 PE=1 SV=1
ARPC1B	O15143	Actin-related protein 2/3 complex subunit 1B OS=Homo sapiens OX=9606 GN=ARPC1B PE=1 SV=3

Protein	Accession	Description
ARPC2	O15144	Actin-related protein 2/3 complex subunit 2 OS=Homo sapiens OX=9606 GN=ARPC2 PE=1 SV=1
ARPC3	O15145	Actin-related protein 2/3 complex subunit 3 OS=Homo sapiens OX=9606 GN=ARPC3 PE=1 SV=3
ARPC4	P59998	Actin-related protein 2/3 complex subunit 4 OS=Homo sapiens OX=9606 GN=ARPC4 PE=1 SV=3
ARPC5	O15511	Actin-related protein 2/3 complex subunit 5 OS=Homo sapiens OX=9606 GN=ARPC5 PE=1 SV=3
ATG3	Q9NT62	Ubiquitin-like-conjugating enzyme ATG3 OS=Homo sapiens OX=9606 GN=ATG3 PE=1 SV=1
ATIC	P31939	Bifunctional purine biosynthesis protein ATIC OS=Homo sapiens OX=9606 GN=ATIC PE=1 SV=3
ATP1A1	P05023	Sodium/potassium-transporting ATPase subunit alpha-1 OS=Homo sapiens OX=9606 GN=ATP1A1 PE=1 SV=1
ATP1B3	P54709	Sodium/potassium-transporting ATPase subunit beta-3 OS=Homo sapiens OX=9606 GN=ATP1B3 PE=1 SV=1
ATP2B4	P23634	Plasma membrane calcium-transporting ATPase 4 OS=Homo sapiens OX=9606 GN=ATP2B4 PE=1 SV=2
ATP5F1A	P25705	ATP synthase subunit alpha, mitochondrial OS=Homo sapiens OX=9606 GN=ATP5F1A PE=1 SV=1
ATP6V0A1	Q93050	V-type proton ATPase 116 kDa subunit a 1 OS=Homo sapiens OX=9606 GN=ATP6V0A1 PE=1 SV=3
ATP6V0D1	P61421	V-type proton ATPase subunit d 1 OS=Homo sapiens OX=9606 GN=ATP6V0D1 PE=1 SV=1
ATP6V1B2	P21281	V-type proton ATPase subunit B, brain isoform OS=Homo sapiens OX=9606 GN=ATP6V1B2 PE=1 SV=3
ATP6V1E1	P36543	V-type proton ATPase subunit E 1 OS=Homo sapiens OX=9606 GN=ATP6V1E1 PE=1 SV=1
ATP6V1G1	O75348	V-type proton ATPase subunit G 1 OS=Homo sapiens OX=9606 GN=ATP6V1G1 PE=1 SV=3
ATP7A	Q04656	Copper-transporting ATPase 1 OS=Homo sapiens OX=9606 GN=ATP7A PE=1 SV=4
AZU1	P20160	Azurocidin OS=Homo sapiens OX=9606 GN=AZU1 PE=1 SV=3
B2M	P61769	Beta-2-microglobulin OS=Homo sapiens OX=9606 GN=B2M PE=1 SV=1
BABAM1	Q9NWV8	BRISC and BRCA1-A complex member 1 OS=Homo sapiens OX=9606 GN=BABAM1 PE=1 SV=1
BANK1	Q8NDB2	B-cell scaffold protein with ankyrin repeats OS=Homo sapiens OX=9606 GN=BANK1 PE=1 SV=3
BASP1	P80723	Brain acid soluble protein 1 OS=Homo sapiens OX=9606 GN=BASP1 PE=1 SV=2
BCAM	P50895	Basal cell adhesion molecule OS=Homo sapiens OX=9606 GN=BCAM PE=1 SV=2
BCAP31	P51572	B-cell receptor-associated protein 31 OS=Homo sapiens OX=9606 GN=BCAP31 PE=1 SV=3
BID	P55957	BH3-interacting domain death agonist OS=Homo sapiens OX=9606 GN=BID PE=1 SV=1

Protein	Accession	Description
BIN2	Q9UBW5	Bridging integrator 2 OS=Homo sapiens OX=9606 GN=BIN2 PE=1 SV=3
BLVRB	P30043	Flavin reductase (NADPH) OS=Homo sapiens OX=9606 GN=BLVRB PE=1 SV=3
BOLA2B	Q9H3K6	BolA-like protein 2 OS=Homo sapiens OX=9606 GN=BOLA2B PE=1 SV=1
BPGM	P07738	Bisphosphoglycerate mutase OS=Homo sapiens OX=9606 GN=BPGM PE=1 SV=2
BPI	P17213	Bactericidal permeability-increasing protein OS=Homo sapiens OX=9606 GN=BPI PE=1 SV=4
BSG	P35613	Basigin OS=Homo sapiens OX=9606 GN=BSG PE=1 SV=2
BST1	Q10588	ADP-ribosyl cyclase/cyclic ADP-ribose hydrolase 2 OS=Homo sapiens OX=9606 GN=BST1 PE=1 SV=2
C1QB	P02746	Complement C1q subcomponent subunit B OS=Homo sapiens OX=9606 GN=C1QB PE=1 SV=3
C1QBP	Q07021	Complement component 1 Q subcomponent-binding protein, mitochondrial OS=Homo sapiens OX=9606 GN=C1QBP PE=1 SV=1
C1QC	P02747	Complement C1q subcomponent subunit C OS=Homo sapiens OX=9606 GN=C1QC PE=1 SV=3
C1R	P00736	Complement C1r subcomponent OS=Homo sapiens OX=9606 GN=C1R PE=1 SV=2
C1S	P09871	Complement C1s subcomponent OS=Homo sapiens OX=9606 GN=C1S PE=1 SV=1
C2	P06681	Complement C2 OS=Homo sapiens OX=9606 GN=C2 PE=1 SV=2
C3	P01024	Complement C3 OS=Homo sapiens OX=9606 GN=C3 PE=1 SV=2
C4A	P0C0L4	Complement C4-A OS=Homo sapiens OX=9606 GN=C4A PE=1 SV=2
C4B_2	P0C0L5	Complement C4-B OS=Homo sapiens OX=9606 GN=C4B_2 PE=1 SV=2
C4BPA	P04003	C4b-binding protein alpha chain OS=Homo sapiens OX=9606 GN=C4BPA PE=1 SV=2
C4BPB	P20851	C4b-binding protein beta chain OS=Homo sapiens OX=9606 GN=C4BPB PE=1 SV=1
C5AR1	P21730	C5a anaphylatoxin chemotactic receptor 1 OS=Homo sapiens OX=9606 GN=C5AR1 PE=1 SV=2
C6	P13671	Complement component C6 OS=Homo sapiens OX=9606 GN=C6 PE=1 SV=3
C7	P10643	Complement component C7 OS=Homo sapiens OX=9606 GN=C7 PE=1 SV=2
C8A	P07357	Complement component C8 alpha chain OS=Homo sapiens OX=9606 GN=C8A PE=1 SV=2
C8B	P07358	Complement component C8 beta chain OS=Homo sapiens OX=9606 GN=C8B PE=1 SV=3
C8G	P07360	Complement component C8 gamma chain OS=Homo sapiens OX=9606 GN=C8G PE=1 SV=3

Protein	Accession	Description
C9	P02748	Complement component C9 OS=Homo sapiens OX=9606 GN=C9 PE=1 SV=2
C9orf40	Q8IXQ3	Uncharacterized protein C9orf40 OS=Homo sapiens OX=9606 GN=C9orf40 PE=1 SV=1
CA1	P00915	Carbonic anhydrase 1 OS=Homo sapiens OX=9606 GN=CA1 PE=1 SV=2
CA2	P00918	Carbonic anhydrase 2 OS=Homo sapiens OX=9606 GN=CA2 PE=1 SV=2
CALD1	Q05682	Caldesmon OS=Homo sapiens OX=9606 GN=CALD1 PE=1 SV=3
CALM3	P0DP25	Calmodulin-3 OS=Homo sapiens OX=9606 GN=CALM3 PE=1 SV=1
CALR	P27797	Calreticulin OS=Homo sapiens OX=9606 GN=CALR PE=1 SV=1
CALU	O43852	Calumenin OS=Homo sapiens OX=9606 GN=CALU PE=1 SV=2
CAMP	P49913	Cathelicidin antimicrobial peptide OS=Homo sapiens OX=9606 GN=CAMP PE=1 SV=1
CAND1	Q86VP6	Cullin-associated NEDD8-dissociated protein 1 OS=Homo sapiens OX=9606 GN=CAND1 PE=1 SV=2
CANX	P27824	Calnexin OS=Homo sapiens OX=9606 GN=CANX PE=1 SV=2
CAP1	Q01518	Adenylyl cyclase-associated protein 1 OS=Homo sapiens OX=9606 GN=CAP1 PE=1 SV=5
CAPG	P40121	Macrophage-capping protein OS=Homo sapiens OX=9606 GN=CAPG PE=1 SV=2
CAPN1	P07384	Calpain-1 catalytic subunit OS=Homo sapiens OX=9606 GN=CAPN1 PE=1 SV=1
CAPNS1	P04632	Calpain small subunit 1 OS=Homo sapiens OX=9606 GN=CAPNS1 PE=1 SV=1
CAPRIN1	Q14444	Caprin-1 OS=Homo sapiens OX=9606 GN=CAPRIN1 PE=1 SV=2
CAPZA1	P52907	F-actin-capping protein subunit alpha-1 OS=Homo sapiens OX=9606 GN=CAPZA1 PE=1 SV=3
CAPZB	P47756	F-actin-capping protein subunit beta OS=Homo sapiens OX=9606 GN=CAPZB PE=1 SV=5
CARHSP1	Q9Y2V2	Calcium-regulated heat-stable protein 1 OS=Homo sapiens OX=9606 GN=CARHSP1 PE=1 SV=2
CASP14	P31944	Caspase-14 OS=Homo sapiens OX=9606 GN=CASP14 PE=1 SV=2
CASP3	P42574	Caspase-3 OS=Homo sapiens OX=9606 GN=CASP3 PE=1 SV=2
CAST	P20810	Calpastatin OS=Homo sapiens OX=9606 GN=CAST PE=1 SV=4
CAT	P04040	Catalase OS=Homo sapiens OX=9606 GN=CAT PE=1 SV=3
CAVIN2	O95810	Caveolae-associated protein 2 OS=Homo sapiens OX=9606 GN=CAVIN2 PE=1 SV=3
CBX1	P83916	Chromobox protein homolog 1 OS=Homo sapiens OX=9606 GN=CBX1 PE=1 SV=1

Protein	Accession	Description
CBX3	Q13185	Chromobox protein homolog 3 OS=Homo sapiens OX=9606 GN=CBX3 PE=1 SV=4
CCDC175	P0C221	Coiled-coil domain-containing protein 175 OS=Homo sapiens OX=9606 GN=CCDC175 PE=4 SV=2
CCDC88B	A6NC98	Coiled-coil domain-containing protein 88B OS=Homo sapiens OX=9606 GN=CCDC88B PE=1 SV=1
CCL14	Q16627	C-C motif chemokine 14 OS=Homo sapiens OX=9606 GN=CCL14 PE=1 SV=1
CCS	O14618	Copper chaperone for superoxide dismutase OS=Homo sapiens OX=9606 GN=CCS PE=1 SV=1
CCT2	P78371	T-complex protein 1 subunit beta OS=Homo sapiens OX=9606 GN=CCT2 PE=1 SV=4
CCT3	P49368	T-complex protein 1 subunit gamma OS=Homo sapiens OX=9606 GN=CCT3 PE=1 SV=4
CCT4	P50991	T-complex protein 1 subunit delta OS=Homo sapiens OX=9606 GN=CCT4 PE=1 SV=4
CCT5	P48643	T-complex protein 1 subunit epsilon OS=Homo sapiens OX=9606 GN=CCT5 PE=1 SV=1
CCT6A	P40227	T-complex protein 1 subunit zeta OS=Homo sapiens OX=9606 GN=CCT6A PE=1 SV=3
CCT8	P50990	T-complex protein 1 subunit theta OS=Homo sapiens OX=9606 GN=CCT8 PE=1 SV=4
CD14	P08571	Monocyte differentiation antigen CD14 OS=Homo sapiens OX=9606 GN=CD14 PE=1 SV=2
CD2	P06729	T-cell surface antigen CD2 OS=Homo sapiens OX=9606 GN=CD2 PE=1 SV=2
CD226	Q15762	CD226 antigen OS=Homo sapiens OX=9606 GN=CD226 PE=1 SV=2
CD248	Q9HCU0	Endosialin OS=Homo sapiens OX=9606 GN=CD248 PE=1 SV=1
CD2AP	Q9Y5K6	CD2-associated protein OS=Homo sapiens OX=9606 GN=CD2AP PE=1 SV=1
CD36	P16671	Platelet glycoprotein 4 OS=Homo sapiens OX=9606 GN=CD36 PE=1 SV=2
CD44	P16070	CD44 antigen OS=Homo sapiens OX=9606 GN=CD44 PE=1 SV=3
CD47	Q08722	Leukocyte surface antigen CD47 OS=Homo sapiens OX=9606 GN=CD47 PE=1 SV=1
CD53	P19397	Leukocyte surface antigen CD53 OS=Homo sapiens OX=9606 GN=CD53 PE=1 SV=1
CD58	P19256	Lymphocyte function-associated antigen 3 OS=Homo sapiens OX=9606 GN=CD58 PE=1 SV=1
CD59	P13987	CD59 glycoprotein OS=Homo sapiens OX=9606 GN=CD59 PE=1 SV=1
CD5L	O43866	CD5 antigen-like OS=Homo sapiens OX=9606 GN=CD5L PE=1 SV=1
CD9	P21926	CD9 antigen OS=Homo sapiens OX=9606 GN=CD9 PE=1 SV=4

Protein	Accession	Description
CD99	P14209	CD99 antigen OS=Homo sapiens OX=9606 GN=CD99 PE=1 SV=1
CDA	P32320	Cytidine deaminase OS=Homo sapiens OX=9606 GN=CDA PE=1 SV=2
CDC37	Q16543	Hsp90 co-chaperone Cdc37 OS=Homo sapiens OX=9606 GN=CDC37 PE=1 SV=1
CDC42	P60953	Cell division control protein 42 homolog OS=Homo sapiens OX=9606 GN=CDC42 PE=1 SV=2
CDK11B	P21127	Cyclin-dependent kinase 11B OS=Homo sapiens OX=9606 GN=CDK11B PE=1 SV=4
CDSN	Q15517	Corneodesmosin OS=Homo sapiens OX=9606 GN=CDSN PE=1 SV=3
CEP290	O15078	Centrosomal protein of 290 kDa OS=Homo sapiens OX=9606 GN=CEP290 PE=1 SV=2
CFB	P00751	Complement factor B OS=Homo sapiens OX=9606 GN=CFB PE=1 SV=2
CFH	P08603	Complement factor H OS=Homo sapiens OX=9606 GN=CFH PE=1 SV=4
CFHR1	Q03591	Complement factor H-related protein 1 OS=Homo sapiens OX=9606 GN=CFHR1 PE=1 SV=2
CFHR2	P36980	Complement factor H-related protein 2 OS=Homo sapiens OX=9606 GN=CFHR2 PE=1 SV=1
CFHR4	Q92496	Complement factor H-related protein 4 OS=Homo sapiens OX=9606 GN=CFHR4 PE=1 SV=3
CFI	P05156	Complement factor I OS=Homo sapiens OX=9606 GN=CFI PE=1 SV=2
CFL1	P23528	Cofilin-1 OS=Homo sapiens OX=9606 GN=CFL1 PE=1 SV=3
CHD5	Q8TDI0	Chromodomain-helicase-DNA-binding protein 5 OS=Homo sapiens OX=9606 GN=CHD5 PE=1 SV=1
CHGA	P10645	Chromogranin-A OS=Homo sapiens OX=9606 GN=CHGA PE=1 SV=7
CHMP1A	Q9HD42	Charged multivesicular body protein 1a OS=Homo sapiens OX=9606 GN=CHMP1A PE=1 SV=1
CHMP1B	Q7LBR1	Charged multivesicular body protein 1b OS=Homo sapiens OX=9606 GN=CHMP1B PE=1 SV=1
CHMP2A	O43633	Charged multivesicular body protein 2a OS=Homo sapiens OX=9606 GN=CHMP2A PE=1 SV=1
CHMP3	Q9Y3E7	Charged multivesicular body protein 3 OS=Homo sapiens OX=9606 GN=CHMP3 PE=1 SV=3
CHMP4A	Q9BY43	Charged multivesicular body protein 4a OS=Homo sapiens OX=9606 GN=CHMP4A PE=1 SV=3
CHMP4B	Q9H444	Charged multivesicular body protein 4b OS=Homo sapiens OX=9606 GN=CHMP4B PE=1 SV=1
CHMP5	Q9NZZ3	Charged multivesicular body protein 5 OS=Homo sapiens OX=9606 GN=CHMP5 PE=1 SV=1
CIAO2A	Q9H5X1	Cytosolic iron-sulfur assembly component 2A OS=Homo sapiens OX=9606 GN=CIAO2A PE=1 SV=1
CLC	Q05315	Galectin-10 OS=Homo sapiens OX=9606 GN=CLC PE=1 SV=3

Protein	Accession	Description
CLEC11A	Q9Y240	C-type lectin domain family 11 member A OS=Homo sapiens OX=9606 GN=CLEC11A PE=1 SV=1
CLEC3B	P05452	Tetranectin OS=Homo sapiens OX=9606 GN=CLEC3B PE=1 SV=3
CLIC1	O00299	Chloride intracellular channel protein 1 OS=Homo sapiens OX=9606 GN=CLIC1 PE=1 SV=4
CLINT1	Q14677	Clathrin interactor 1 OS=Homo sapiens OX=9606 GN=CLINT1 PE=1 SV=1
CLNS1A	P54105	Methylosome subunit pICln OS=Homo sapiens OX=9606 GN=CLNS1A PE=1 SV=1
CLTA	P09496	Clathrin light chain A OS=Homo sapiens OX=9606 GN=CLTA PE=1 SV=1
CLTB	P09497	Clathrin light chain B OS=Homo sapiens OX=9606 GN=CLTB PE=1 SV=1
CLTC	Q00610	Clathrin heavy chain 1 OS=Homo sapiens OX=9606 GN=CLTC PE=1 SV=5
CLU	P10909	Clusterin OS=Homo sapiens OX=9606 GN=CLU PE=1 SV=1
CMPK1	P30085	UMP-CMP kinase OS=Homo sapiens OX=9606 GN=CMPK1 PE=1 SV=3
CNN2	Q99439	Calponin-2 OS=Homo sapiens OX=9606 GN=CNN2 PE=1 SV=4
1 COP	Q8NHY2	E3 ubiquitin-protein ligase COP1 OS=Homo sapiens OX=9606 GN=COP1 PE=1 SV=1
COPG1	Q9Y678	Coatomer subunit gamma-1 OS=Homo sapiens OX=9606 GN=COPG1 PE=1 SV=1
COPS4	Q9BT78	COP9 signalosome complex subunit 4 OS=Homo sapiens OX=9606 GN=COPS4 PE=1 SV=1
COPS9	Q8WXC6	COP9 signalosome complex subunit 9 OS=Homo sapiens OX=9606 GN=COPS9 PE=1 SV=3
COPZ1	P61923	Coatomer subunit zeta-1 OS=Homo sapiens OX=9606 GN=COPZ1 PE=1 SV=1
CORO1A	P31146	Coronin-1A OS=Homo sapiens OX=9606 GN=CORO1A PE=1 SV=4
CORO1C	Q9ULV4	Coronin-1C OS=Homo sapiens OX=9606 GN=CORO1C PE=1 SV=1
COTL1	Q14019	Coactosin-like protein OS=Homo sapiens OX=9606 GN=COTL1 PE=1 SV=3
COX5B	P10606	Cytochrome c oxidase subunit 5B, mitochondrial OS=Homo sapiens OX=9606 GN=COX5B PE=1 SV=2
COX6B1	P14854	Cytochrome c oxidase subunit 6B1 OS=Homo sapiens OX=9606 GN=COX6B1 PE=1 SV=2
CP	P00450	Ceruloplasmin OS=Homo sapiens OX=9606 GN=CP PE=1 SV=1
CPN1	P15169	Carboxypeptidase N catalytic chain OS=Homo sapiens OX=9606 GN=CPN1 PE=1 SV=1
CPN2	P22792	Carboxypeptidase N subunit 2 OS=Homo sapiens OX=9606 GN=CPN2 PE=1 SV=3
CR1	P17927	Complement receptor type 1 OS=Homo sapiens OX=9606 GN=CR1 PE=1 SV=3

Protein	Accession	Description
CREB3L4	Q8TEY5	Cyclic AMP-responsive element-binding protein 3-like protein 4 OS=Homo sapiens OX=9606 GN=CREB3L4 PE=1 SV=1
CRKL	P46109	Crk-like protein OS=Homo sapiens OX=9606 GN=CRKL PE=1 SV=1
CRLF3	Q8IUI8	Cytokine receptor-like factor 3 OS=Homo sapiens OX=9606 GN=CRLF3 PE=1 SV=2
CSMD2	Q7Z408	CUB and sushi domain-containing protein 2 OS=Homo sapiens OX=9606 GN=CSMD2 PE=1 SV=2
CSNK2A1	P68400	Casein kinase II subunit alpha OS=Homo sapiens OX=9606 GN=CSNK2A1 PE=1 SV=1
CSNK2B	P67870	Casein kinase II subunit beta OS=Homo sapiens OX=9606 GN=CSNK2B PE=1 SV=1
CSRP1	P21291	Cysteine and glycine-rich protein 1 OS=Homo sapiens OX=9606 GN=CSRP1 PE=1 SV=3
CST3	P01034	Cystatin-C OS=Homo sapiens OX=9606 GN=CST3 PE=1 SV=1
CST7	O76096	Cystatin-F OS=Homo sapiens OX=9606 GN=CST7 PE=1 SV=1
CTSB	P07858	Cathepsin B OS=Homo sapiens OX=9606 GN=CTSB PE=1 SV=3
CTSG	P08311	Cathepsin G OS=Homo sapiens OX=9606 GN=CTSG PE=1 SV=2
CTTN	Q14247	Src substrate cortactin OS=Homo sapiens OX=9606 GN=CTTN PE=1 SV=2
CYBB	P04839	Cytochrome b-245 heavy chain OS=Homo sapiens OX=9606 GN=CYBB PE=1 SV=2
CYBRD1	Q53TN4	Plasma membrane ascorbate-dependent reductase CYBRD1 OS=Homo sapiens OX=9606 GN=CYBRD1 PE=1 SV=1
CYP26B1	Q9NR63	Cytochrome P450 26B1 OS=Homo sapiens OX=9606 GN=CYP26B1 PE=1 SV=1
DBN1	Q16643	Drebrin OS=Homo sapiens OX=9606 GN=DBN1 PE=1 SV=4
DBNL	Q9UJU6	Drebrin-like protein OS=Homo sapiens OX=9606 GN=DBNL PE=1 SV=1
DCD	P81605	Dermcidin OS=Homo sapiens OX=9606 GN=DCD PE=1 SV=2
DCTN1	Q14203	Dynactin subunit 1 OS=Homo sapiens OX=9606 GN=DCTN1 PE=1 SV=3
DCTN2	Q13561	Dynactin subunit 2 OS=Homo sapiens OX=9606 GN=DCTN2 PE=1 SV=4
DDB1	Q16531	DNA damage-binding protein 1 OS=Homo sapiens OX=9606 GN=DDB1 PE=1 SV=1
DDI2	Q5TDH0	Protein DDI1 homolog 2 OS=Homo sapiens OX=9606 GN=DDI2 PE=1 SV=1
DDX17	Q92841	Probable ATP-dependent RNA helicase DDX17 OS=Homo sapiens OX=9606 GN=DDX17 PE=1 SV=2
DDX21	Q9NR30	Nucleolar RNA helicase 2 OS=Homo sapiens OX=9606 GN=DDX21 PE=1 SV=5
DDX39B	Q13838	Spliceosome RNA helicase DDX39B OS=Homo sapiens OX=9606 GN=DDX39B PE=1 SV=1
DEFA1B	P59665	Neutrophil defensin 1 OS=Homo sapiens OX=9606 GN=DEFA1B PE=1 SV=1

Protein	Accession	Description
DIAPH1	O60610	Protein diaphanous homolog 1 OS=Homo sapiens OX=9606 GN=DIAPH1 PE=1 SV=2
DIRAS2	Q96HU8	GTP-binding protein Di-Ras2 OS=Homo sapiens OX=9606 GN=DIRAS2 PE=1 SV=1
DMTN	Q08495	Dematin OS=Homo sapiens OX=9606 GN=DMTN PE=1 SV=3
DNAJB2	P25686	DnaJ homolog subfamily B member 2 OS=Homo sapiens OX=9606 GN=DNAJB2 PE=1 SV=3
DNAJB4	Q9UDY4	DnaJ homolog subfamily B member 4 OS=Homo sapiens OX=9606 GN=DNAJB4 PE=1 SV=1
DTYMK	P23919	Thymidylate kinase OS=Homo sapiens OX=9606 GN=DTYMK PE=1 SV=4
ECM1	Q16610	Extracellular matrix protein 1 OS=Homo sapiens OX=9606 GN=ECM1 PE=1 SV=2
EEF1A1	P68104	Elongation factor 1-alpha 1 OS=Homo sapiens OX=9606 GN=EEF1A1 PE=1 SV=1
EEF1B2	P24534	Elongation factor 1-beta OS=Homo sapiens OX=9606 GN=EEF1B2 PE=1 SV=3
EEF1D	P29692	Elongation factor 1-delta OS=Homo sapiens OX=9606 GN=EEF1D PE=1 SV=5
EEF1G	P26641	Elongation factor 1-gamma OS=Homo sapiens OX=9606 GN=EEF1G PE=1 SV=3
EEF2	P13639	Elongation factor 2 OS=Homo sapiens OX=9606 GN=EEF2 PE=1 SV=4
EFHD2	Q96C19	EF-hand domain-containing protein D2 OS=Homo sapiens OX=9606 GN=EFHD2 PE=1 SV=1
EHD1	Q9H4M9	EH domain-containing protein 1 OS=Homo sapiens OX=9606 GN=EHD1 PE=1 SV=2
EIF1AY	O14602	Eukaryotic translation initiation factor 1A, Y-chromosomal OS=Homo sapiens OX=9606 GN=EIF1AY PE=1 SV=4
EIF2S2	P20042	Eukaryotic translation initiation factor 2 subunit 2 OS=Homo sapiens OX=9606 GN=EIF2S2 PE=1 SV=2
EIF3A	Q14152	Eukaryotic translation initiation factor 3 subunit A OS=Homo sapiens OX=9606 GN=EIF3A PE=1 SV=1
EIF3B	P55884	Eukaryotic translation initiation factor 3 subunit B OS=Homo sapiens OX=9606 GN=EIF3B PE=1 SV=3
EIF3D	O15371	Eukaryotic translation initiation factor 3 subunit D OS=Homo sapiens OX=9606 GN=EIF3D PE=1 SV=1
EIF3G	O75821	Eukaryotic translation initiation factor 3 subunit G OS=Homo sapiens OX=9606 GN=EIF3G PE=1 SV=2
EIF3J	O75822	Eukaryotic translation initiation factor 3 subunit J OS=Homo sapiens OX=9606 GN=EIF3J PE=1 SV=2
EIF4E	P06730	Eukaryotic translation initiation factor 4E OS=Homo sapiens OX=9606 GN=EIF4E PE=1 SV=2
EIF4G1	Q04637	Eukaryotic translation initiation factor 4 gamma 1 OS=Homo sapiens OX=9606 GN=EIF4G1 PE=1 SV=4
EIF5	P55010	Eukaryotic translation initiation factor 5 OS=Homo sapiens OX=9606 GN=EIF5 PE=1 SV=2
EIF5A	P63241	Eukaryotic translation initiation factor 5A-1 OS=Homo sapiens OX=9606 GN=EIF5A PE=1 SV=2

Protein	Accession	Description
ELANE	P08246	Neutrophil elastase OS=Homo sapiens OX=9606 GN=ELANE PE=1 SV=1
ELOC	Q15369	Elongin-C OS=Homo sapiens OX=9606 GN=ELOC PE=1 SV=1
EMILIN1	Q9Y6C2	EMILIN-1 OS=Homo sapiens OX=9606 GN=EMILIN1 PE=1 SV=3
EMILIN2	Q9BXX0	EMILIN-2 OS=Homo sapiens OX=9606 GN=EMILIN2 PE=1 SV=3
EML4	Q9HC35	Echinoderm microtubule-associated protein-like 4 OS=Homo sapiens OX=9606 GN=EML4 PE=1 SV=3
ENO1	P06733	Alpha-enolase OS=Homo sapiens OX=9606 GN=ENO1 PE=1 SV=2
ENO2	P09104	Gamma-enolase OS=Homo sapiens OX=9606 GN=ENO2 PE=1 SV=3
EPB41	P11171	Protein 4.1 OS=Homo sapiens OX=9606 GN=EPB41 PE=1 SV=4
EPB42	P16452	Protein 4.2 OS=Homo sapiens OX=9606 GN=EPB42 PE=1 SV=3
EPS15	P42566	Epidermal growth factor receptor substrate 15 OS=Homo sapiens OX=9606 GN=EPS15 PE=1 SV=2
EPX	P11678	Eosinophil peroxidase OS=Homo sapiens OX=9606 GN=EPX PE=1 SV=2
ERMAP	Q96PL5	Erythroid membrane-associated protein OS=Homo sapiens OX=9606 GN=ERMAP PE=1 SV=1
ERP29	P30040	Endoplasmic reticulum resident protein 29 OS=Homo sapiens OX=9606 GN=ERP29 PE=1 SV=4
EVI2B	P34910	Protein EVI2B OS=Homo sapiens OX=9606 GN=EVI2B PE=1 SV=2
EZR	P15311	Ezrin OS=Homo sapiens OX=9606 GN=EZR PE=1 SV=4
F10	P00742	Coagulation factor X OS=Homo sapiens OX=9606 GN=F10 PE=1 SV=2
F11R	Q9Y624	Junctional adhesion molecule A OS=Homo sapiens OX=9606 GN=F11R PE=1 SV=1
F12	P00748	Coagulation factor XII OS=Homo sapiens OX=9606 GN=F12 PE=1 SV=3
F13A1	P00488	Coagulation factor XIII A chain OS=Homo sapiens OX=9606 GN=F13A1 PE=1 SV=5
F13B	P05160	Coagulation factor XIII B chain OS=Homo sapiens OX=9606 GN=F13B PE=1 SV=3
F2	P00734	Prothrombin OS=Homo sapiens OX=9606 GN=F2 PE=1 SV=2
F5	P12259	Coagulation factor V OS=Homo sapiens OX=9606 GN=F5 PE=1 SV=4
F9	P00740	Coagulation factor IX OS=Homo sapiens OX=9606 GN=F9 PE=1 SV=2
FABP5	Q01469	Fatty acid-binding protein 5 OS=Homo sapiens OX=9606 GN=FABP5 PE=1 SV=3
FAM90A20P	A6NIJ5	Putative protein FAM90A20P OS=Homo sapiens OX=9606 GN=FAM90A20P PE=5 SV=1

Protein	Accession	Description
FAU	P62861	FAU ubiquitin-like and ribosomal protein S30 OS=Homo sapiens OX=9606 GN=FAU PE=1 SV=2
FBLN1	P23142	Fibulin-1 OS=Homo sapiens OX=9606 GN=FBLN1 PE=1 SV=4
FCER1G	P30273	High affinity immunoglobulin epsilon receptor subunit gamma OS=Homo sapiens OX=9606 GN=FCER1G PE=1 SV=1
FCGR3A	P08637	Low affinity immunoglobulin gamma Fc region receptor III-A OS=Homo sapiens OX=9606 GN=FCGR3A PE=1 SV=2
FCGR3B	O75015	Low affinity immunoglobulin gamma Fc region receptor III-B OS=Homo sapiens OX=9606 GN=FCGR3B PE=1 SV=2
FCN1	O00602	Ficolin-1 OS=Homo sapiens OX=9606 GN=FCN1 PE=1 SV=2
FERMT3	Q86UX7	Fermitin family homolog 3 OS=Homo sapiens OX=9606 GN=FERMT3 PE=1 SV=1
FETUB	Q9UGM5	Fetuin-B OS=Homo sapiens OX=9606 GN=FETUB PE=1 SV=2
FGA	P02671	Fibrinogen alpha chain OS=Homo sapiens OX=9606 GN=FGA PE=1 SV=2
FGB	P02675	Fibrinogen beta chain OS=Homo sapiens OX=9606 GN=FGB PE=1 SV=2
FGG	P02679	Fibrinogen gamma chain OS=Homo sapiens OX=9606 GN=FGG PE=1 SV=3
FKBP15	Q5T1M5	FK506-binding protein 15 OS=Homo sapiens OX=9606 GN=FKBP15 PE=1 SV=2
FKBP1A	P62942	Peptidyl-prolyl cis-trans isomerase FKBP1A OS=Homo sapiens OX=9606 GN=FKBP1A PE=1 SV=2
FLII	Q13045	Protein flightless-1 homolog OS=Homo sapiens OX=9606 GN=FLII PE=1 SV=2
FLNA	P21333	Filamin-A OS=Homo sapiens OX=9606 GN=FLNA PE=1 SV=4
FLOT1	O75955	Flotillin-1 OS=Homo sapiens OX=9606 GN=FLOT1 PE=1 SV=3
FLOT2	Q14254	Flotillin-2 OS=Homo sapiens OX=9606 GN=FLOT2 PE=1 SV=2
FN1	P02751	Fibronectin OS=Homo sapiens OX=9606 GN=FN1 PE=1 SV=5
FN3K	Q9H479	Fructosamine-3-kinase OS=Homo sapiens OX=9606 GN=FN3K PE=1 SV=1
FPR3	P25089	N-formyl peptide receptor 3 OS=Homo sapiens OX=9606 GN=FPR3 PE=2 SV=2
FSTL1	Q12841	Follistatin-related protein 1 OS=Homo sapiens OX=9606 GN=FSTL1 PE=1 SV=1
FYB1	O15117	FYN-binding protein 1 OS=Homo sapiens OX=9606 GN=FYB1 PE=1 SV=2
G6PD	P11413	Glucose-6-phosphate 1-dehydrogenase OS=Homo sapiens OX=9606 GN=G6PD PE=1 SV=4
GABRP	O00591	Gamma-aminobutyric acid receptor subunit pi OS=Homo sapiens OX=9606 GN=GABRP PE=2 SV=1
GANAB	Q14697	Neutral alpha-glucosidase AB OS=Homo sapiens OX=9606 GN=GANAB PE=1 SV=3
GAPDH	P04406	Glyceraldehyde-3-phosphate dehydrogenase OS=Homo sapiens OX=9606 GN=GAPDH PE=1 SV=3

Protein	Accession	Description
GC	P02774	Vitamin D-binding protein OS=Homo sapiens OX=9606 GN=GC PE=1 SV=2
GCA	P28676	Grancalcin OS=Homo sapiens OX=9606 GN=GCA PE=1 SV=2
GCLM	P48507	Glutamate--cysteine ligase regulatory subunit OS=Homo sapiens OX=9606 GN=GCLM PE=1 SV=1
GDI2	P50395	Rab GDP dissociation inhibitor beta OS=Homo sapiens OX=9606 GN=GDI2 PE=1 SV=2
GET3	O43681	ATPase GET3 OS=Homo sapiens OX=9606 GN=GET3 PE=1 SV=2
GFUS	Q13630	GDP-L-fucose synthase OS=Homo sapiens OX=9606 GN=GFUS PE=1 SV=1
GLIPR2	Q9H4G4	Golgi-associated plant pathogenesis-related protein 1 OS=Homo sapiens OX=9606 GN=GLIPR2 PE=1 SV=3
GLO1	Q04760	Lactoylglutathione lyase OS=Homo sapiens OX=9606 GN=GLO1 PE=1 SV=4
GLRX	P35754	Glutaredoxin-1 OS=Homo sapiens OX=9606 GN=GLRX PE=1 SV=2
GMFB	P60983	Glia maturation factor beta OS=Homo sapiens OX=9606 GN=GMFB PE=1 SV=2
GMFG	O60234	Glia maturation factor gamma OS=Homo sapiens OX=9606 GN=GMFG PE=1 SV=1
GNAI2	P04899	Guanine nucleotide-binding protein G(i) subunit alpha-2 OS=Homo sapiens OX=9606 GN=GNAI2 PE=1 SV=3
GNB2	P62879	Guanine nucleotide-binding protein G(I)/G(S)/G(T) subunit beta-2 OS=Homo sapiens OX=9606 GN=GNB2 PE=1 SV=3
GNG2	P59768	Guanine nucleotide-binding protein G(I)/G(S)/G(O) subunit gamma-2 OS=Homo sapiens OX=9606 GN=GNG2 PE=1 SV=2
GOLM1	Q8NBJ4	Golgi membrane protein 1 OS=Homo sapiens OX=9606 GN=GOLM1 PE=1 SV=1
GOLM2	Q6P4E1	Protein GOLM2 OS=Homo sapiens OX=9606 GN=GOLM2 PE=1 SV=2
GP1BA	P07359	Platelet glycoprotein Ib alpha chain OS=Homo sapiens OX=9606 GN=GP1BA PE=1 SV=2
GP1BB	P13224	Platelet glycoprotein Ib beta chain OS=Homo sapiens OX=9606 GN=GP1BB PE=1 SV=1
GP5	P40197	Platelet glycoprotein V OS=Homo sapiens OX=9606 GN=GP5 PE=1 SV=1
GP6	Q9HCN6	Platelet glycoprotein VI OS=Homo sapiens OX=9606 GN=GP6 PE=1 SV=4
GP9	P14770	Platelet glycoprotein IX OS=Homo sapiens OX=9606 GN=GP9 PE=1 SV=3
GPI	P06744	Glucose-6-phosphate isomerase OS=Homo sapiens OX=9606 GN=GPI PE=1 SV=4
GPLD1	P80108	Phosphatidylinositol-glycan-specific phospholipase D OS=Homo sapiens OX=9606 GN=GPLD1 PE=1 SV=3
GPX1	P07203	Glutathione peroxidase 1 OS=Homo sapiens OX=9606 GN=GPX1 PE=1 SV=4
GRB2	P62993	Growth factor receptor-bound protein 2 OS=Homo sapiens OX=9606 GN=GRB2 PE=1 SV=1

Protein	Accession	Description
GRN	P28799	Progranulin OS=Homo sapiens OX=9606 GN=GRN PE=1 SV=2
GSN	P06396	Gelsolin OS=Homo sapiens OX=9606 GN=GSN PE=1 SV=1
GSTO1	P78417	Glutathione S-transferase omega-1 OS=Homo sapiens OX=9606 GN=GSTO1 PE=1 SV=2
GSTP1	P09211	Glutathione S-transferase P OS=Homo sapiens OX=9606 GN=GSTP1 PE=1 SV=2
GYG1	P46976	Glycogenin-1 OS=Homo sapiens OX=9606 GN=GYG1 PE=1 SV=4
H1-0	P07305	Histone H1.0 OS=Homo sapiens OX=9606 GN=H1-0 PE=1 SV=3
H1-10	Q92522	Histone H1.10 OS=Homo sapiens OX=9606 GN=H1-10 PE=1 SV=1
H1-3	P16402	Histone H1.3 OS=Homo sapiens OX=9606 GN=H1-3 PE=1 SV=2
H1-5	P16401	Histone H1.5 OS=Homo sapiens OX=9606 GN=H1-5 PE=1 SV=3
H2AC21	Q8IUE6	Histone H2A type 2-B OS=Homo sapiens OX=9606 GN=H2AC21 PE=1 SV=3
H2AC7	P20671	Histone H2A type 1-D OS=Homo sapiens OX=9606 GN=H2AC7 PE=1 SV=2
H2AC8	P04908	Histone H2A type 1-B/E OS=Homo sapiens OX=9606 GN=H2AC8 PE=1 SV=2
H2AX	P16104	Histone H2AX OS=Homo sapiens OX=9606 GN=H2AX PE=1 SV=2
H2AZ1	P0C0S5	Histone H2A.Z OS=Homo sapiens OX=9606 GN=H2AZ1 PE=1 SV=2
H2BC12L	P57053	Histone H2B type F-S OS=Homo sapiens OX=9606 GN=H2BC12L PE=1 SV=2
H2BC14	Q99879	Histone H2B type 1-M OS=Homo sapiens OX=9606 GN=H2BC14 PE=1 SV=3
H2BC3	P33778	Histone H2B type 1-B OS=Homo sapiens OX=9606 GN=H2BC3 PE=1 SV=2
H2BC5	P58876	Histone H2B type 1-D OS=Homo sapiens OX=9606 GN=H2BC5 PE=1 SV=2
H3-3B	P84243	Histone H3.3 OS=Homo sapiens OX=9606 GN=H3-3B PE=1 SV=2
H3-7	Q5TEC6	Histone H3-7 OS=Homo sapiens OX=9606 GN=H3-7 PE=1 SV=1
H3Y2	P0DPK5	Histone H3.X OS=Homo sapiens OX=9606 GN=H3Y2 PE=5 SV=1
H4C16	P62805	Histone H4 OS=Homo sapiens OX=9606 GN=H4C16 PE=1 SV=2
HABP2	Q14520	Hyaluronan-binding protein 2 OS=Homo sapiens OX=9606 GN=HABP2 PE=1 SV=1
HBA2	P69905	Hemoglobin subunit alpha OS=Homo sapiens OX=9606 GN=HBA2 PE=1 SV=2
HBB	P68871	Hemoglobin subunit beta OS=Homo sapiens OX=9606 GN=HBB PE=1 SV=2

Protein	Accession	Description
HBD	P02042	Hemoglobin subunit delta OS=Homo sapiens OX=9606 GN=HBD PE=1 SV=2
HBG1	P69891	Hemoglobin subunit gamma-1 OS=Homo sapiens OX=9606 GN=HBG1 PE=1 SV=2
HBG2	P69892	Hemoglobin subunit gamma-2 OS=Homo sapiens OX=9606 GN=HBG2 PE=1 SV=2
HBM	Q6B0K9	Hemoglobin subunit mu OS=Homo sapiens OX=9606 GN=HBM PE=1 SV=1
HBQ1	P09105	Hemoglobin subunit theta-1 OS=Homo sapiens OX=9606 GN=HBQ1 PE=1 SV=2
HCLS1	P14317	Hematopoietic lineage cell-specific protein OS=Homo sapiens OX=9606 GN=HCLS1 PE=1 SV=3
HDGF	P51858	Hepatoma-derived growth factor OS=Homo sapiens OX=9606 GN=HDGF PE=1 SV=1
HEBP1	Q9NRV9	Heme-binding protein 1 OS=Homo sapiens OX=9606 GN=HEBP1 PE=1 SV=1
HEG1	Q9ULI3	Protein HEG homolog 1 OS=Homo sapiens OX=9606 GN=HEG1 PE=1 SV=3
HGS	O14964	Hepatocyte growth factor-regulated tyrosine kinase substrate OS=Homo sapiens OX=9606 GN=HGS PE=1 SV=1
HINT1	P49773	Adenosine 5'-monophosphoramidase HINT1 OS=Homo sapiens OX=9606 GN=HINT1 PE=1 SV=2
HLA-A	P04439	HLA class I histocompatibility antigen, A alpha chain OS=Homo sapiens OX=9606 GN=HLA-A PE=1 SV=2
HLA-B	P01889	HLA class I histocompatibility antigen, B alpha chain OS=Homo sapiens OX=9606 GN=HLA-B PE=1 SV=3
HLA-C	P10321	HLA class I histocompatibility antigen, C alpha chain OS=Homo sapiens OX=9606 GN=HLA-C PE=1 SV=3
HMBS	P08397	Porphobilinogen deaminase OS=Homo sapiens OX=9606 GN=HMBS PE=1 SV=2
HMGB1	P09429	High mobility group protein B1 OS=Homo sapiens OX=9606 GN=HMGB1 PE=1 SV=3
HMGB2	P26583	High mobility group protein B2 OS=Homo sapiens OX=9606 GN=HMGB2 PE=1 SV=2
HMGB3	O15347	High mobility group protein B3 OS=Homo sapiens OX=9606 GN=HMGB3 PE=1 SV=4
HMGN2	P05204	Non-histone chromosomal protein HMG-17 OS=Homo sapiens OX=9606 GN=HMGN2 PE=1 SV=3
HMGN5	P82970	High mobility group nucleosome-binding domain-containing protein 5 OS=Homo sapiens OX=9606 GN=HMGN5 PE=1 SV=1
HNRNPA1	P09651	Heterogeneous nuclear ribonucleoprotein A1 OS=Homo sapiens OX=9606 GN=HNRNPA1 PE=1 SV=5
HNRNPA2B1	P22626	Heterogeneous nuclear ribonucleoproteins A2/B1 OS=Homo sapiens OX=9606 GN=HNRNPA2B1 PE=1 SV=2
HNRNPC	P07910	Heterogeneous nuclear ribonucleoproteins C1/C2 OS=Homo sapiens OX=9606 GN=HNRNPC PE=1 SV=4
HNRNPD	Q14103	Heterogeneous nuclear ribonucleoprotein D0 OS=Homo sapiens OX=9606 GN=HNRNPD PE=1 SV=1

Protein	Accession	Description
HNRNPF	P52597	Heterogeneous nuclear ribonucleoprotein F OS=Homo sapiens OX=9606 GN=HNRNPF PE=1 SV=3
HNRNPK	P61978	Heterogeneous nuclear ribonucleoprotein K OS=Homo sapiens OX=9606 GN=HNRNPK PE=1 SV=1
HNRNPL	P14866	Heterogeneous nuclear ribonucleoprotein L OS=Homo sapiens OX=9606 GN=HNRNPL PE=1 SV=2
HNRNPM	P52272	Heterogeneous nuclear ribonucleoprotein M OS=Homo sapiens OX=9606 GN=HNRNPM PE=1 SV=3
HNRNPU	Q00839	Heterogeneous nuclear ribonucleoprotein U OS=Homo sapiens OX=9606 GN=HNRNPU PE=1 SV=6
HNRNPUL2	Q1KMD3	Heterogeneous nuclear ribonucleoprotein U-like protein 2 OS=Homo sapiens OX=9606 GN=HNRNPUL2 PE=1 SV=1
HP	P00738	Haptoglobin OS=Homo sapiens OX=9606 GN=HP PE=1 SV=1
HP1BP3	Q5SSJ5	Heterochromatin protein 1-binding protein 3 OS=Homo sapiens OX=9606 GN=HP1BP3 PE=1 SV=1
HPR	P00739	Haptoglobin-related protein OS=Homo sapiens OX=9606 GN=HPR PE=2 SV=2
HPRT1	P00492	Hypoxanthine-guanine phosphoribosyltransferase OS=Homo sapiens OX=9606 GN=HPRT1 PE=1 SV=2
HPX	P02790	Hemopexin OS=Homo sapiens OX=9606 GN=HPX PE=1 SV=2
HRG	P04196	Histidine-rich glycoprotein OS=Homo sapiens OX=9606 GN=HRG PE=1 SV=1
HSP90AA1	P07900	Heat shock protein HSP 90-alpha OS=Homo sapiens OX=9606 GN=HSP90AA1 PE=1 SV=5
HSP90AB1	P08238	Heat shock protein HSP 90-beta OS=Homo sapiens OX=9606 GN=HSP90AB1 PE=1 SV=4
HSP90AB4P	Q58FF6	Putative heat shock protein HSP 90-beta 4 OS=Homo sapiens OX=9606 GN=HSP90AB4P PE=5 SV=1
HSP90B1	P14625	Endoplasmin OS=Homo sapiens OX=9606 GN=HSP90B1 PE=1 SV=1
HSPA1B	P0DMV9	Heat shock 70 kDa protein 1B OS=Homo sapiens OX=9606 GN=HSPA1B PE=1 SV=1
HSPA4	P34932	Heat shock 70 kDa protein 4 OS=Homo sapiens OX=9606 GN=HSPA4 PE=1 SV=4
HSPA5	P11021	Endoplasmic reticulum chaperone BiP OS=Homo sapiens OX=9606 GN=HSPA5 PE=1 SV=2
HSPA8	P11142	Heat shock cognate 71 kDa protein OS=Homo sapiens OX=9606 GN=HSPA8 PE=1 SV=1
HSPB1	P04792	Heat shock protein beta-1 OS=Homo sapiens OX=9606 GN=HSPB1 PE=1 SV=2
HSPD1	P10809	60 kDa heat shock protein, mitochondrial OS=Homo sapiens OX=9606 GN=HSPD1 PE=1 SV=2
HSPE1	P61604	10 kDa heat shock protein, mitochondrial OS=Homo sapiens OX=9606 GN=HSPE1 PE=1 SV=2
ICAM3	P32942	Intercellular adhesion molecule 3 OS=Homo sapiens OX=9606 GN=ICAM3 PE=1 SV=2
ICAM4	Q14773	Intercellular adhesion molecule 4 OS=Homo sapiens OX=9606 GN=ICAM4 PE=1 SV=1

Protein	Accession	Description
IGBP1	P78318	Immunoglobulin-binding protein 1 OS=Homo sapiens OX=9606 GN=IGBP1 PE=1 SV=1
IGHA1	P01876	Immunoglobulin heavy constant alpha 1 OS=Homo sapiens OX=9606 GN=IGHA1 PE=1 SV=2
IGHG2	P01859	Immunoglobulin heavy constant gamma 2 OS=Homo sapiens OX=9606 GN=IGHG2 PE=1 SV=2
IGHG3	P01860	Immunoglobulin heavy constant gamma 3 OS=Homo sapiens OX=9606 GN=IGHG3 PE=1 SV=2
IGHG4	P01861	Immunoglobulin heavy constant gamma 4 OS=Homo sapiens OX=9606 GN=IGHG4 PE=1 SV=1
IGHM	P01871	Immunoglobulin heavy constant mu OS=Homo sapiens OX=9606 GN=IGHM PE=1 SV=4
IGKC	P01834	Immunoglobulin kappa constant OS=Homo sapiens OX=9606 GN=IGKC PE=1 SV=2
IGKV2-40	A0A087WW87	Immunoglobulin kappa variable 2-40 OS=Homo sapiens OX=9606 GN=IGKV2-40 PE=3 SV=2
IGKV3-20	P01619	Immunoglobulin kappa variable 3-20 OS=Homo sapiens OX=9606 GN=IGKV3-20 PE=1 SV=2
IGKV3D-11	A0A0A0MRZ8	Immunoglobulin kappa variable 3D-11 OS=Homo sapiens OX=9606 GN=IGKV3D-11 PE=3 SV=6
IGKV3D-7	A0A0C4DH55	Immunoglobulin kappa variable 3D-7 OS=Homo sapiens OX=9606 GN=IGKV3D-7 PE=3 SV=5
IGLC2	P0DOY2	Immunoglobulin lambda constant 2 OS=Homo sapiens OX=9606 GN=IGLC2 PE=1 SV=1
IGLV1-47	P01700	Immunoglobulin lambda variable 1-47 OS=Homo sapiens OX=9606 GN=IGLV1-47 PE=1 SV=2
IGLV3-21	P80748	Immunoglobulin lambda variable 3-21 OS=Homo sapiens OX=9606 GN=IGLV3-21 PE=1 SV=2
IGLV3-25	P01717	Immunoglobulin lambda variable 3-25 OS=Homo sapiens OX=9606 GN=IGLV3-25 PE=1 SV=2
IL16	Q14005	Pro-interleukin-16 OS=Homo sapiens OX=9606 GN=IL16 PE=1 SV=4
ILK	Q13418	Integrin-linked protein kinase OS=Homo sapiens OX=9606 GN=ILK PE=1 SV=2
INSR	P06213	Insulin receptor OS=Homo sapiens OX=9606 GN=INSR PE=1 SV=4
IPO7	O95373	Importin-7 OS=Homo sapiens OX=9606 GN=IPO7 PE=1 SV=1
IQGAP1	P46940	Ras GTPase-activating-like protein IQGAP1 OS=Homo sapiens OX=9606 GN=IQGAP1 PE=1 SV=1
IQGAP2	Q13576	Ras GTPase-activating-like protein IQGAP2 OS=Homo sapiens OX=9606 GN=IQGAP2 PE=1 SV=4
IRGQ	Q8WZA9	Immunity-related GTPase family Q protein OS=Homo sapiens OX=9606 GN=IRGQ PE=1 SV=1
IST1	P53990	IST1 homolog OS=Homo sapiens OX=9606 GN=IST1 PE=1 SV=1
ITGA2B	P08514	Integrin alpha-IIb OS=Homo sapiens OX=9606 GN=ITGA2B PE=1 SV=3
ITGA6	P23229	Integrin alpha-6 OS=Homo sapiens OX=9606 GN=ITGA6 PE=1 SV=5

Protein	Accession	Description
ITGAM	P11215	Integrin alpha-M OS=Homo sapiens OX=9606 GN=ITGAM PE=1 SV=2
ITGB1	P05556	Integrin beta-1 OS=Homo sapiens OX=9606 GN=ITGB1 PE=1 SV=2
ITGB2	P05107	Integrin beta-2 OS=Homo sapiens OX=9606 GN=ITGB2 PE=1 SV=2
ITGB3	P05106	Integrin beta-3 OS=Homo sapiens OX=9606 GN=ITGB3 PE=1 SV=2
ITIH1	P19827	Inter-alpha-trypsin inhibitor heavy chain H1 OS=Homo sapiens OX=9606 GN=ITIH1 PE=1 SV=3
ITIH2	P19823	Inter-alpha-trypsin inhibitor heavy chain H2 OS=Homo sapiens OX=9606 GN=ITIH2 PE=1 SV=2
ITIH3	Q06033	Inter-alpha-trypsin inhibitor heavy chain H3 OS=Homo sapiens OX=9606 GN=ITIH3 PE=1 SV=2
ITIH4	Q14624	Inter-alpha-trypsin inhibitor heavy chain H4 OS=Homo sapiens OX=9606 GN=ITIH4 PE=1 SV=4
ITM2B	Q9Y287	Integral membrane protein 2B OS=Homo sapiens OX=9606 GN=ITM2B PE=1 SV=1
JCHAIN	P01591	Immunoglobulin J chain OS=Homo sapiens OX=9606 GN=JCHAIN PE=1 SV=4
JMJD4	Q9H9V9	2-oxoglutarate and iron-dependent oxygenase JMJD4 OS=Homo sapiens OX=9606 GN=JMJD4 PE=1 SV=2
KCNB2	Q92953	Potassium voltage-gated channel subfamily B member 2 OS=Homo sapiens OX=9606 GN=KCNB2 PE=2 SV=2
KEL	P23276	Kell blood group glycoprotein OS=Homo sapiens OX=9606 GN=KEL PE=1 SV=2
KHDRBS1	Q07666	KH domain-containing, RNA-binding, signal transduction-associated protein 1 OS=Homo sapiens OX=9606 GN=KHDRBS1 PE=1 SV=1
KIF2A	O00139	Kinesin-like protein KIF2A OS=Homo sapiens OX=9606 GN=KIF2A PE=1 SV=3
KIF5B	P33176	Kinesin-1 heavy chain OS=Homo sapiens OX=9606 GN=KIF5B PE=1 SV=1
KLKB1	P03952	Plasma kallikrein OS=Homo sapiens OX=9606 GN=KLKB1 PE=1 SV=1
KNG1	P01042	Kininogen-1 OS=Homo sapiens OX=9606 GN=KNG1 PE=1 SV=2
KPNB1	Q14974	Importin subunit beta-1 OS=Homo sapiens OX=9606 GN=KPNB1 PE=1 SV=2
KRT1	P04264	Keratin, type II cytoskeletal 1 OS=Homo sapiens OX=9606 GN=KRT1 PE=1 SV=6
KRT9	P35527	Keratin, type I cytoskeletal 9 OS=Homo sapiens OX=9606 GN=KRT9 PE=1 SV=3
LAMTOR1	Q6IAA8	Ragulator complex protein LAMTOR1 OS=Homo sapiens OX=9606 GN=LAMTOR1 PE=1 SV=2
LAMTOR5	O43504	Ragulator complex protein LAMTOR5 OS=Homo sapiens OX=9606 GN=LAMTOR5 PE=1 SV=1
LASP1	Q14847	LIM and SH3 domain protein 1 OS=Homo sapiens OX=9606 GN=LASP1 PE=1 SV=2

Protein	Accession	Description
LAT	O43561	Linker for activation of T-cells family member 1 OS=Homo sapiens OX=9606 GN=LAT PE=1 SV=1
LCAT	P04180	Phosphatidylcholine-sterol acyltransferase OS=Homo sapiens OX=9606 GN=LCAT PE=1 SV=1
LCN1	P31025	Lipocalin-1 OS=Homo sapiens OX=9606 GN=LCN1 PE=1 SV=1
LCN2	P80188	Neutrophil gelatinase-associated lipocalin OS=Homo sapiens OX=9606 GN=LCN2 PE=1 SV=2
LCP1	P13796	Plastin-2 OS=Homo sapiens OX=9606 GN=LCP1 PE=1 SV=6
LCP2	Q13094	Lymphocyte cytosolic protein 2 OS=Homo sapiens OX=9606 GN=LCP2 PE=1 SV=1
LDHA	P00338	L-lactate dehydrogenase A chain OS=Homo sapiens OX=9606 GN=LDHA PE=1 SV=2
LDHB	P07195	L-lactate dehydrogenase B chain OS=Homo sapiens OX=9606 GN=LDHB PE=1 SV=2
LGALS1	P09382	Galectin-1 OS=Homo sapiens OX=9606 GN=LGALS1 PE=1 SV=2
LGALS3	P17931	Galectin-3 OS=Homo sapiens OX=9606 GN=LGALS3 PE=1 SV=5
LGALS3BP	Q08380	Galectin-3-binding protein OS=Homo sapiens OX=9606 GN=LGALS3BP PE=1 SV=1
LIMS1	P48059	LIM and senescent cell antigen-like-containing domain protein 1 OS=Homo sapiens OX=9606 GN=LIMS1 PE=1 SV=4
LMNA	P02545	Prelamin-A/C OS=Homo sapiens OX=9606 GN=LMNA PE=1 SV=1
LMNB1	P20700	Lamin-B1 OS=Homo sapiens OX=9606 GN=LMNB1 PE=1 SV=2
LMNB2	Q03252	Lamin-B2 OS=Homo sapiens OX=9606 GN=LMNB2 PE=1 SV=4
LPA	P08519	Apolipoprotein(a) OS=Homo sapiens OX=9606 GN=LPA PE=1 SV=2
LRBA	P50851	Lipopolysaccharide-responsive and beige-like anchor protein OS=Homo sapiens OX=9606 GN=LRBA PE=1 SV=4
LRRFIP1	Q32MZ4	Leucine-rich repeat flightless-interacting protein 1 OS=Homo sapiens OX=9606 GN=LRRFIP1 PE=1 SV=2
7,00 LSM	Q9UK45	U6 snRNA-associated Sm-like protein LSm7 OS=Homo sapiens OX=9606 GN=LSM7 PE=1 SV=1
8,00 LSM	O95777	U6 snRNA-associated Sm-like protein LSm8 OS=Homo sapiens OX=9606 GN=LSM8 PE=1 SV=3
LSP1	P33241	Lymphocyte-specific protein 1 OS=Homo sapiens OX=9606 GN=LSP1 PE=1 SV=1
LTBP1	Q14766	Latent-transforming growth factor beta-binding protein 1 OS=Homo sapiens OX=9606 GN=LTBP1 PE=1 SV=4
LTF	P02788	Lactotransferrin OS=Homo sapiens OX=9606 GN=LTF PE=1 SV=6
LUM	P51884	Lumican OS=Homo sapiens OX=9606 GN=LUM PE=1 SV=2
LYPLA1	O75608	Acyl-protein thioesterase 1 OS=Homo sapiens OX=9606 GN=LYPLA1 PE=1 SV=1

Protein	Accession	Description
LYZ	P61626	Lysozyme C OS=Homo sapiens OX=9606 GN=LYZ PE=1 SV=1
LZIC	Q8WZA0	Protein LZIC OS=Homo sapiens OX=9606 GN=LZIC PE=1 SV=1
M6PR	P20645	Cation-dependent mannose-6-phosphate receptor OS=Homo sapiens OX=9606 GN=M6PR PE=1 SV=1
MACROH2A1	O75367	Core histone macro-H2A.1 OS=Homo sapiens OX=9606 GN=MACROH2A1 PE=1 SV=5
MAPK1	P28482	Mitogen-activated protein kinase 1 OS=Homo sapiens OX=9606 GN=MAPK1 PE=1 SV=3
MAPRE1	Q15691	Microtubule-associated protein RP/EB family member 1 OS=Homo sapiens OX=9606 GN=MAPRE1 PE=1 SV=3
MAPRE2	Q15555	Microtubule-associated protein RP/EB family member 2 OS=Homo sapiens OX=9606 GN=MAPRE2 PE=1 SV=1
MARCHF8	Q5T0T0	E3 ubiquitin-protein ligase MARCHF8 OS=Homo sapiens OX=9606 GN=MARCHF8 PE=1 SV=1
MARCKS	P29966	Myristoylated alanine-rich C-kinase substrate OS=Homo sapiens OX=9606 GN=MARCKS PE=1 SV=4
MASP1	P48740	Mannan-binding lectin serine protease 1 OS=Homo sapiens OX=9606 GN=MASP1 PE=1 SV=3
MDH1	P40925	Malate dehydrogenase, cytoplasmic OS=Homo sapiens OX=9606 GN=MDH1 PE=1 SV=4
MIF	P14174	Macrophage migration inhibitory factor OS=Homo sapiens OX=9606 GN=MIF PE=1 SV=4
MMP8	P22894	Neutrophil collagenase OS=Homo sapiens OX=9606 GN=MMP8 PE=1 SV=1
MMP9	P14780	Matrix metalloproteinase-9 OS=Homo sapiens OX=9606 GN=MMP9 PE=1 SV=3
MMRN1	Q13201	Multimerin-1 OS=Homo sapiens OX=9606 GN=MMRN1 PE=1 SV=3
MNDA	P41218	Myeloid cell nuclear differentiation antigen OS=Homo sapiens OX=9606 GN=MNDA PE=1 SV=1
MOB1B	Q7L9L4	MOB kinase activator 1B OS=Homo sapiens OX=9606 GN=MOB1B PE=1 SV=3
MPIG6B	O95866	Megakaryocyte and platelet inhibitory receptor G6b OS=Homo sapiens OX=9606 GN=MPIG6B PE=1 SV=1
MPO	P05164	Myeloperoxidase OS=Homo sapiens OX=9606 GN=MPO PE=1 SV=1
MPST	P25325	3-mercaptopyruvate sulfurtransferase OS=Homo sapiens OX=9606 GN=MPST PE=1 SV=3
MSN	P26038	Moesin OS=Homo sapiens OX=9606 GN=MSN PE=1 SV=3
MTHFD1	P11586	C-1-tetrahydrofolate synthase, cytoplasmic OS=Homo sapiens OX=9606 GN=MTHFD1 PE=1 SV=4
MTPN	P58546	Myotrophin OS=Homo sapiens OX=9606 GN=MTPN PE=1 SV=2
MVP	Q14764	Major vault protein OS=Homo sapiens OX=9606 GN=MVP PE=1 SV=4
MYH9	P35579	Myosin-9 OS=Homo sapiens OX=9606 GN=MYH9 PE=1 SV=4

Protein	Accession	Description
MYL12B	O14950	Myosin regulatory light chain 12B OS=Homo sapiens OX=9606 GN=MYL12B PE=1 SV=2
MYL4	P12829	Myosin light chain 4 OS=Homo sapiens OX=9606 GN=MYL4 PE=1 SV=3
MYL6	P60660	Myosin light polypeptide 6 OS=Homo sapiens OX=9606 GN=MYL6 PE=1 SV=2
MYL9	P24844	Myosin regulatory light polypeptide 9 OS=Homo sapiens OX=9606 GN=MYL9 PE=1 SV=4
MYLK	Q15746	Myosin light chain kinase, smooth muscle OS=Homo sapiens OX=9606 GN=MYLK PE=1 SV=4
MYO18A	Q92614	Unconventional myosin-XVIIIa OS=Homo sapiens OX=9606 GN=MYO18A PE=1 SV=3
NAA80	Q93015	N-alpha-acetyltransferase 80 OS=Homo sapiens OX=9606 GN=NAA80 PE=1 SV=2
NACA	E9PAV3	Nascent polypeptide-associated complex subunit alpha, muscle-specific form OS=Homo sapiens OX=9606 GN=NACA PE=1 SV=1
NACA2	Q9H009	Nascent polypeptide-associated complex subunit alpha-2 OS=Homo sapiens OX=9606 GN=NACA2 PE=1 SV=1
NAGK	Q9UJ70	N-acetyl-D-glucosamine kinase OS=Homo sapiens OX=9606 GN=NAGK PE=1 SV=4
NAMPT	P43490	Nicotinamide phosphoribosyltransferase OS=Homo sapiens OX=9606 GN=NAMPT PE=1 SV=1
NAP1L1	P55209	Nucleosome assembly protein 1-like 1 OS=Homo sapiens OX=9606 GN=NAP1L1 PE=1 SV=1
NAP1L4	Q99733	Nucleosome assembly protein 1-like 4 OS=Homo sapiens OX=9606 GN=NAP1L4 PE=1 SV=1
NAPA	P54920	Alpha-soluble NSF attachment protein OS=Homo sapiens OX=9606 GN=NAPA PE=1 SV=3
NAPRT	Q6XQN6	Nicotinate phosphoribosyltransferase OS=Homo sapiens OX=9606 GN=NAPRT PE=1 SV=2
NAT10	Q9H0A0	RNA cytidine acetyltransferase OS=Homo sapiens OX=9606 GN=NAT10 PE=1 SV=2
NCAM1	P13591	Neural cell adhesion molecule 1 OS=Homo sapiens OX=9606 GN=NCAM1 PE=1 SV=3
NCF1B	A6NI72	Putative neutrophil cytosol factor 1B OS=Homo sapiens OX=9606 GN=NCF1B PE=5 SV=2
NCF2	P19878	Neutrophil cytosol factor 2 OS=Homo sapiens OX=9606 GN=NCF2 PE=1 SV=2
NCL	P19338	Nucleolin OS=Homo sapiens OX=9606 GN=NCL PE=1 SV=3
NDEL1	Q9GZM8	Nuclear distribution protein nudE-like 1 OS=Homo sapiens OX=9606 GN=NDEL1 PE=1 SV=1
NECTIN1	Q15223	Nectin-1 OS=Homo sapiens OX=9606 GN=NECTIN1 PE=1 SV=3
NEDD8	Q15843	NEDD8 OS=Homo sapiens OX=9606 GN=NEDD8 PE=1 SV=1
NIBAN1	Q9BZQ8	Protein Niban 1 OS=Homo sapiens OX=9606 GN=NIBAN1 PE=1 SV=1
NID1	P14543	Nidogen-1 OS=Homo sapiens OX=9606 GN=NID1 PE=1 SV=3

Protein	Accession	Description
NID2	Q14112	Nidogen-2 OS=Homo sapiens OX=9606 GN=NID2 PE=1 SV=3
NME2	P22392	Nucleoside diphosphate kinase B OS=Homo sapiens OX=9606 GN=NME2 PE=1 SV=1
NPM1	P06748	Nucleophosmin OS=Homo sapiens OX=9606 GN=NPM1 PE=1 SV=2
NRAS	P01111	GTPase NRas OS=Homo sapiens OX=9606 GN=NRAS PE=1 SV=1
NSFL1C	Q9UNZ2	NSFL1 cofactor p47 OS=Homo sapiens OX=9606 GN=NSFL1C PE=1 SV=2
NUCB2	P80303	Nucleobindin-2 OS=Homo sapiens OX=9606 GN=NUCB2 PE=1 SV=3
NUCKS1	Q9H1E3	Nuclear ubiquitous casein and cyclin-dependent kinase substrate 1 OS=Homo sapiens OX=9606 GN=NUCKS1 PE=1 SV=1
NUDT5	Q9UKK9	ADP-sugar pyrophosphatase OS=Homo sapiens OX=9606 GN=NUDT5 PE=1 SV=1
NUTF2	P61970	Nuclear transport factor 2 OS=Homo sapiens OX=9606 GN=NUTF2 PE=1 SV=1
ORM1	P02763	Alpha-1-acid glycoprotein 1 OS=Homo sapiens OX=9606 GN=ORM1 PE=1 SV=2
ORM2	P19652	Alpha-1-acid glycoprotein 2 OS=Homo sapiens OX=9606 GN=ORM2 PE=1 SV=2
OSTF1	Q92882	Osteoclast-stimulating factor 1 OS=Homo sapiens OX=9606 GN=OSTF1 PE=1 SV=2
OXSR1	O95747	Serine/threonine-protein kinase OSR1 OS=Homo sapiens OX=9606 GN=OXSR1 PE=1 SV=1
P0DOX2	P0DOX2	Immunoglobulin alpha-2 heavy chain OS=Homo sapiens OX=9606 PE=1 SV=2
P0DOX3	P0DOX3	Immunoglobulin delta heavy chain OS=Homo sapiens OX=9606 PE=1 SV=1
P0DOX5	P0DOX5	Immunoglobulin gamma-1 heavy chain OS=Homo sapiens OX=9606 PE=1 SV=2
P0DOX7	P0DOX7	Immunoglobulin kappa light chain OS=Homo sapiens OX=9606 PE=1 SV=1
P0DOX8	P0DOX8	Immunoglobulin lambda-1 light chain OS=Homo sapiens OX=9606 PE=1 SV=1
P4HB	P07237	Protein disulfide-isomerase OS=Homo sapiens OX=9606 GN=P4HB PE=1 SV=3
PABPN1	Q86U42	Polyadenylate-binding protein 2 OS=Homo sapiens OX=9606 GN=PABPN1 PE=1 SV=3
PACSIN2	Q9UNF0	Protein kinase C and casein kinase substrate in neurons protein 2 OS=Homo sapiens OX=9606 GN=PACSIN2 PE=1 SV=2
PADI4	Q9UM07	Protein-arginine deiminase type-4 OS=Homo sapiens OX=9606 GN=PADI4 PE=1 SV=2
PAFAH1B3	Q15102	Platelet-activating factor acetylhydrolase IB subunit alpha1 OS=Homo sapiens OX=9606 GN=PAFAH1B3 PE=1 SV=1
PAK2	Q13177	Serine/threonine-protein kinase PAK 2 OS=Homo sapiens OX=9606 GN=PAK2 PE=1 SV=3
PALS1	Q8N3R9	Protein PALS1 OS=Homo sapiens OX=9606 GN=PALS1 PE=1 SV=3

Protein	Accession	Description
PARK7	Q99497	Parkinson disease protein 7 OS=Homo sapiens OX=9606 GN=PARK7 PE=1 SV=2
PARVB	Q9HBI1	Beta-parvin OS=Homo sapiens OX=9606 GN=PARVB PE=1 SV=1
PASD1	Q8IV76	Circadian clock protein PASD1 OS=Homo sapiens OX=9606 GN=PASD1 PE=1 SV=1
PCBP1	Q15365	Poly(rC)-binding protein 1 OS=Homo sapiens OX=9606 GN=PCBP1 PE=1 SV=2
PCBP2	Q15366	Poly(rC)-binding protein 2 OS=Homo sapiens OX=9606 GN=PCBP2 PE=1 SV=1
PCDH1	Q08174	Protocadherin-1 OS=Homo sapiens OX=9606 GN=PCDH1 PE=1 SV=2
PCMT1	P22061	Protein-L-isoaspartate(D-aspartate) O-methyltransferase OS=Homo sapiens OX=9606 GN=PCMT1 PE=1 SV=4
PCNA	P12004	Proliferating cell nuclear antigen OS=Homo sapiens OX=9606 GN=PCNA PE=1 SV=1
PDCD10	Q9BUL8	Programmed cell death protein 10 OS=Homo sapiens OX=9606 GN=PDCD10 PE=1 SV=1
PDCD6IP	Q8WUM4	Programmed cell death 6-interacting protein OS=Homo sapiens OX=9606 GN=PDCD6IP PE=1 SV=1
PDIA3	P30101	Protein disulfide-isomerase A3 OS=Homo sapiens OX=9606 GN=PDIA3 PE=1 SV=4
PDIA4	P13667	Protein disulfide-isomerase A4 OS=Homo sapiens OX=9606 GN=PDIA4 PE=1 SV=2
PDIA6	Q15084	Protein disulfide-isomerase A6 OS=Homo sapiens OX=9606 GN=PDIA6 PE=1 SV=1
PDLIM1	O00151	PDZ and LIM domain protein 1 OS=Homo sapiens OX=9606 GN=PDLIM1 PE=1 SV=4
PEBP1	P30086	Phosphatidylethanolamine-binding protein 1 OS=Homo sapiens OX=9606 GN=PEBP1 PE=1 SV=3
PECAM1	P16284	Platelet endothelial cell adhesion molecule OS=Homo sapiens OX=9606 GN=PECAM1 PE=1 SV=2
PF4	P02776	Platelet factor 4 OS=Homo sapiens OX=9606 GN=PF4 PE=1 SV=2
PFDN1	O60925	Prefoldin subunit 1 OS=Homo sapiens OX=9606 GN=PFDN1 PE=1 SV=2
PFDN6	O15212	Prefoldin subunit 6 OS=Homo sapiens OX=9606 GN=PFDN6 PE=1 SV=1
PFN1	P07737	Profilin-1 OS=Homo sapiens OX=9606 GN=PFN1 PE=1 SV=2
PGAM1	P18669	Phosphoglycerate mutase 1 OS=Homo sapiens OX=9606 GN=PGAM1 PE=1 SV=2
PGD	P52209	6-phosphogluconate dehydrogenase, decarboxylating OS=Homo sapiens OX=9606 GN=PGD PE=1 SV=3
1,00 PGK	P00558	Phosphoglycerate kinase 1 OS=Homo sapiens OX=9606 GN=PGK1 PE=1 SV=3
PGLS	O95336	6-phosphogluconolactonase OS=Homo sapiens OX=9606 GN=PGLS PE=1 SV=2
PGLYRP1	O75594	Peptidoglycan recognition protein 1 OS=Homo sapiens OX=9606 GN=PGLYRP1 PE=1 SV=1

Protein	Accession	Description
PGLYRP2	Q96PD5	N-acetylmuramoyl-L-alanine amidase OS=Homo sapiens OX=9606 GN=PGLYRP2 PE=1 SV=1
PI16	Q6UXB8	Peptidase inhibitor 16 OS=Homo sapiens OX=9606 GN=PI16 PE=1 SV=1
PI4K2A	Q9BTU6	Phosphatidylinositol 4-kinase type 2-alpha OS=Homo sapiens OX=9606 GN=PI4K2A PE=1 SV=1
PIKFYVE	Q9Y2I7	1-phosphatidylinositol 3-phosphate 5-kinase OS=Homo sapiens OX=9606 GN=PIKFYVE PE=1 SV=3
PIN1	Q13526	Peptidyl-prolyl cis-trans isomerase NIMA-interacting 1 OS=Homo sapiens OX=9606 GN=PIN1 PE=1 SV=1
PIP4K2A	P48426	Phosphatidylinositol 5-phosphate 4-kinase type-2 alpha OS=Homo sapiens OX=9606 GN=PIP4K2A PE=1 SV=2
PKLR	P30613	Pyruvate kinase PKLR OS=Homo sapiens OX=9606 GN=PKLR PE=1 SV=2
PKM	P14618	Pyruvate kinase PKM OS=Homo sapiens OX=9606 GN=PKM PE=1 SV=4
PLCXD2	Q0VAA5	PI-PLC X domain-containing protein 2 OS=Homo sapiens OX=9606 GN=PLCXD2 PE=2 SV=1
PLEC	Q15149	Plectin OS=Homo sapiens OX=9606 GN=PLEC PE=1 SV=3
PLEK	P08567	Pleckstrin OS=Homo sapiens OX=9606 GN=PLEK PE=1 SV=3
PLG	P00747	Plasminogen OS=Homo sapiens OX=9606 GN=PLG PE=1 SV=2
PLIN3	O60664	Perilipin-3 OS=Homo sapiens OX=9606 GN=PLIN3 PE=1 SV=3
PLP2	Q04941	Proteolipid protein 2 OS=Homo sapiens OX=9606 GN=PLP2 PE=1 SV=1
PMFBP1	Q8TBY8	Polyamine-modulated factor 1-binding protein 1 OS=Homo sapiens OX=9606 GN=PMFBP1 PE=1 SV=3
PON1	P27169	Serum paraoxonase/arylesterase 1 OS=Homo sapiens OX=9606 GN=PON1 PE=1 SV=3
PPBP	P02775	Platelet basic protein OS=Homo sapiens OX=9606 GN=PPBP PE=1 SV=3
PPIA	P62937	Peptidyl-prolyl cis-trans isomerase A OS=Homo sapiens OX=9606 GN=PPIA PE=1 SV=2
PPIB	P23284	Peptidyl-prolyl cis-trans isomerase B OS=Homo sapiens OX=9606 GN=PPIB PE=1 SV=2
PPM1A	P35813	Protein phosphatase 1A OS=Homo sapiens OX=9606 GN=PPM1A PE=1 SV=1
PPM1B	O75688	Protein phosphatase 1B OS=Homo sapiens OX=9606 GN=PPM1B PE=1 SV=1
PPP1R12A	O14974	Protein phosphatase 1 regulatory subunit 12A OS=Homo sapiens OX=9606 GN=PPP1R12A PE=1 SV=1
PPP1R2	P41236	Protein phosphatase inhibitor 2 OS=Homo sapiens OX=9606 GN=PPP1R2 PE=1 SV=2
PPP1R7	Q15435	Protein phosphatase 1 regulatory subunit 7 OS=Homo sapiens OX=9606 GN=PPP1R7 PE=1 SV=1

Protein	Accession	Description
PPP2R1A	P30153	Serine/threonine-protein phosphatase 2A 65 kDa regulatory subunit A alpha isoform OS=Homo sapiens OX=9606 GN=PPP2R1A PE=1 SV=4
PPP6R1	Q9UPN7	Serine/threonine-protein phosphatase 6 regulatory subunit 1 OS=Homo sapiens OX=9606 GN=PPP6R1 PE=1 SV=5
PRDX1	Q06830	Peroxiredoxin-1 OS=Homo sapiens OX=9606 GN=PRDX1 PE=1 SV=1
PRDX2	P32119	Peroxiredoxin-2 OS=Homo sapiens OX=9606 GN=PRDX2 PE=1 SV=5
PRDX5	P30044	Peroxiredoxin-5, mitochondrial OS=Homo sapiens OX=9606 GN=PRDX5 PE=1 SV=4
PRDX6	P30041	Peroxiredoxin-6 OS=Homo sapiens OX=9606 GN=PRDX6 PE=1 SV=3
PRG4	Q92954	Proteoglycan 4 OS=Homo sapiens OX=9606 GN=PRG4 PE=1 SV=3
PRKCSH	P14314	Glucosidase 2 subunit beta OS=Homo sapiens OX=9606 GN=PRKCSH PE=1 SV=2
PROC	P04070	Vitamin K-dependent protein C OS=Homo sapiens OX=9606 GN=PROC PE=1 SV=1
PROS1	P07225	Vitamin K-dependent protein S OS=Homo sapiens OX=9606 GN=PROS1 PE=1 SV=1
PROZ	P22891	Vitamin K-dependent protein Z OS=Homo sapiens OX=9606 GN=PROZ PE=1 SV=2
PRPS1	P60891	Ribose-phosphate pyrophosphokinase 1 OS=Homo sapiens OX=9606 GN=PRPS1 PE=1 SV=2
PRSS3	P35030	Trypsin-3 OS=Homo sapiens OX=9606 GN=PRSS3 PE=1 SV=2
PRTN3	P24158	Myeloblastin OS=Homo sapiens OX=9606 GN=PRTN3 PE=1 SV=3
PSAP	P07602	Prosaposin OS=Homo sapiens OX=9606 GN=PSAP PE=1 SV=2
PSMA1	P25786	Proteasome subunit alpha type-1 OS=Homo sapiens OX=9606 GN=PSMA1 PE=1 SV=1
PSMA2	P25787	Proteasome subunit alpha type-2 OS=Homo sapiens OX=9606 GN=PSMA2 PE=1 SV=2
PSMA3	P25788	Proteasome subunit alpha type-3 OS=Homo sapiens OX=9606 GN=PSMA3 PE=1 SV=2
PSMA4	P25789	Proteasome subunit alpha type-4 OS=Homo sapiens OX=9606 GN=PSMA4 PE=1 SV=1
PSMA5	P28066	Proteasome subunit alpha type-5 OS=Homo sapiens OX=9606 GN=PSMA5 PE=1 SV=3
PSMA6	P60900	Proteasome subunit alpha type-6 OS=Homo sapiens OX=9606 GN=PSMA6 PE=1 SV=1
PSMA7	O14818	Proteasome subunit alpha type-7 OS=Homo sapiens OX=9606 GN=PSMA7 PE=1 SV=1
PSMB1	P20618	Proteasome subunit beta type-1 OS=Homo sapiens OX=9606 GN=PSMB1 PE=1 SV=2
PSMB2	P49721	Proteasome subunit beta type-2 OS=Homo sapiens OX=9606 GN=PSMB2 PE=1 SV=1

Protein	Accession	Description
PSMB4	P28070	Proteasome subunit beta type-4 OS=Homo sapiens OX=9606 GN=PSMB4 PE=1 SV=4
PSMB5	P28074	Proteasome subunit beta type-5 OS=Homo sapiens OX=9606 GN=PSMB5 PE=1 SV=3
PSMB6	P28072	Proteasome subunit beta type-6 OS=Homo sapiens OX=9606 GN=PSMB6 PE=1 SV=4
PSMB7	Q99436	Proteasome subunit beta type-7 OS=Homo sapiens OX=9606 GN=PSMB7 PE=1 SV=1
PSMC1	P62191	26S proteasome regulatory subunit 4 OS=Homo sapiens OX=9606 GN=PSMC1 PE=1 SV=1
PSMC2	P35998	26S proteasome regulatory subunit 7 OS=Homo sapiens OX=9606 GN=PSMC2 PE=1 SV=3
PSMC3	P17980	26S proteasome regulatory subunit 6A OS=Homo sapiens OX=9606 GN=PSMC3 PE=1 SV=3
PSMC4	P43686	26S proteasome regulatory subunit 6B OS=Homo sapiens OX=9606 GN=PSMC4 PE=1 SV=2
PSMC5	P62195	26S proteasome regulatory subunit 8 OS=Homo sapiens OX=9606 GN=PSMC5 PE=1 SV=1
PSMD1	Q99460	26S proteasome non-ATPase regulatory subunit 1 OS=Homo sapiens OX=9606 GN=PSMD1 PE=1 SV=2
PSMD11	O00231	26S proteasome non-ATPase regulatory subunit 11 OS=Homo sapiens OX=9606 GN=PSMD11 PE=1 SV=3
PSMD2	Q13200	26S proteasome non-ATPase regulatory subunit 2 OS=Homo sapiens OX=9606 GN=PSMD2 PE=1 SV=3
PSMD6	Q15008	26S proteasome non-ATPase regulatory subunit 6 OS=Homo sapiens OX=9606 GN=PSMD6 PE=1 SV=1
PSMD9	O00233	26S proteasome non-ATPase regulatory subunit 9 OS=Homo sapiens OX=9606 GN=PSMD9 PE=1 SV=3
PSME1	Q06323	Proteasome activator complex subunit 1 OS=Homo sapiens OX=9606 GN=PSME1 PE=1 SV=1
PSME2	Q9UL46	Proteasome activator complex subunit 2 OS=Homo sapiens OX=9606 GN=PSME2 PE=1 SV=4
PSMF1	Q92530	Proteasome inhibitor PI31 subunit OS=Homo sapiens OX=9606 GN=PSMF1 PE=1 SV=2
PTBP1	P26599	Polypyrimidine tract-binding protein 1 OS=Homo sapiens OX=9606 GN=PTBP1 PE=1 SV=2
PTGES3	Q15185	Prostaglandin E synthase 3 OS=Homo sapiens OX=9606 GN=PTGES3 PE=1 SV=1
PTMA	P06454	Prothymosin alpha OS=Homo sapiens OX=9606 GN=PTMA PE=1 SV=2
PTMS	P20962	Parathymosin OS=Homo sapiens OX=9606 GN=PTMS PE=1 SV=2
PTPA	Q15257	Serine/threonine-protein phosphatase 2A activator OS=Homo sapiens OX=9606 GN=PTPA PE=1 SV=3
PTPN6	P29350	Tyrosine-protein phosphatase non-receptor type 6 OS=Homo sapiens OX=9606 GN=PTPN6 PE=1 SV=1
PTPRC	P08575	Receptor-type tyrosine-protein phosphatase C OS=Homo sapiens OX=9606 GN=PTPRC PE=1 SV=3

Protein	Accession	Description
PTPRCAP	Q14761	Protein tyrosine phosphatase receptor type C-associated protein OS=Homo sapiens OX=9606 GN=PTPRCAP PE=1 SV=1
PTPRJ	Q12913	Receptor-type tyrosine-protein phosphatase eta OS=Homo sapiens OX=9606 GN=PTPRJ PE=1 SV=3
PTTG1IP	P53801	Pituitary tumor-transforming gene 1 protein-interacting protein OS=Homo sapiens OX=9606 GN=PTTG1IP PE=1 SV=1
PTX3	P26022	Pentraxin-related protein PTX3 OS=Homo sapiens OX=9606 GN=PTX3 PE=1 SV=3
PURA	Q00577	Transcriptional activator protein Pur-alpha OS=Homo sapiens OX=9606 GN=PURA PE=1 SV=2
PYCARD	Q9ULZ3	Apoptosis-associated speck-like protein containing a CARD OS=Homo sapiens OX=9606 GN=PYCARD PE=1 SV=2
PYGL	P06737	Glycogen phosphorylase, liver form OS=Homo sapiens OX=9606 GN=PYGL PE=1 SV=4
RAB10	P61026	Ras-related protein Rab-10 OS=Homo sapiens OX=9606 GN=RAB10 PE=1 SV=1
RAB11B	Q15907	Ras-related protein Rab-11B OS=Homo sapiens OX=9606 GN=RAB11B PE=1 SV=4
RAB14	P61106	Ras-related protein Rab-14 OS=Homo sapiens OX=9606 GN=RAB14 PE=1 SV=4
RAB1A	P62820	Ras-related protein Rab-1A OS=Homo sapiens OX=9606 GN=RAB1A PE=1 SV=3
RAB21	Q9UL25	Ras-related protein Rab-21 OS=Homo sapiens OX=9606 GN=RAB21 PE=1 SV=3
RAB27A	P51159	Ras-related protein Rab-27A OS=Homo sapiens OX=9606 GN=RAB27A PE=1 SV=3
RAB27B	O00194	Ras-related protein Rab-27B OS=Homo sapiens OX=9606 GN=RAB27B PE=1 SV=4
RAB5B	P61020	Ras-related protein Rab-5B OS=Homo sapiens OX=9606 GN=RAB5B PE=1 SV=1
RAB7A	P51149	Ras-related protein Rab-7a OS=Homo sapiens OX=9606 GN=RAB7A PE=1 SV=1
RAB8A	P61006	Ras-related protein Rab-8A OS=Homo sapiens OX=9606 GN=RAB8A PE=1 SV=1
RAC1	P63000	Ras-related C3 botulinum toxin substrate 1 OS=Homo sapiens OX=9606 GN=RAC1 PE=1 SV=1
RAC2	P15153	Ras-related C3 botulinum toxin substrate 2 OS=Homo sapiens OX=9606 GN=RAC2 PE=1 SV=1
RAD23A	P54725	UV excision repair protein RAD23 homolog A OS=Homo sapiens OX=9606 GN=RAD23A PE=1 SV=1
RAD23B	P54727	UV excision repair protein RAD23 homolog B OS=Homo sapiens OX=9606 GN=RAD23B PE=1 SV=1
RALA	P11233	Ras-related protein Ral-A OS=Homo sapiens OX=9606 GN=RALA PE=1 SV=1
RALY	Q9UKM9	RNA-binding protein Raly OS=Homo sapiens OX=9606 GN=RALY PE=1 SV=1
RAN	P62826	GTP-binding nuclear protein Ran OS=Homo sapiens OX=9606 GN=RAN PE=1 SV=3

Protein	Accession	Description
RANBP1	P43487	Ran-specific GTPase-activating protein OS=Homo sapiens OX=9606 GN=RANBP1 PE=1 SV=1
RANGAP1	P46060	Ran GTPase-activating protein 1 OS=Homo sapiens OX=9606 GN=RANGAP1 PE=1 SV=1
RAP1B	P61224	Ras-related protein Rap-1b OS=Homo sapiens OX=9606 GN=RAP1B PE=1 SV=1
RARRES2	Q99969	Retinoic acid receptor responder protein 2 OS=Homo sapiens OX=9606 GN=RARRES2 PE=1 SV=1
RBBP4	Q09028	Histone-binding protein RBBP4 OS=Homo sapiens OX=9606 GN=RBBP4 PE=1 SV=3
RBM25	P49756	RNA-binding protein 25 OS=Homo sapiens OX=9606 GN=RBM25 PE=1 SV=3
RBMX	P38159	RNA-binding motif protein, X chromosome OS=Homo sapiens OX=9606 GN=RBMX PE=1 SV=3
RBP4	P02753	Retinol-binding protein 4 OS=Homo sapiens OX=9606 GN=RBP4 PE=1 SV=3
RETN	Q9HD89	Resistin OS=Homo sapiens OX=9606 GN=RETN PE=1 SV=1
RGS10	O43665	Regulator of G-protein signaling 10 OS=Homo sapiens OX=9606 GN=RGS10 PE=1 SV=3
RGS19	P49795	Regulator of G-protein signaling 19 OS=Homo sapiens OX=9606 GN=RGS19 PE=1 SV=1
RHOA	P61586	Transforming protein RhoA OS=Homo sapiens OX=9606 GN=RHOA PE=1 SV=1
RHOBTB2	Q9BYZ6	Rho-related BTB domain-containing protein 2 OS=Homo sapiens OX=9606 GN=RHOBTB2 PE=1 SV=2
RHOG	P84095	Rho-related GTP-binding protein RhoG OS=Homo sapiens OX=9606 GN=RHOG PE=1 SV=1
RNF11	Q9Y3C5	RING finger protein 11 OS=Homo sapiens OX=9606 GN=RNF11 PE=1 SV=1
RNH1	P13489	Ribonuclease inhibitor OS=Homo sapiens OX=9606 GN=RNH1 PE=1 SV=2
RO60	P10155	RNA-binding protein RO60 OS=Homo sapiens OX=9606 GN=RO60 PE=1 SV=2
RPEL1	Q2QD12	Ribulose-phosphate 3-epimerase-like protein 1 OS=Homo sapiens OX=9606 GN=RPEL1 PE=2 SV=1
RPL10A	P62906	60S ribosomal protein L10a OS=Homo sapiens OX=9606 GN=RPL10A PE=1 SV=2
RPL12	P30050	60S ribosomal protein L12 OS=Homo sapiens OX=9606 GN=RPL12 PE=1 SV=1
RPL13	P26373	60S ribosomal protein L13 OS=Homo sapiens OX=9606 GN=RPL13 PE=1 SV=4
RPL15	P61313	60S ribosomal protein L15 OS=Homo sapiens OX=9606 GN=RPL15 PE=1 SV=2
RPL22	P35268	60S ribosomal protein L22 OS=Homo sapiens OX=9606 GN=RPL22 PE=1 SV=2
RPL27	P61353	60S ribosomal protein L27 OS=Homo sapiens OX=9606 GN=RPL27 PE=1 SV=2
RPL29	P47914	60S ribosomal protein L29 OS=Homo sapiens OX=9606 GN=RPL29 PE=1 SV=2

Protein	Accession	Description
RPL4	P36578	60S ribosomal protein L4 OS=Homo sapiens OX=9606 GN=RPL4 PE=1 SV=5
RPL6	Q02878	60S ribosomal protein L6 OS=Homo sapiens OX=9606 GN=RPL6 PE=1 SV=3
RPL7A	P62424	60S ribosomal protein L7a OS=Homo sapiens OX=9606 GN=RPL7A PE=1 SV=2
RPL8	P62917	60S ribosomal protein L8 OS=Homo sapiens OX=9606 GN=RPL8 PE=1 SV=2
RPLP0	P05388	60S acidic ribosomal protein P0 OS=Homo sapiens OX=9606 GN=RPLP0 PE=1 SV=1
RPLP2	P05387	60S acidic ribosomal protein P2 OS=Homo sapiens OX=9606 GN=RPLP2 PE=1 SV=1
RPS10	P46783	40S ribosomal protein S10 OS=Homo sapiens OX=9606 GN=RPS10 PE=1 SV=1
RPS12	P25398	40S ribosomal protein S12 OS=Homo sapiens OX=9606 GN=RPS12 PE=1 SV=3
RPS14	P62263	40S ribosomal protein S14 OS=Homo sapiens OX=9606 GN=RPS14 PE=1 SV=3
RPS15	P62841	40S ribosomal protein S15 OS=Homo sapiens OX=9606 GN=RPS15 PE=1 SV=2
RPS15A	P62244	40S ribosomal protein S15a OS=Homo sapiens OX=9606 GN=RPS15A PE=1 SV=2
RPS18	P62269	40S ribosomal protein S18 OS=Homo sapiens OX=9606 GN=RPS18 PE=1 SV=3
RPS19	P39019	40S ribosomal protein S19 OS=Homo sapiens OX=9606 GN=RPS19 PE=1 SV=2
RPS20	P60866	40S ribosomal protein S20 OS=Homo sapiens OX=9606 GN=RPS20 PE=1 SV=1
RPS21	P63220	40S ribosomal protein S21 OS=Homo sapiens OX=9606 GN=RPS21 PE=1 SV=1
RPS25	P62851	40S ribosomal protein S25 OS=Homo sapiens OX=9606 GN=RPS25 PE=1 SV=1
RPS27A	P62979	Ubiquitin-40S ribosomal protein S27a OS=Homo sapiens OX=9606 GN=RPS27A PE=1 SV=2
RPS28	P62857	40S ribosomal protein S28 OS=Homo sapiens OX=9606 GN=RPS28 PE=1 SV=1
RPS3A	P61247	40S ribosomal protein S3a OS=Homo sapiens OX=9606 GN=RPS3A PE=1 SV=2
RPS4X	P62701	40S ribosomal protein S4, X isoform OS=Homo sapiens OX=9606 GN=RPS4X PE=1 SV=2
RPS6	P62753	40S ribosomal protein S6 OS=Homo sapiens OX=9606 GN=RPS6 PE=1 SV=1
RPS8	P62241	40S ribosomal protein S8 OS=Homo sapiens OX=9606 GN=RPS8 PE=1 SV=2
RPSA	P08865	40S ribosomal protein SA OS=Homo sapiens OX=9606 GN=RPSA PE=1 SV=4
RSPH14	Q9UHP6	Radial spoke head 14 homolog OS=Homo sapiens OX=9606 GN=RSPH14 PE=1 SV=1

Protein	Accession	Description
RSU1	Q15404	Ras suppressor protein 1 OS=Homo sapiens OX=9606 GN=RSU1 PE=1 SV=3
RTEL1	Q9NZ71	Regulator of telomere elongation helicase 1 OS=Homo sapiens OX=9606 GN=RTEL1 PE=1 SV=2
RTN4	Q9NQC3	Reticulon-4 OS=Homo sapiens OX=9606 GN=RTN4 PE=1 SV=2
RUVBL1	Q9Y265	RuvB-like 1 OS=Homo sapiens OX=9606 GN=RUVBL1 PE=1 SV=1
RUVBL2	Q9Y230	RuvB-like 2 OS=Homo sapiens OX=9606 GN=RUVBL2 PE=1 SV=3
RYR1	P21817	Ryanodine receptor 1 OS=Homo sapiens OX=9606 GN=RYR1 PE=1 SV=3
S100A10	P60903	Protein S100-A10 OS=Homo sapiens OX=9606 GN=S100A10 PE=1 SV=2
S100A11	P31949	Protein S100-A11 OS=Homo sapiens OX=9606 GN=S100A11 PE=1 SV=2
S100A12	P80511	Protein S100-A12 OS=Homo sapiens OX=9606 GN=S100A12 PE=1 SV=2
S100A4	P26447	Protein S100-A4 OS=Homo sapiens OX=9606 GN=S100A4 PE=1 SV=1
S100A6	P06703	Protein S100-A6 OS=Homo sapiens OX=9606 GN=S100A6 PE=1 SV=1
S100A8	P05109	Protein S100-A8 OS=Homo sapiens OX=9606 GN=S100A8 PE=1 SV=1
S100A9	P06702	Protein S100-A9 OS=Homo sapiens OX=9606 GN=S100A9 PE=1 SV=1
S100P	P25815	Protein S100-P OS=Homo sapiens OX=9606 GN=S100P PE=1 SV=2
SAA1	P0DJI8	Serum amyloid A-1 protein OS=Homo sapiens OX=9606 GN=SAA1 PE=1 SV=1
SAA4	P35542	Serum amyloid A-4 protein OS=Homo sapiens OX=9606 GN=SAA4 PE=1 SV=2
SARNP	P82979	SAP domain-containing ribonucleoprotein OS=Homo sapiens OX=9606 GN=SARNP PE=1 SV=3
SARS2	Q9NP81	Serine--tRNA ligase, mitochondrial OS=Homo sapiens OX=9606 GN=SARS2 PE=1 SV=1
SCRN1	Q12765	Secernin-1 OS=Homo sapiens OX=9606 GN=SCRN1 PE=1 SV=2
SDC4	P31431	Syndecan-4 OS=Homo sapiens OX=9606 GN=SDC4 PE=1 SV=2
SDCBP	O00560	Syntenin-1 OS=Homo sapiens OX=9606 GN=SDCBP PE=1 SV=1
SEC22B	O75396	Vesicle-trafficking protein SEC22b OS=Homo sapiens OX=9606 GN=SEC22B PE=1 SV=5
SELENOP	P49908	Selenoprotein P OS=Homo sapiens OX=9606 GN=SELENOP PE=1 SV=3
SELL	P14151	L-selectin OS=Homo sapiens OX=9606 GN=SELL PE=1 SV=2
SELP	P16109	P-selectin OS=Homo sapiens OX=9606 GN=SELP PE=1 SV=3

Protein	Accession	Description
SELPLG	Q14242	P-selectin glycoprotein ligand 1 OS=Homo sapiens OX=9606 GN=SELPLG PE=1 SV=1
SEMA7A	O75326	Semaphorin-7A OS=Homo sapiens OX=9606 GN=SEMA7A PE=1 SV=1
SEPTIN6	Q14141	Septin-6 OS=Homo sapiens OX=9606 GN=SEPTIN6 PE=1 SV=4
SERPINA1	P01009	Alpha-1-antitrypsin OS=Homo sapiens OX=9606 GN=SERPINA1 PE=1 SV=3
SERPINA3	P01011	Alpha-1-antichymotrypsin OS=Homo sapiens OX=9606 GN=SERPINA3 PE=1 SV=2
SERPINA6	P08185	Corticosteroid-binding globulin OS=Homo sapiens OX=9606 GN=SERPINA6 PE=1 SV=1
SERPINB1	P30740	Leukocyte elastase inhibitor OS=Homo sapiens OX=9606 GN=SERPINB1 PE=1 SV=1
SERPINC1	P01008	Antithrombin-III OS=Homo sapiens OX=9606 GN=SERPINC1 PE=1 SV=1
SERPIND1	P05546	Heparin cofactor 2 OS=Homo sapiens OX=9606 GN=SERPIND1 PE=1 SV=3
SERPINF2	P08697	Alpha-2-antiplasmin OS=Homo sapiens OX=9606 GN=SERPINF2 PE=1 SV=3
SERPING1	P05155	Plasma protease C1 inhibitor OS=Homo sapiens OX=9606 GN=SERPING1 PE=1 SV=2
SET	Q01105	Protein SET OS=Homo sapiens OX=9606 GN=SET PE=1 SV=3
SF3B3	Q15393	Splicing factor 3B subunit 3 OS=Homo sapiens OX=9606 GN=SF3B3 PE=1 SV=4
SF3B5	Q9BWJ5	Splicing factor 3B subunit 5 OS=Homo sapiens OX=9606 GN=SF3B5 PE=1 SV=1
SFPQ	P23246	Splicing factor, proline- and glutamine-rich OS=Homo sapiens OX=9606 GN=SFPQ PE=1 SV=2
SH3BGRL3	Q9H299	SH3 domain-binding glutamic acid-rich-like protein 3 OS=Homo sapiens OX=9606 GN=SH3BGRL3 PE=1 SV=1
SH3GL1	Q99961	Endophilin-A2 OS=Homo sapiens OX=9606 GN=SH3GL1 PE=1 SV=1
SH3KBP1	Q96B97	SH3 domain-containing kinase-binding protein 1 OS=Homo sapiens OX=9606 GN=SH3KBP1 PE=1 SV=2
SIPA1	Q96FS4	Signal-induced proliferation-associated protein 1 OS=Homo sapiens OX=9606 GN=SIPA1 PE=1 SV=1
SKAP2	O75563	Src kinase-associated phosphoprotein 2 OS=Homo sapiens OX=9606 GN=SKAP2 PE=1 SV=1
SKP1	P63208	S-phase kinase-associated protein 1 OS=Homo sapiens OX=9606 GN=SKP1 PE=1 SV=2
SLC11A2	P49281	Natural resistance-associated macrophage protein 2 OS=Homo sapiens OX=9606 GN=SLC11A2 PE=1 SV=2
SLC12A6	Q9UHW9	Solute carrier family 12 member 6 OS=Homo sapiens OX=9606 GN=SLC12A6 PE=1 SV=2
SLC14A1	Q13336	Urea transporter 1 OS=Homo sapiens OX=9606 GN=SLC14A1 PE=1 SV=2
SLC1A5	Q15758	Neutral amino acid transporter B(0) OS=Homo sapiens OX=9606 GN=SLC1A5 PE=1 SV=2

Protein	Accession	Description
SLC25A5	P05141	ADP/ATP translocase 2 OS=Homo sapiens OX=9606 GN=SLC25A5 PE=1 SV=7
SLC2A1	P11166	Solute carrier family 2, facilitated glucose transporter member 1 OS=Homo sapiens OX=9606 GN=SLC2A1 PE=1 SV=2
SLC2A3	P11169	Solute carrier family 2, facilitated glucose transporter member 3 OS=Homo sapiens OX=9606 GN=SLC2A3 PE=1 SV=1
SLC30A1	Q9Y6M5	Proton-coupled zinc antiporter SLC30A1 OS=Homo sapiens OX=9606 GN=SLC30A1 PE=1 SV=3
SLC3A2	P08195	4F2 cell-surface antigen heavy chain OS=Homo sapiens OX=9606 GN=SLC3A2 PE=1 SV=3
SLC44A2	Q8IWA5	Choline transporter-like protein 2 OS=Homo sapiens OX=9606 GN=SLC44A2 PE=1 SV=3
SLC4A1	P02730	Band 3 anion transport protein OS=Homo sapiens OX=9606 GN=SLC4A1 PE=1 SV=3
SLC7A1	P30825	High affinity cationic amino acid transporter 1 OS=Homo sapiens OX=9606 GN=SLC7A1 PE=1 SV=1
SLC9A3R1	O14745	Na(+)/H(+) exchange regulatory cofactor NHE-RF1 OS=Homo sapiens OX=9606 GN=SLC9A3R1 PE=1 SV=4
SLK	Q9H2G2	STE20-like serine/threonine-protein kinase OS=Homo sapiens OX=9606 GN=SLK PE=1 SV=1
SMG5	Q9UPR3	Nonsense-mediated mRNA decay factor SMG5 OS=Homo sapiens OX=9606 GN=SMG5 PE=1 SV=3
SMIM1	B2RUZ4	Small integral membrane protein 1 OS=Homo sapiens OX=9606 GN=SMIM1 PE=1 SV=1
SMIM5	Q71RC9	Small integral membrane protein 5 OS=Homo sapiens OX=9606 GN=SMIM5 PE=1 SV=2
SNCA	P37840	Alpha-synuclein OS=Homo sapiens OX=9606 GN=SNCA PE=1 SV=1
SNRNP70	P08621	U1 small nuclear ribonucleoprotein 70 kDa OS=Homo sapiens OX=9606 GN=SNRNP70 PE=1 SV=2
SNRPA	P09012	U1 small nuclear ribonucleoprotein A OS=Homo sapiens OX=9606 GN=SNRPA PE=1 SV=3
SNRPB	P14678	Small nuclear ribonucleoprotein-associated proteins B and B' OS=Homo sapiens OX=9606 GN=SNRPB PE=1 SV=2
SNRPD1	P62314	Small nuclear ribonucleoprotein Sm D1 OS=Homo sapiens OX=9606 GN=SNRPD1 PE=1 SV=1
SNRPD2	P62316	Small nuclear ribonucleoprotein Sm D2 OS=Homo sapiens OX=9606 GN=SNRPD2 PE=1 SV=1
SNRPD3	P62318	Small nuclear ribonucleoprotein Sm D3 OS=Homo sapiens OX=9606 GN=SNRPD3 PE=1 SV=1
SNX3	O60493	Sorting nexin-3 OS=Homo sapiens OX=9606 GN=SNX3 PE=1 SV=3
SOD1	P00441	Superoxide dismutase [Cu-Zn] OS=Homo sapiens OX=9606 GN=SOD1 PE=1 SV=2
SPARC	P09486	SPARC OS=Homo sapiens OX=9606 GN=SPARC PE=1 SV=1
SPARCL1	Q14515	SPARC-like protein 1 OS=Homo sapiens OX=9606 GN=SPARCL1 PE=1 SV=2
SPN	P16150	Leukosialin OS=Homo sapiens OX=9606 GN=SPN PE=1 SV=1

Protein	Accession	Description
SPTA1	P02549	Spectrin alpha chain, erythrocytic 1 OS=Homo sapiens OX=9606 GN=SPTA1 PE=1 SV=5
SPTAN1	Q13813	Spectrin alpha chain, non-erythrocytic 1 OS=Homo sapiens OX=9606 GN=SPTAN1 PE=1 SV=3
SPTB	P11277	Spectrin beta chain, erythrocytic OS=Homo sapiens OX=9606 GN=SPTB PE=1 SV=5
SPTBN1	Q01082	Spectrin beta chain, non-erythrocytic 1 OS=Homo sapiens OX=9606 GN=SPTBN1 PE=1 SV=2
SPTBN5	Q9NRC6	Spectrin beta chain, non-erythrocytic 5 OS=Homo sapiens OX=9606 GN=SPTBN5 PE=1 SV=2
SRGN	P10124	Serglycin OS=Homo sapiens OX=9606 GN=SRGN PE=1 SV=3
SRI	P30626	Sorcin OS=Homo sapiens OX=9606 GN=SRI PE=1 SV=1
SRSF3	P84103	Serine/arginine-rich splicing factor 3 OS=Homo sapiens OX=9606 GN=SRSF3 PE=1 SV=1
SSC5D	A1L4H1	Soluble scavenger receptor cysteine-rich domain-containing protein SSC5D OS=Homo sapiens OX=9606 GN=SSC5D PE=1 SV=3
ST13	P50502	Hsc70-interacting protein OS=Homo sapiens OX=9606 GN=ST13 PE=1 SV=2
STAM	Q92783	Signal transducing adapter molecule 1 OS=Homo sapiens OX=9606 GN=STAM PE=1 SV=3
STEAP3	Q658P3	Metalloreductase STEAP3 OS=Homo sapiens OX=9606 GN=STEAP3 PE=1 SV=2
STIP1	P31948	Stress-induced-phosphoprotein 1 OS=Homo sapiens OX=9606 GN=STIP1 PE=1 SV=1
STK26	Q9P289	Serine/threonine-protein kinase 26 OS=Homo sapiens OX=9606 GN=STK26 PE=1 SV=2
STK4	Q13043	Serine/threonine-protein kinase 4 OS=Homo sapiens OX=9606 GN=STK4 PE=1 SV=2
STMN1	P16949	Stathmin OS=Homo sapiens OX=9606 GN=STMN1 PE=1 SV=3
STOM	P27105	Stomatin OS=Homo sapiens OX=9606 GN=STOM PE=1 SV=3
STX12	Q86Y82	Syntaxin-12 OS=Homo sapiens OX=9606 GN=STX12 PE=1 SV=1
STX4	Q12846	Syntaxin-4 OS=Homo sapiens OX=9606 GN=STX4 PE=1 SV=2
STX7	O15400	Syntaxin-7 OS=Homo sapiens OX=9606 GN=STX7 PE=1 SV=4
STX8	Q9UNK0	Syntaxin-8 OS=Homo sapiens OX=9606 GN=STX8 PE=1 SV=2
STXBP2	Q15833	Syntaxin-binding protein 2 OS=Homo sapiens OX=9606 GN=STXBP2 PE=1 SV=2
SWAP70	Q9UH65	Switch-associated protein 70 OS=Homo sapiens OX=9606 GN=SWAP70 PE=1 SV=1
TAGLN2	P37802	Transgelin-2 OS=Homo sapiens OX=9606 GN=TAGLN2 PE=1 SV=3
TALDO1	P37837	Transaldolase OS=Homo sapiens OX=9606 GN=TALDO1 PE=1 SV=2
TBCA	O75347	Tubulin-specific chaperone A OS=Homo sapiens OX=9606 GN=TBCA PE=1 SV=3
TCP1	P17987	T-complex protein 1 subunit alpha OS=Homo sapiens OX=9606 GN=TCP1 PE=1 SV=1

Protein	Accession	Description
TF	P02787	Serotransferrin OS=Homo sapiens OX=9606 GN=TF PE=1 SV=4
TFG	Q92734	Protein TFG OS=Homo sapiens OX=9606 GN=TFG PE=1 SV=2
TFRC	P02786	Transferrin receptor protein 1 OS=Homo sapiens OX=9606 GN=TFRC PE=1 SV=2
TGFB1	P01137	Transforming growth factor beta-1 proprotein OS=Homo sapiens OX=9606 GN=TGFB1 PE=1 SV=2
TGM2	P21980	Protein-glutamine gamma-glutamyltransferase 2 OS=Homo sapiens OX=9606 GN=TGM2 PE=1 SV=2
THBS1	P07996	Thrombospondin-1 OS=Homo sapiens OX=9606 GN=THBS1 PE=1 SV=2
THBS3	P49746	Thrombospondin-3 OS=Homo sapiens OX=9606 GN=THBS3 PE=1 SV=1
THBS4	P35443	Thrombospondin-4 OS=Homo sapiens OX=9606 GN=THBS4 PE=1 SV=2
TKT	P29401	Transketolase OS=Homo sapiens OX=9606 GN=TKT PE=1 SV=3
TLN1	Q9Y490	Talin-1 OS=Homo sapiens OX=9606 GN=TLN1 PE=1 SV=3
TMEM30A	Q9NV96	Cell cycle control protein 50A OS=Homo sapiens OX=9606 GN=TMEM30A PE=1 SV=1
TMEM9	Q9P0T7	Proton-transporting V-type ATPase complex assembly regulator TMEM9 OS=Homo sapiens OX=9606 GN=TMEM9 PE=1 SV=1
TMOD3	Q9NYL9	Tropomodulin-3 OS=Homo sapiens OX=9606 GN=TMOD3 PE=1 SV=1
TMPRSS11A	Q6ZMR5	Transmembrane protease serine 11A OS=Homo sapiens OX=9606 GN=TMPRSS11A PE=1 SV=2
TMSB4X	P62328	Thymosin beta-4 OS=Homo sapiens OX=9606 GN=TMSB4X PE=1 SV=2
TPD52L2	O43399	Tumor protein D54 OS=Homo sapiens OX=9606 GN=TPD52L2 PE=1 SV=2
TPI1	P60174	Triosephosphate isomerase OS=Homo sapiens OX=9606 GN=TPI1 PE=1 SV=4
TPM1	P09493	Tropomyosin alpha-1 chain OS=Homo sapiens OX=9606 GN=TPM1 PE=1 SV=2
TPM3	P06753	Tropomyosin alpha-3 chain OS=Homo sapiens OX=9606 GN=TPM3 PE=1 SV=2
TPM4	P67936	Tropomyosin alpha-4 chain OS=Homo sapiens OX=9606 GN=TPM4 PE=1 SV=3
TPP2	P29144	Tripeptidyl-peptidase 2 OS=Homo sapiens OX=9606 GN=TPP2 PE=1 SV=4
TPT1	P13693	Translationally-controlled tumor protein OS=Homo sapiens OX=9606 GN=TPT1 PE=1 SV=1
TREML1	Q86YW5	Trem-like transcript 1 protein OS=Homo sapiens OX=9606 GN=TREML1 PE=1 SV=2
TSG101	Q99816	Tumor susceptibility gene 101 protein OS=Homo sapiens OX=9606 GN=TSG101 PE=1 SV=2

Protein	Accession	Description
TSTD1	Q8NFU3	Thiosulfate:glutathione sulfurtransferase OS=Homo sapiens OX=9606 GN=TSTD1 PE=1 SV=3
TTR	P02766	Transthyretin OS=Homo sapiens OX=9606 GN=TTR PE=1 SV=1
TUBA1A	Q71U36	Tubulin alpha-1A chain OS=Homo sapiens OX=9606 GN=TUBA1A PE=1 SV=1
TUBA3D	P0DPH8	Tubulin alpha-3D chain OS=Homo sapiens OX=9606 GN=TUBA3D PE=1 SV=1
TUBA4A	P68366	Tubulin alpha-4A chain OS=Homo sapiens OX=9606 GN=TUBA4A PE=1 SV=1
TUBB	P07437	Tubulin beta chain OS=Homo sapiens OX=9606 GN=TUBB PE=1 SV=2
TUBB1	Q9H4B7	Tubulin beta-1 chain OS=Homo sapiens OX=9606 GN=TUBB1 PE=1 SV=1
TUBB4B	P68371	Tubulin beta-4B chain OS=Homo sapiens OX=9606 GN=TUBB4B PE=1 SV=1
TXN	P10599	Thioredoxin OS=Homo sapiens OX=9606 GN=TXN PE=1 SV=3
TXNL1	O43396	Thioredoxin-like protein 1 OS=Homo sapiens OX=9606 GN=TXNL1 PE=1 SV=3
TYMP	P19971	Thymidine phosphorylase OS=Homo sapiens OX=9606 GN=TYMP PE=1 SV=2
UBA1	P22314	Ubiquitin-like modifier-activating enzyme 1 OS=Homo sapiens OX=9606 GN=UBA1 PE=1 SV=3
UBE2K	P61086	Ubiquitin-conjugating enzyme E2 K OS=Homo sapiens OX=9606 GN=UBE2K PE=1 SV=3
UBE2L3	P68036	Ubiquitin-conjugating enzyme E2 L3 OS=Homo sapiens OX=9606 GN=UBE2L3 PE=1 SV=1
UBE2N	P61088	Ubiquitin-conjugating enzyme E2 N OS=Homo sapiens OX=9606 GN=UBE2N PE=1 SV=1
UBE2O	Q9C0C9	(E3-independent) E2 ubiquitin-conjugating enzyme OS=Homo sapiens OX=9606 GN=UBE2O PE=1 SV=3
UBE2V1	Q13404	Ubiquitin-conjugating enzyme E2 variant 1 OS=Homo sapiens OX=9606 GN=UBE2V1 PE=1 SV=2
UBN2	Q6ZU65	Ubinuclein-2 OS=Homo sapiens OX=9606 GN=UBN2 PE=1 SV=2
UBXN11	Q5T124	UBX domain-containing protein 11 OS=Homo sapiens OX=9606 GN=UBXN11 PE=1 SV=2
UCHL3	P15374	Ubiquitin carboxyl-terminal hydrolase isozyme L3 OS=Homo sapiens OX=9606 GN=UCHL3 PE=1 SV=1
UGP2	Q16851	UTP--glucose-1-phosphate uridylyltransferase OS=Homo sapiens OX=9606 GN=UGP2 PE=1 SV=5
USO1	O60763	General vesicular transport factor p115 OS=Homo sapiens OX=9606 GN=USO1 PE=1 SV=2
USP14	P54578	Ubiquitin carboxyl-terminal hydrolase 14 OS=Homo sapiens OX=9606 GN=USP14 PE=1 SV=3
USP15	Q9Y4E8	Ubiquitin carboxyl-terminal hydrolase 15 OS=Homo sapiens OX=9606 GN=USP15 PE=1 SV=3

Protein	Accession	Description
USP5	P45974	Ubiquitin carboxyl-terminal hydrolase 5 OS=Homo sapiens OX=9606 GN=USP5 PE=1 SV=2
UTRN	P46939	Utrophin OS=Homo sapiens OX=9606 GN=UTRN PE=1 SV=2
VAMP3	Q15836	Vesicle-associated membrane protein 3 OS=Homo sapiens OX=9606 GN=VAMP3 PE=1 SV=3
VAMP8	Q9BV40	Vesicle-associated membrane protein 8 OS=Homo sapiens OX=9606 GN=VAMP8 PE=1 SV=1
VASP	P50552	Vasodilator-stimulated phosphoprotein OS=Homo sapiens OX=9606 GN=VASP PE=1 SV=3
VAT1	Q99536	Synaptic vesicle membrane protein VAT-1 homolog OS=Homo sapiens OX=9606 GN=VAT1 PE=1 SV=2
VCAN	P13611	Versican core protein OS=Homo sapiens OX=9606 GN=VCAN PE=1 SV=3
VCL	P18206	Vinculin OS=Homo sapiens OX=9606 GN=VCL PE=1 SV=4
VCP	P55072	Transitional endoplasmic reticulum ATPase OS=Homo sapiens OX=9606 GN=VCP PE=1 SV=4
VIM	P08670	Vimentin OS=Homo sapiens OX=9606 GN=VIM PE=1 SV=4
2,00 VNN	O95498	Pantetheine hydrolase VNN2 OS=Homo sapiens OX=9606 GN=VNN2 PE=1 SV=3
VPS37C	A5D8V6	Vacuolar protein sorting-associated protein 37C OS=Homo sapiens OX=9606 GN=VPS37C PE=1 SV=2
VPS4A	Q9UN37	Vacuolar protein sorting-associated protein 4A OS=Homo sapiens OX=9606 GN=VPS4A PE=1 SV=1
VSIR	Q9H7M9	V-type immunoglobulin domain-containing suppressor of T-cell activation OS=Homo sapiens OX=9606 GN=VSIR PE=1 SV=3
VTA1	Q9NP79	Vacuolar protein sorting-associated protein VTA1 homolog OS=Homo sapiens OX=9606 GN=VTA1 PE=1 SV=1
VTI1B	Q9UEU0	Vesicle transport through interaction with t-SNAREs homolog 1B OS=Homo sapiens OX=9606 GN=VTI1B PE=1 SV=3
VTN	P04004	Vitronectin OS=Homo sapiens OX=9606 GN=VTN PE=1 SV=1
VWF	P04275	von Willebrand factor OS=Homo sapiens OX=9606 GN=VWF PE=1 SV=4
WAS	P42768	Actin nucleation-promoting factor WAS OS=Homo sapiens OX=9606 GN=WAS PE=1 SV=4
WASF2	Q9Y6W5	Actin-binding protein WASF2 OS=Homo sapiens OX=9606 GN=WASF2 PE=1 SV=3
WDR1	O75083	WD repeat-containing protein 1 OS=Homo sapiens OX=9606 GN=WDR1 PE=1 SV=4
XRCC5	P13010	X-ray repair cross-complementing protein 5 OS=Homo sapiens OX=9606 GN=XRCC5 PE=1 SV=3
XRCC6	P12956	X-ray repair cross-complementing protein 6 OS=Homo sapiens OX=9606 GN=XRCC6 PE=1 SV=2
XRN1	Q8IZH2	5'-3' exoribonuclease 1 OS=Homo sapiens OX=9606 GN=XRN1 PE=1 SV=1
YBX1	P67809	Y-box-binding protein 1 OS=Homo sapiens OX=9606 GN=YBX1 PE=1 SV=3
YWHAB	P31946	14-3-3 protein beta/alpha OS=Homo sapiens OX=9606 GN=YWHAB PE=1 SV=3

Protein	Accession	Description
YWHAE	P62258	14-3-3 protein epsilon OS=Homo sapiens OX=9606 GN=YWHAE PE=1 SV=1
YWHAG	P61981	14-3-3 protein gamma OS=Homo sapiens OX=9606 GN=YWHAG PE=1 SV=2
YWHAH	Q04917	14-3-3 protein eta OS=Homo sapiens OX=9606 GN=YWHAH PE=1 SV=4
YWHAQ	P27348	14-3-3 protein theta OS=Homo sapiens OX=9606 GN=YWHAQ PE=1 SV=1
YWHAZ	P63104	14-3-3 protein zeta/delta OS=Homo sapiens OX=9606 GN=YWHAZ PE=1 SV=1
ZYX	Q15942	Zyxin OS=Homo sapiens OX=9606 GN=ZYX PE=1 SV=1

Supplemental Table 3. Proteins only detected in EVS derived from H-RBCs by LC-MS/MS

Name	Accession	Description
ACAT2	Q9BWD1	Acetyl-CoA acetyltransferase, cytosolic OS=Homo sapiens OX=9606 GN=ACAT2 PE=1 SV=2
ADGRG3	Q86Y34	Adhesion G protein-coupled receptor G3 OS=Homo sapiens OX=9606 GN=ADGRG3 PE=1 SV=1
AKR1C3	P42330	Aldo-keto reductase family 1 member C3 OS=Homo sapiens OX=9606 GN=AKR1C3 PE=1 SV=4
ALOX5AP	P20292	Arachidonate 5-lipoxygenase-activating protein OS=Homo sapiens OX=9606 GN=ALOX5AP PE=1 SV=2
ARHGAP30	Q7Z6I6	Rho GTPase-activating protein 30 OS=Homo sapiens OX=9606 GN=ARHGAP30 PE=1 SV=3
ATP5F1B	P06576	ATP synthase subunit beta, mitochondrial OS=Homo sapiens OX=9606 GN=ATP5F1B PE=1 SV=3
ATP6V1D	Q9Y5K8	V-type proton ATPase subunit D OS=Homo sapiens OX=9606 GN=ATP6V1D PE=1 SV=1
ATP6V1F	Q16864	V-type proton ATPase subunit F OS=Homo sapiens OX=9606 GN=ATP6V1F PE=1 SV=2
BROX	Q5VW32	BRO1 domain-containing protein BROX OS=Homo sapiens OX=9606 GN=BROX PE=1 SV=1
C5	P01031	Complement C5 OS=Homo sapiens OX=9606 GN=C5 PE=1 SV=4
CD151	P48509	CD151 antigen OS=Homo sapiens OX=9606 GN=CD151 PE=1 SV=3
CDH5	P33151	Cadherin-5 OS=Homo sapiens OX=9606 GN=CDH5 PE=1 SV=5
CHMP6	Q96FZ7	Charged multivesicular body protein 6 OS=Homo sapiens OX=9606 GN=CHMP6 PE=1 SV=3
CKB	P12277	Creatine kinase B-type OS=Homo sapiens OX=9606 GN=CKB PE=1 SV=1
COMP	P49747	Cartilage oligomeric matrix protein OS=Homo sapiens OX=9606 GN=COMP PE=1 SV=2
COPG2	Q9UBF2	Coatomer subunit gamma-2 OS=Homo sapiens OX=9606 GN=COPG2 PE=1 SV=1
CORO7	P57737	Coronin-7 OS=Homo sapiens OX=9606 GN=CORO7 PE=1 SV=2
CROCC2	H7BZ55	Ciliary rootlet coiled-coil protein 2 OS=Homo sapiens OX=9606 GN=CROCC2 PE=1 SV=4
CTSD	P07339	Cathepsin D OS=Homo sapiens OX=9606 GN=CTSD PE=1 SV=1
DAAM1	Q9Y4D1	Disheveled-associated activator of morphogenesis 1 OS=Homo sapiens OX=9606 GN=DAAM1 PE=1 SV=2
DENR	O43583	Density-regulated protein OS=Homo sapiens OX=9606 GN=DENR PE=1 SV=2
DNAJB1	P25685	DnaJ homolog subfamily B member 1 OS=Homo sapiens OX=9606 GN=DNAJB1 PE=1 SV=4
DSTN	P60981	Destrin OS=Homo sapiens OX=9606 GN=DSTN PE=1 SV=3
EFEMP1	Q12805	EGF-containing fibulin-like extracellular matrix protein 1 OS=Homo sapiens OX=9606 GN=EFEMP1 PE=1 SV=2
EHD3	Q9NZN3	EH domain-containing protein 3 OS=Homo sapiens OX=9606 GN=EHD3 PE=1 SV=2
ELOB	Q15370	Elongin-B OS=Homo sapiens OX=9606 GN=ELOB PE=1 SV=1

Name	Accession	Description
EPHA2	P29317	Ephrin type-A receptor 2 OS=Homo sapiens OX=9606 GN=EPHA2 PE=1 SV=2
ERP44	Q9BS26	Endoplasmic reticulum resident protein 44 OS=Homo sapiens OX=9606 GN=ERP44 PE=1 SV=1
FAS	P25445	Tumor necrosis factor receptor superfamily member 6 OS=Homo sapiens OX=9606 GN=FAS PE=1 SV=1
FHL1	Q13642	Four and a half LIM domains protein 1 OS=Homo sapiens OX=9606 GN=FHL1 PE=1 SV=4
FTH1	P02794	Ferritin heavy chain OS=Homo sapiens OX=9606 GN=FTH1 PE=1 SV=2
GPC3	P51654	Glypican-3 OS=Homo sapiens OX=9606 GN=GPC3 PE=1 SV=1
H2BC18	Q5QNW6	Histone H2B type 2-F OS=Homo sapiens OX=9606 GN=H2BC18 PE=1 SV=3
HAGH	Q16775	Hydroxyacylglutathione hydrolase, mitochondrial OS=Homo sapiens OX=9606 GN=HAGH PE=1 SV=2
HUWE1	Q7Z6Z7	E3 ubiquitin-protein ligase HUWE1 OS=Homo sapiens OX=9606 GN=HUWE1 PE=1 SV=3
HYOU1	Q9Y4L1	Hypoxia up-regulated protein 1 OS=Homo sapiens OX=9606 GN=HYOU1 PE=1 SV=1
IGFBP3	P17936	Insulin-like growth factor-binding protein 3 OS=Homo sapiens OX=9606 GN=IGFBP3 PE=1 SV=2
IGKV1D-33	P01593	Immunoglobulin kappa variable 1D-33 OS=Homo sapiens OX=9606 GN=IGKV1D-33 PE=1 SV=2
IGKV4-1	P06312	Immunoglobulin kappa variable 4-1 OS=Homo sapiens OX=9606 GN=IGKV4-1 PE=1 SV=1
LAMP1	P11279	Lysosome-associated membrane glycoprotein 1 OS=Homo sapiens OX=9606 GN=LAMP1 PE=1 SV=3
LRG1	P02750	Leucine-rich alpha-2-glycoprotein OS=Homo sapiens OX=9606 GN=LRG1 PE=1 SV=2
3,00 LSM	P62310	U6 snRNA-associated Sm-like protein LSm3 OS=Homo sapiens OX=9606 GN=LSM3 PE=1 SV=2
MYO1G	B0I1T2	Unconventional myosin-Ig OS=Homo sapiens OX=9606 GN=MYO1G PE=1 SV=2
NASP	P49321	Nuclear autoantigenic sperm protein OS=Homo sapiens OX=9606 GN=NASP PE=1 SV=2
NEXN	Q0ZGT2	Nexilin OS=Homo sapiens OX=9606 GN=NEXN PE=1 SV=1
NPTN	Q9Y639	Neuroplastin OS=Homo sapiens OX=9606 GN=NPTN PE=1 SV=2
NRP1	O14786	Neuropilin-1 OS=Homo sapiens OX=9606 GN=NRP1 PE=1 SV=3
PA2G4	Q9UQ80	Proliferation-associated protein 2G4 OS=Homo sapiens OX=9606 GN=PA2G4 PE=1 SV=3
PCSK6	P29122	Proprotein convertase subtilisin/kexin type 6 OS=Homo sapiens OX=9606 GN=PCSK6 PE=1 SV=1
PFDN4	Q9NQP4	Prefoldin subunit 4 OS=Homo sapiens OX=9606 GN=PFDN4 PE=1 SV=1
PLVAP	Q9BX97	Plasmalemma vesicle-associated protein OS=Homo sapiens OX=9606 GN=PLVAP PE=1 SV=1
PPA1	Q15181	Inorganic pyrophosphatase OS=Homo sapiens OX=9606 GN=PPA1 PE=1 SV=2

Name	Accession	Description
PSMB8	P28062	Proteasome subunit beta type-8 OS=Homo sapiens OX=9606 GN=PSMB8 PE=1 SV=3
PTPN11	Q06124	Tyrosine-protein phosphatase non-receptor type 11 OS=Homo sapiens OX=9606 GN=PTPN11 PE=1 SV=3
RAB5C	P51148	Ras-related protein Rab-5C OS=Homo sapiens OX=9606 GN=RAB5C PE=1 SV=2
RAB6A	P20340	Ras-related protein Rab-6A OS=Homo sapiens OX=9606 GN=RAB6A PE=1 SV=3
RAP2B	P61225	Ras-related protein Rap-2b OS=Homo sapiens OX=9606 GN=RAP2B PE=1 SV=1
RDX	P35241	Radixin OS=Homo sapiens OX=9606 GN=RDX PE=1 SV=1
RNASE3	P12724	Eosinophil cationic protein OS=Homo sapiens OX=9606 GN=RNASE3 PE=1 SV=2
RPL39	P62891	60S ribosomal protein L39 OS=Homo sapiens OX=9606 GN=RPL39 PE=1 SV=2
RPS26	P62854	40S ribosomal protein S26 OS=Homo sapiens OX=9606 GN=RPS26 PE=1 SV=3
SAFB	Q15424	Scaffold attachment factor B1 OS=Homo sapiens OX=9606 GN=SAFB PE=1 SV=4
SBNO1	A3KN83	Protein strawberry notch homolog 1 OS=Homo sapiens OX=9606 GN=SBNO1 PE=1 SV=1
SMARCA2	P51531	Probable global transcription activator SNF2L2 OS=Homo sapiens OX=9606 GN=SMARCA2 PE=1 SV=2
SNAP23	O00161	Synaptosomal-associated protein 23 OS=Homo sapiens OX=9606 GN=SNAP23 PE=1 SV=1
SNRPE	P62304	Small nuclear ribonucleoprotein E OS=Homo sapiens OX=9606 GN=SNRPE PE=1 SV=1
SOD3	P08294	Extracellular superoxide dismutase [Cu-Zn] OS=Homo sapiens OX=9606 GN=SOD3 PE=1 SV=2
SRP9	P49458	Signal recognition particle 9 kDa protein OS=Homo sapiens OX=9606 GN=SRP9 PE=1 SV=2
STX11	O75558	Syntaxin-11 OS=Homo sapiens OX=9606 GN=STX11 PE=1 SV=1
SUSD5	O60279	Sushi domain-containing protein 5 OS=Homo sapiens OX=9606 GN=SUSD5 PE=1 SV=3
SYNCRIP	O60506	Heterogeneous nuclear ribonucleoprotein Q OS=Homo sapiens OX=9606 GN=SYNCRIP PE=1 SV=2
TIMP1	P01033	Metalloproteinase inhibitor 1 OS=Homo sapiens OX=9606 GN=TIMP1 PE=1 SV=1
TNXB	P22105	Tenascin-X OS=Homo sapiens OX=9606 GN=TNXB PE=1 SV=5
TTLL4	Q14679	Tubulin monoglutamylase TTLL4 OS=Homo sapiens OX=9606 GN=TTLL4 PE=1 SV=2
TWF2	Q6IBS0	Twinfilin-2 OS=Homo sapiens OX=9606 GN=TWF2 PE=1 SV=2
WIPF1	O43516	WAS/WASL-interacting protein family member 1 OS=Homo sapiens OX=9606 GN=WIPF1 PE=1 SV=3
ZRANB2	O95218	Zinc finger Ran-binding domain-containing protein 2 OS=Homo sapiens OX=9606 GN=ZRANB2 PE=1 SV=2

92

Supplemental Table 4. Proteins only detected in EVS derived from T2D-RBCs by LC-MS/MS

Name	Accession	Description
AIF1	P55008	Allograft inflammatory factor 1 OS=Homo sapiens OX=9606 GN=AIF1 PE=1 SV=1
AIMP1	Q12904	Aminoacyl tRNA synthase complex-interacting multifunctional protein 1 OS=Homo sapiens OX=9606 GN=AIMP1 PE=1 SV=2
AKR1B1	P15121	Aldo-keto reductase family 1 member B1 OS=Homo sapiens OX=9606 GN=AKR1B1 PE=1 SV=3
ALDH16A1	Q8IZ83	Aldehyde dehydrogenase family 16 member A1 OS=Homo sapiens OX=9606 GN=ALDH16A1 PE=1 SV=2
ALDH9A1	P49189	4-trimethylaminobutyraldehyde dehydrogenase OS=Homo sapiens OX=9606 GN=ALDH9A1 PE=1 SV=3
ALOX15	P16050	Polyunsaturated fatty acid lipoxygenase ALOX15 OS=Homo sapiens OX=9606 GN=ALOX15 PE=1 SV=3
ALPL	P05186	Alkaline phosphatase, tissue-nonspecific isozyme OS=Homo sapiens OX=9606 GN=ALPL PE=1 SV=4
APMAP	Q9HDC9	Adipocyte plasma membrane-associated protein OS=Homo sapiens OX=9606 GN=APMAP PE=1 SV=2
ARGLU1	Q9NWB6	Arginine and glutamate-rich protein 1 OS=Homo sapiens OX=9606 GN=ARGLU1 PE=1 SV=1
ARHGAP1	Q07960	Rho GTPase-activating protein 1 OS=Homo sapiens OX=9606 GN=ARHGAP1 PE=1 SV=1
AZGP1	P25311	Zinc-alpha-2-glycoprotein OS=Homo sapiens OX=9606 GN=AZGP1 PE=1 SV=2
CALCOCO1	Q9P1Z2	Calcium-binding and coiled-coil domain-containing protein 1 OS=Homo sapiens OX=9606 GN=CALCOCO1 PE=1 SV=2
CD177	Q8N6Q3	CD177 antigen OS=Homo sapiens OX=9606 GN=CD177 PE=1 SV=2
CEACAM8	P31997	Carcinoembryonic antigen-related cell adhesion molecule 8 OS=Homo sapiens OX=9606 GN=CEACAM8 PE=1 SV=2
CNTRL	Q7Z7A1	Centriolin OS=Homo sapiens OX=9606 GN=CNTRL PE=1 SV=2
COPS8	Q99627	COP9 signalosome complex subunit 8 OS=Homo sapiens OX=9606 GN=COPS8 PE=1 SV=1
DNAJC8	O75937	DnaJ homolog subfamily C member 8 OS=Homo sapiens OX=9606 GN=DNAJC8 PE=1 SV=2
DUSP3	P51452	Dual specificity protein phosphatase 3 OS=Homo sapiens OX=9606 GN=DUSP3 PE=1 SV=1
DYNLRB1	Q9NP97	Dynein light chain roadblock-type 1 OS=Homo sapiens OX=9606 GN=DYNLRB1 PE=1 SV=3
EGF	P01133	Pro-epidermal growth factor OS=Homo sapiens OX=9606 GN=EGF PE=1 SV=2
EIF3CL	B5ME19	Eukaryotic translation initiation factor 3 subunit C-like protein OS=Homo sapiens OX=9606 GN=EIF3CL PE=1 SV=1
GCLC	P48506	Glutamate--cysteine ligase catalytic subunit OS=Homo sapiens OX=9606 GN=GCLC PE=1 SV=2
GGT1	P19440	Glutathione hydrolase 1 proenzyme OS=Homo sapiens OX=9606 GN=GGT1 PE=1 SV=2
GLRX3	O76003	Glutaredoxin-3 OS=Homo sapiens OX=9606 GN=GLRX3 PE=1 SV=2

Name	Accession	Description
GOLGB1	Q14789	Golgin subfamily B member 1 OS=Homo sapiens OX=9606 GN=GOLGB1 PE=1 SV=2
GPSM3	Q9Y4H4	G-protein-signaling modulator 3 OS=Homo sapiens OX=9606 GN=GPSM3 PE=1 SV=1
HBZ	P02008	Hemoglobin subunit zeta OS=Homo sapiens OX=9606 GN=HBZ PE=1 SV=2
HDGFL2	Q7Z4V5	Hepatoma-derived growth factor-related protein 2 OS=Homo sapiens OX=9606 GN=HDGFL2 PE=1 SV=1
HK3	P52790	Hexokinase-3 OS=Homo sapiens OX=9606 GN=HK3 PE=1 SV=2
HNRNPAB	Q99729	Heterogeneous nuclear ribonucleoprotein A/B OS=Homo sapiens OX=9606 GN=HNRNPAB PE=1 SV=2
ICAM2	P13598	Intercellular adhesion molecule 2 OS=Homo sapiens OX=9606 GN=ICAM2 PE=1 SV=2
LGALS9B	Q3B8N2	Galectin-9B OS=Homo sapiens OX=9606 GN=LGALS9B PE=1 SV=3
LPCAT2	Q7L5N7	Lysophosphatidylcholine acyltransferase 2 OS=Homo sapiens OX=9606 GN=LPCAT2 PE=1 SV=1
LRRC59	Q96AG4	Leucine-rich repeat-containing protein 59 OS=Homo sapiens OX=9606 GN=LRRC59 PE=1 SV=1
MANF	P55145	Mesencephalic astrocyte-derived neurotrophic factor OS=Homo sapiens OX=9606 GN=MANF PE=1 SV=3
MAPK14	Q16539	Mitogen-activated protein kinase 14 OS=Homo sapiens OX=9606 GN=MAPK14 PE=1 SV=3
MENT	Q9BUN1	Protein MENT OS=Homo sapiens OX=9606 GN=MENT PE=1 SV=1
NNT	Q13423	NAD(P) transhydrogenase, mitochondrial OS=Homo sapiens OX=9606 GN=NNT PE=1 SV=3
PDGFRA	P16234	Platelet-derived growth factor receptor alpha OS=Homo sapiens OX=9606 GN=PDGFRA PE=1 SV=1
PRR4	Q16378	Proline-rich protein 4 OS=Homo sapiens OX=9606 GN=PRR4 PE=1 SV=3
RAB1B	Q9H0U4	Ras-related protein Rab-1B OS=Homo sapiens OX=9606 GN=RAB1B PE=1 SV=1
RALB	P11234	Ras-related protein Ral-B OS=Homo sapiens OX=9606 GN=RALB PE=1 SV=1
RILPL2	Q969X0	RILP-like protein 2 OS=Homo sapiens OX=9606 GN=RILPL2 PE=1 SV=1
RNASE2	P10153	Non-secretory ribonuclease OS=Homo sapiens OX=9606 GN=RNASE2 PE=1 SV=2
ROCK1	Q13464	Rho-associated protein kinase 1 OS=Homo sapiens OX=9606 GN=ROCK1 PE=1 SV=1
RPRD1B	Q9NQG5	Regulation of nuclear pre-mRNA domain-containing protein 1B OS=Homo sapiens OX=9606 GN=RPRD1B PE=1 SV=1
RPS16	P62249	40S ribosomal protein S16 OS=Homo sapiens OX=9606 GN=RPS16 PE=1 SV=2
SGTA	O43765	Small glutamine-rich tetratricopeptide repeat-containing protein alpha OS=Homo sapiens OX=9606 GN=SGTA PE=1 SV=1

Name	Accession	Description
SMARCE1	Q969G3	SWI/SNF-related matrix-associated actin-dependent regulator of chromatin subfamily E member 1 OS=Homo sapiens OX=9606 GN=SMARCE1 PE=1 SV=2
SMC1A	Q14683	Structural maintenance of chromosomes protein 1A OS=Homo sapiens OX=9606 GN=SMC1A PE=1 SV=2
ST8SIA4	Q92187	CMP-N-acetylneuraminate-poly-alpha-2,8-sialyltransferase OS=Homo sapiens OX=9606 GN=ST8SIA4 PE=1 SV=1
STX16	O14662	Syntaxin-16 OS=Homo sapiens OX=9606 GN=STX16 PE=1 SV=3
TMSB10	P63313	Thymosin beta-10 OS=Homo sapiens OX=9606 GN=TMSB10 PE=1 SV=2
TNPO1	Q92973	Transportin-1 OS=Homo sapiens OX=9606 GN=TNPO1 PE=1 SV=2
WARS1	P23381	Tryptophan--tRNA ligase, cytoplasmic OS=Homo sapiens OX=9606 GN=WARS1 PE=1 SV=2
ZBTB8OS	Q8IWT0	Protein archease OS=Homo sapiens OX=9606 GN=ZBTB8OS PE=1 SV=2
