# Supplementary figures and images for: Erythrocyte-derived extracellular vesicles induce endothelial dysfunction through arginase-1 and oxidative stress in type 2 diabetes
Source: J Clin Invest. 2025 Mar 20;135(10):e180900. doi: 10.1172/JCI180900 (PMC12077887; doi:10.1172/JCI180900)

**Figure 4A**

**Arginase-1**

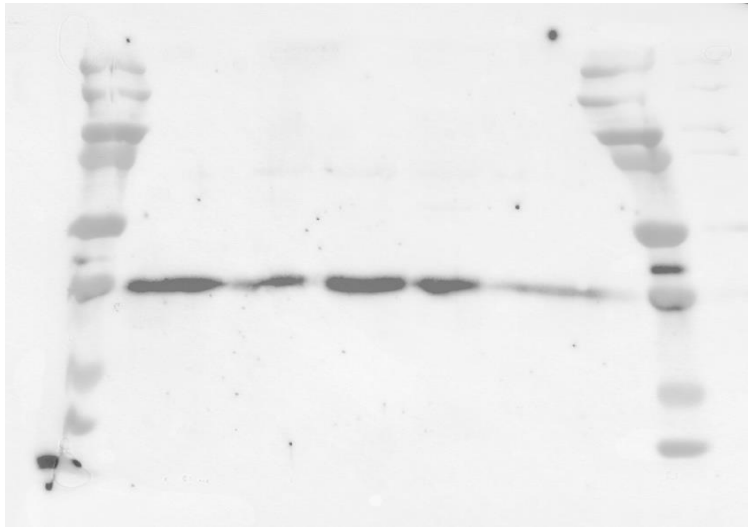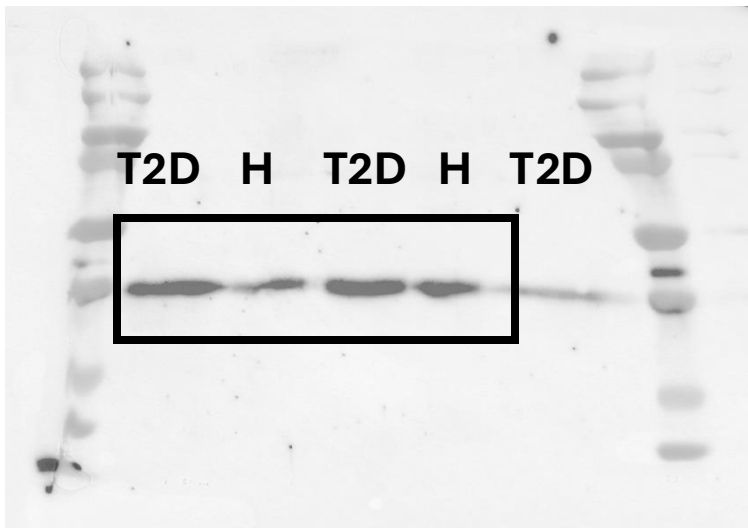

**GAPDH**

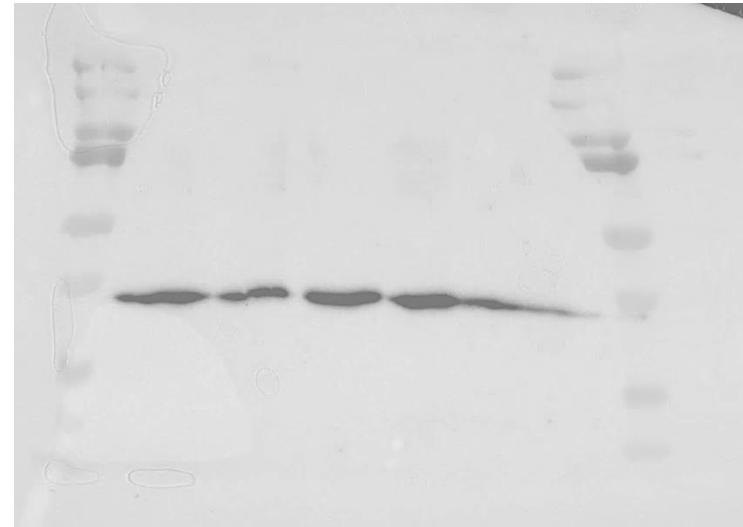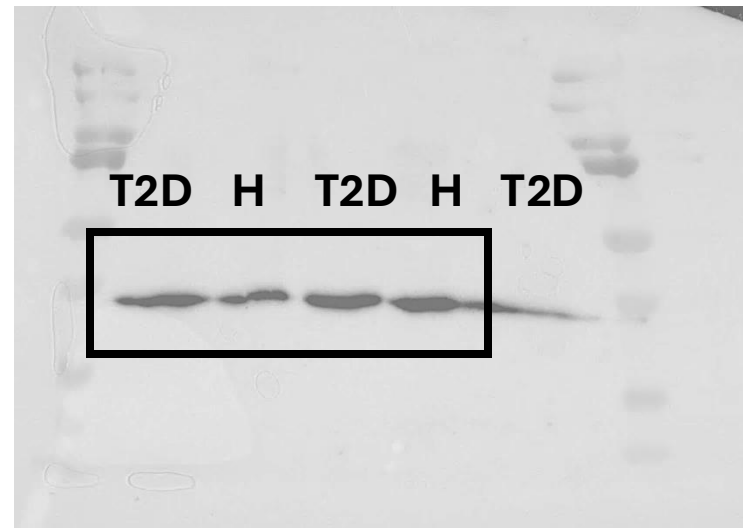

Supplement: Unedited blot and gel images [file jci-135-180900-s278.pdf]
